# Directional Regulation of Zinc Deposition Through Constructing Hierarchical Janus Carbon Matrix with Organic‐Decorated Multi‐Channels

**DOI:** 10.1002/advs.202522959

**Published:** 2026-01-27

**Authors:** Li Gao, Hongli Chen, Dongfang Li, Chen Qian, Chao Yuan, Bin Yu, Jitraporn Vongsvivut, Bernt Johannessen, Lingfei Zhao, Yaojie Lei, Hong Gao, Yufei Zhao, Jinqiang Zhang, Guoxiu Wang, Hao Liu

**Affiliations:** ^1^ Joint International Laboratory on Environmental and Energy Frontier Materials School of Environmental and Chemical Engineering Shanghai University Shanghai P. R. China; ^2^ Centre For Clean Energy Technology University of Technology Sydney Sydney New South Wales Australia; ^3^ State Key Laboratory of Chemical Engineering East China University of Science and Technology Shanghai P. R. China; ^4^ Australian Synchrotron ANSTO Melbourne Victoria Australia

**Keywords:** aqueous zinc‐ion battery, artificial interfacial layer, inorganic‐organic composite layer, Janus structure, porous carbon material, zinc anode

## Abstract

The development of rechargeable aqueous zinc‐ion battery (AZIB) has been hampered by parasitic reactions, despite the low cost, high safety, and environmental friendliness. Here, we design an artificial interfacial layer using a hierarchical Janus carbon matrix on Zn anodes to direct Zn deposition behavior. By covalently anchoring zincophilic organic sulfonic groups within the inner pores of the hierarchical multi‐channel structure of CMK‐5 (CMK‐5/PS), we establish a charge gradient between the grafted inner and unmodified outer surfaces that promotes Zn^2+^ diffusion and deposition. The negatively charged inner surface facilitates fast Zn^2+^ adsorption and uniform nucleation, while the interconnected porous structure facilitates fast Zn^2+^ transport. The confinement effect localizes Zn deposition exclusively within the tubular pores and reduces direct contact with electrolytes, thereby suppressing parasitic reactions and improving the plating/stripping efficiency. As a result, CMK‐5/PS@Zn anode achieves a high Coulombic efficiency of 99.2% and stability over 8000 h. When paired with an NH_4_V_4_O_10_ cathode, the CMK‐5/PS@Zn delivers a high reversible capacity of 213.8 mAh g^−1^ after 1000 cycles at 1 A g^−1^. This work provides a new paradigm for interfacial engineering of Zn metal anodes through carbon‐organic composite architectures, advancing the design of durable and high‐performance AZIB technologies.

## Graphical abstract

A Janus carbon matrix is engineered by covalently anchoring organic sulfonic anions within tubular pores of hierarchical CMK‐5, serving as an interfacial layer for Zn metal anodes. This design establishes a charge gradient for Zn^2+^ diffusion, enabling the directional deposition of confined and uniform Zn inside the pores while suppressing parasitic reactions, thereby enhancing the stability of aqueous Zn batteries.

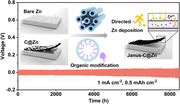

## 1 Introduction

The development of an efficient and environmentally friendly electrochemical energy storage system has become one of the important strategies for the sustainable development of society [1]. Among all the systems, rechargeable aqueous Zn‐ion batteries (AZIBs) have drawn significant attention due to abundant resource reserves, low cost, high theoretical capacity (820 mAh g^−1^ and 5855 mAh cm^−3^), and mild aqueous electrolyte standard hydrogen potential (−0.76 V vs Zn^2+^/Zn) [2, 3, 4, 5, 6]. Nevertheless, the current AZIBs still face severe challenges that limit their commercialization on the industrial scale. In particular, the rather complex interfacial reactions between Zn anodes and electrolytes lead to parasitic reactions such as dendrite growth, hydrogen evolution, and corrosion passivation reactions, resulting in poor stability, fast capacity decay, and short cycle life [7, 8, 9, 10, 11].

To address the challenges of dendrite growth and side reactions in AZIBs, extensive strategies such as interface engineering, structural design, and electrolyte optimization have been explored [12, 13, 14]. In particular, artificial protective layers have shown great promise by regulating Zn^2^^+^ deposition and enhancing the reversibility of Zn metal anodes [15, 16, 17]. Representative examples include inorganic coatings (e.g., CaCO_3_ [18], kaolin [19, 20], TiO_2_ [21], ZrO_2_ [22], MXenes [23]) and organic layers (e.g., Nafion [24], COFs [25]), as well as MOF‐based materials (e.g., ZIF‐7 [26, 27, 28]), which have been reported to regulate Zn^2^^+^ deposition, suppress hydrogen evolution, and mitigate dendrite growth. Among these candidates, carbon‐based materials are particularly effective interfacial layers for stabilizing Zn anodes due to their high conductivity, low cost, and ability to provide large electroactive areas for uniform Zn deposition [29]. Porous carbons (e.g., graphene [30], C_60_ [31], CNTs [32] and hierarchical porous carbon [33]) with high surface area and open nanostructures can regulate Zn^2^^+^ flux, facilitate ion transport, enhance charge transfer, and provide nucleation sites for uniform deposition [34]. However, their weak zincophilicity and possible catalytic activity toward hydrogen evolution can cause uneven Zn deposition, dendrite formation, and reduced Coulombic efficiency.

Various modification strategies, including surface functionalization, heteroatom doping, and incorporation of organic molecules or polymers, have been extensively studied to enhance the zincophilicity of carbon‐based materials, regulate Zn nucleation sites, and suppress dendrite growth during electrochemical cycling [35, 36]. Surface functionalization can introduce specific functional groups that interact favorably with Zn^2+^ ions, thereby improving nucleation uniformity. Heteroatom doping, such as the incorporation of N, O, and S, can create additional active sites and modify the electronic structure of the carbon matrix, promoting more uniform Zn deposition. Organic molecule modification can introduce additional nucleation sites and improve electrolyte wettability, partially promoting uniform Zn deposition [37, 38, 39]. Previous studies have constructed F‐N co‐doped carbon dot/PAN interlayers with both zincophilicity and hydrophobicity, enabling Zn‐induced nucleation and deposition and improving cycling stability [40]. Despite extensive efforts made to modify the carbon materials to improve properties, the non‐directional Zn deposition on the carbon surface is still incapable of suppressing parasitic reactions. Therefore, developing functional carbon materials with hierarchical configuration for efficient interfacial layers to regulate the directional Zn deposition is critical to achieving excellent electrochemical performance of AZIBs.

Herein, we designed a hierarchical Janus CMK‐5 carbon matrix with multi‐channel pores, constructed via a facile organic functionalization strategy to serve as an interfacial layer on Zn metal anodes (CMK‐5/PS@Zn) for high‐performance Zn‐ion batteries. The Janus structure featured the pristine carbon surface to maintain high electronic conductivity and the organic‐sulfonic‐anion‐decorated surface to facilitate ionic transport and enable uniform Zn deposition. In particular, the zincophilic organic sulfonic groups were selectively anchored within the tubular pores of the CMK‐5 matrix, creating a Zn^2+^ gradient to enable directional Zn deposition within the pores. This confined Zn growth in the pores minimized contact with the electrolyte, effectively suppressing parasitic reactions and enhancing Coulombic efficiency. As a result, the CMK‐5/PS interfacial layer exhibited strong zincophilicity, enabling long‐term cycle stability over 8000 h at 0.5 mA cm^−2^ with a smaller voltage hysteresis (∼40 mV) in symmetric cells. The CMK‐5/PS@Zn//Cu asymmetric cell demonstrated a low nucleation overpotential (23.3 mV) and achieved a Coulomb efficiency of 99.2% over 2000 cycles. Furthermore, CMK‐5/PS@Zn//NH_4_V_4_O_10_ full cell showed excellent zinc storage capacity, rate capability, and cycling stability compared to controls. This work presents a new strategy for designing the interfacial layers from the molecular level, opening a new venue to construct dendrite‐free metal anodes for AZIBs.

## 2 Results and Discussion

### 2.1 Theoretical Calculations and Simulations of Janus Carbon Matrix on Zn Anodes to Regulate the Directional Zn Deposition

**FIGURE 1:**
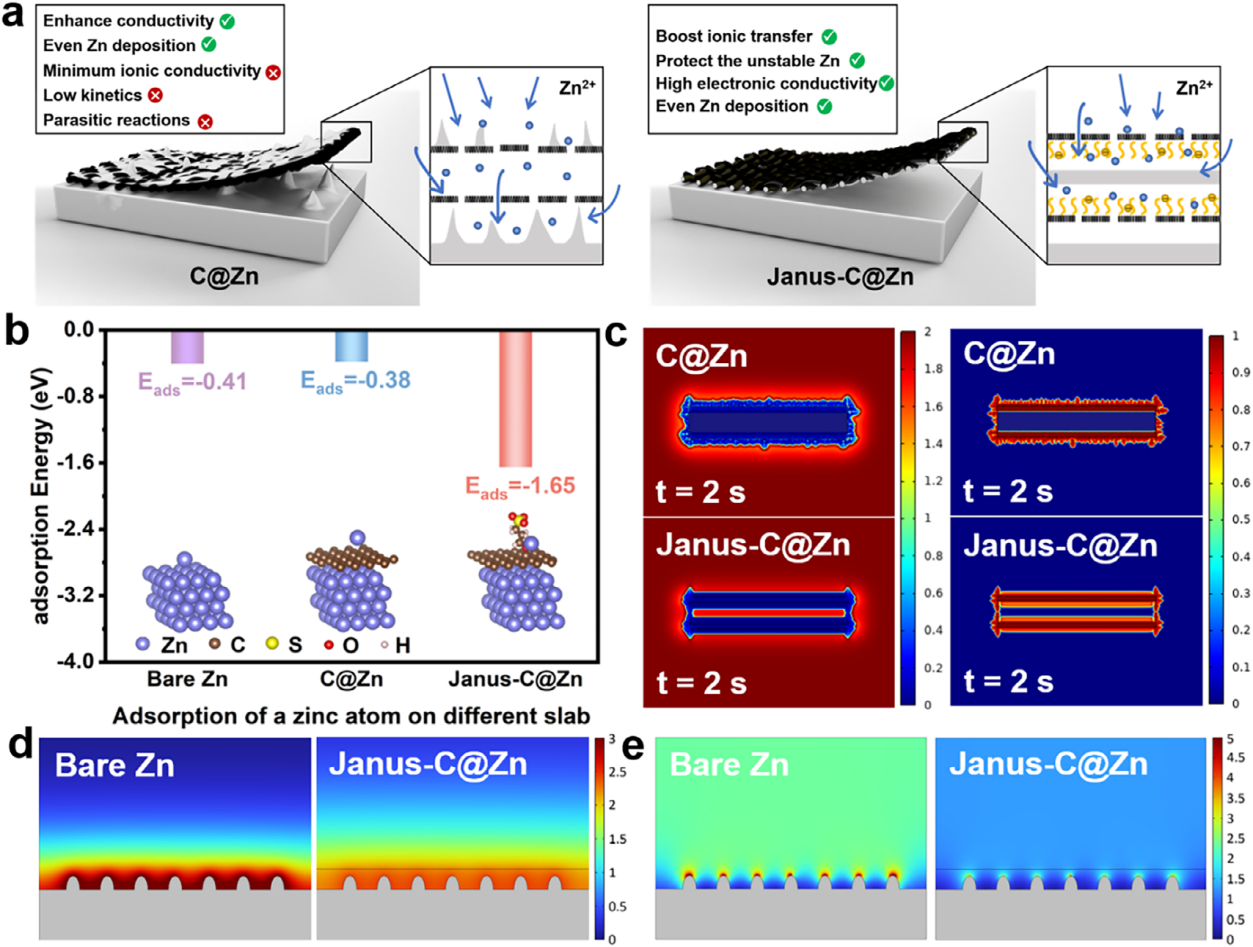
Theoretical calculations and simulations of Janus carbon matrix on Zn anodes to regulate the directional Zn deposition. (a) Schematic illustration of interfacial layers on Zn anodes to enhance properties. (b) Adsorption energy of Zn atoms on different substrate surfaces. (c) COMSOL simulation of Zn^2+^ ion concentration distribution (left) and dendrite growth (right) during Zn deposition on C@Zn and Janus‐C@Zn anode. COMSOL simulation of (d) Zn^2+^ ion concentration distribution and (e) the electric field distribution of C@Zn and Janus‐C@Zn anode.

It is generally understood that the use of untreated zinc foil can easily lead to significant dendrite growth and byproduct accumulation owing to uneven Zn deposition, parasitic reactions, and limited electronic conductivity and ionic diffusion (Figure S1). We posit that the design of a functional surface coating on the bare Zn can alleviate these issues. We first identify that incorporating conducting carbon layers on Zn (C@Zn) can efficiently enhance the in‐plane conductivity while allowing the even distribution of Zn deposition. However, the untreated carbon surface offers the minimum ionic conductivity boost to the double layer on the Zn anode while delivering no protection regarding the deposited Zn layer, exhibiting limited enhancement in kinetics and parasitic reactions (Figure 1a, left panel). Therefore, we propose the introduction of Janus surfaces to the carbon layer (Janus‐C@Zn) to comprehensively alleviate all these issues. The Janus design of the carbon structure by decorating sulfonate ions on the inner surface can further provide boosted ionic transfer of Zn through the negatively charged surface and protect the unstable Zn through a confined deposition pattern, in addition to high electronic conductivity and even Zn deposition (Figure 1a, right panel). Therefore, such a Janus carbon layer design on Zn is ideal for highly efficient Zn‐ion batteries.

We first verified the advances of the Janus design by conducting DFT calculations [41]. As shown in Figure 1b, the adsorption energy of the Zn^2+^ on the Janus‐C@Zn exhibits a significantly reduced value of −1.65 eV, compared to both those of bare Zn (−0.41 eV) and C@Zn (−0.38 eV). This result indicates that Zn is primarily drawn to the negatively charged carbon surface (anion‐decorated surface), rather than the uncharged carbon or Zn surfaces (untreated surface). This provides theoretical guidance on Janus‐C@Zn presenting strong zinc affinity and high capacity for Zn nucleation, which will benefit uniform plating/stripping behavior. This is further verified by the COMSOL simulation of Zn nucleation and deposition, which are usually related to the interfacial electric field distribution [42]. We constructed a simplified physical model of porous channels (negatively charged carbon surface inside and neutral carbon surface outside, Janus‐C@Zn) to simulate the distribution of Zn^2+^ concentration and Zn dendrite growth on Janus carbon (Figure S2a). We constructed the porous channels with untreated carbon both inside and outside for comparison (C@Zn). As shown on C@Zn, the concentration of Zn^2+^ mainly distributes on the outer surface, which increases with time (Figure 1c, top left panel; Figure S3a and Movie S1), leading to the deposition of Zn dendrites primarily outside the channels (Figure 1c, top right panel; Figure S4a and Movie S2). This phenomenon is caused by relatively lower energy for Zn^2+^ accumulating outside than penetrating the inside channels with no modification. The Janus channels in Janus‐C@Zn, however, exhibit a different trend. Due to the high affinity of Zn^2+^ for the negatively charged sulfonic‐anion‐decorated carbon surface, Zn^2+^ tends to diffuse into the inner pores, leading to a quick increase in Zn^2+^ concentration in the pores (Figure 1c, bottom left panel; Figure S3b and Movie S3). This results in a Zn^2+^ gradient that prefers Zn deposition inwards rather than outwards (Figure 1c, bottom right panel; Figure S4b and Movie S4). This design allows the directional Zn growth within the pores of the Janus carbon matrix, which can efficiently suppress the dendrite growth while protecting the deposited Zn in the pores from parasitic reactions. We further explored the Janus carbon layer to control the surface electric field and Zn^2+^ concentration by constructing simplified physical models of COMSOL multiphysics of the bare Zn vs. Janus‐C@Zn (Figure 1d,e; Figure S2b). The bare Zn anode reveals an uneven distribution of the Zn^2+^ concentration and corresponding electric field strength, which shows significant enhancement around the protrusions due to the tip effect. Under such conditions, the growth of Zn dendrites is dramatically promoted. In sharp contrast, the negatively charged matrix coating on Zn effectively homogenized the surface electric field distribution and Zn^2+^ ion concentration, ensuring uniform Zn^2+^ deposition and thereby reducing the chance of Zn dendrite formation. These calculations and simulations provide theoretical insights into designing Janus carbon matrix on Zn anode for stable Zn deposition and suppressed parasitic reactions, providing a new venue for enhancing the performance of AZIBs.

### 2.2 Characterization of Hierarchical Janus CMK‐5/PS

**FIGURE 2:**
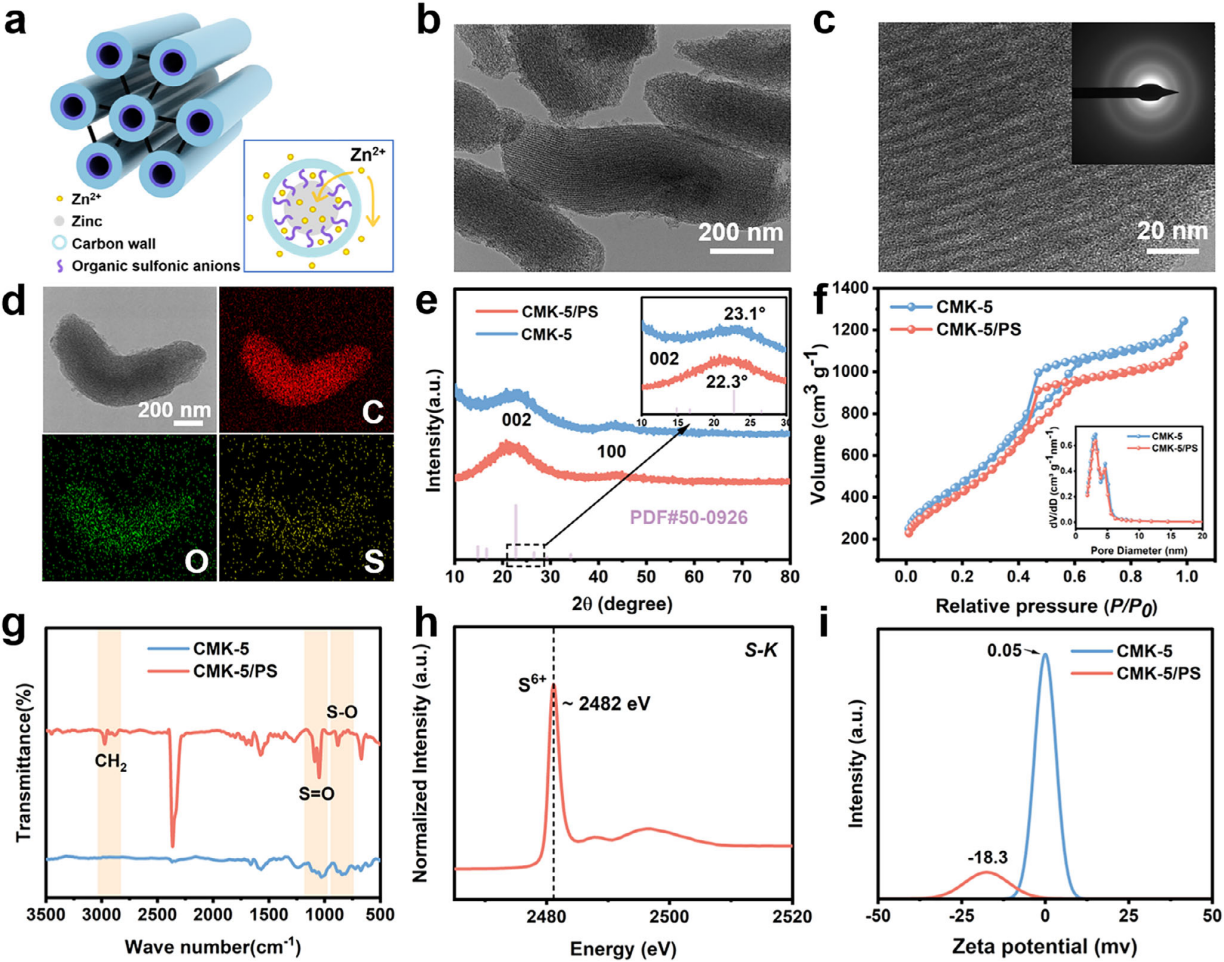
Structural characterizations of hierarchical Janus CMK‐5/PS. (a) Schematic diagram of Zn^2+^ deposition in multi‐level Janus carbon channels in CMK‐5/PS. (b) TEM of CMK‐5/PS. (c) HRTEM of CMK‐5/PS (inset: SAED pattern). (d) EDX element mapping of C, O, and S. (e) XRD patterns and magnified shift patterns of carbon materials with and without organic modifications. (f) Nitrogen adsorption‐desorption isotherms of CMK‐5 and CMK‐5/PS (inset: Diameter distribution). (g) FTIR spectra of CMK‐5/PS and CMK‐5. (h) XANES of S K‐edge. (i) Zeta potential test of CMK‐5/PS and CMK‐5.

With the guidance of theoretical calculations and simulations, we posited that the design of a Janus carbon matrix will be highly beneficial for Zn anodes. Through a template‐assisted synthesis pathway by using mesoporous silica SBA‐15 (Figure S5) followed by a ring‐opening grafting of 1,3‐propanesulfonate before template removal, we synthesized an organic‐anion‐decorated mesoporous CMK‐5 carbon matrix, equipped with a unique multichannel design combining hollow tubes of amorphous carbon backbones (tubule pores) and the voids between adjacent tubes (intertube pores) [43, 44, 45, 46]. The organic sulfonic anions were covalently immobilized in the tubule pores before the removal of the SiO_2_ template, creating the Janus surface to facilitate the Zn deposition (CMK‐5/PS). As shown in Figure 2a and Figure S6, the Janus carbon matrix design can facilitate the directional Zn deposition in the tubule pores owing to the zincophilic nature of the anion‐decorated inner surface conducive to the adsorption and uniform nucleation of Zn^2+^ while facilitating the fast kinetics of Zn^2+^ diffusion through the intertube pores with low energy barrier for Zn^2+^ migration and large surface area. This process leads to the directional uniform Zn deposition in the porous structures of the Janus carbon matrix, which can effectively protect the deposited Zn from parasitic reactions and dendrite growth, resulting in significant improvements in the stability of Zn anodes.

The scanning electron microscope (SEM) images of CMK‐5/PS in Figure S7 exhibits the morphology of rice grains with an average size of ∼500 nm, which has negligible differences from the morphology of CMK‐5. The porous structure of CMK‐5/PS was further characterized by transmission electron microscopy (TEM). As shown in Figure 2b,c, CMK‐5/PS displays the stacking of aligned carbon nanotubes with uniformly arranged rice‐shaped tubular mesoporous pores, similar to CMK‐5 (Figure S8). Each carbon rice particle is composed of two types of mesoporous pores: the intertube porous structure from the removal of the SBA‐15 template and the tubule porous microstructure from the carbon filling in the template. This refers to the fact that decorating the inner surface with anions has no impact on the overall morphology and porous structures, indicating the decoration is restricted to the inner surface rather than outer surface coating. The SAED pattern confirm that the ordered mesoporous carbon is amorphous carbon, and the inner surface is uniformly covered with the organic layer, verified by the uniform distribution of C, O, and S in the EDX element mapping results (Figure 2d; Figure S9). Wide‐angle X‐ray diffraction (XRD) patterns in Figure 2e show two very distinct diffraction peaks centered at 23° and 43°, corresponding to the (002) and (100) faces of the amorphous carbon material in both CMK‐5/PS and CMK‐5. In addition, the (002) diffraction peak of CMK‐5/PS shifts to 22.3° compared to the pure carbon‐based CMK‐5 (23.1°), indicating an increased interlayer distance in the (002) plane, which may be caused by the introduction of bulky organic anions to expand the graphite crystal layer [47, 48]. Expanding the layer spacing of the (002) crystal faces is conducive to providing more space for accommodating more Zn^2+^ while accelerating its diffusion [49]. The nitrogen adsorption‐to‐desorption isotherms (Figure 2f; Table S1) and Raman spectrum (Figure S10) results show that the introduction of organic moieties maintains the unique bimodal pore size structure of CMK‐5 and provides more active sites for the deposition of Zn^2+^.

Figure 2g shows the Fourier transform infrared spectroscopy (FT‐IR) spectra of CMK‐5/PS and CMK‐5. CMK‐5 shows no characteristic absorption band in the measured wavelength range. The CMK‐5/PS shows characteristic peaks of sulfonic groups at three places, namely the SO bond stretching vibration at 879 cm^−1^, and the S═O asymmetric stretching vibration absorption band corresponding to 1049 and 1085 cm^−1^, which are not present in the spectrum of CMK‐5. This verifies the successful grafting of the sulfonic moieties in the CMK‐5 structures. Similarly, the sulfur K‐edge Synchrotron X‐ray absorbing near‐edge structures (XANES) result in Figure 2h shows that the sulfur in CMK‐5/PS exhibits an absorption peak at ∼2482 eV (S^6+^), which shows a similar chemical environment to that of sulfonate/sulfonate salt [50]. This is also consistent with the X‐ray photoelectron spectroscopy (XPS) results of CMK‐5/PS with S signals in addition to the C and O signals observed in CMK‐5 (Figure S11). The overall loading of the sulfonic groups in CMK‐5 is determined to be 3.6 wt.%, calculated by the XPS and thermogravimetric analysis (TGA) (Figure S12). We have also confirmed the decoration of anionic groups on CMK‐5 by conducting zeta potential of CMK‐5/PS, which clearly displays a negatively charged nature, especially when compared to the untreated CMK‐5 (Figure 2i).

We further fabricated the electrodes by CMK‐5/PS slurry drop coating on the surface of Zn foil (Figure S13) to promote the uniform deposition of Zn^2+^ ions and inhibit the formation of dendrites and parasitic reactions. The surface of the drop‐coated CMK‐5/PS coating is relatively flat and uniformly dense, while close observation shows that it is composed of nanocarbon particles with a high specific surface area (Figure S13a,b). The cross‐sectional SEM shows that the drop‐coated layer is tightly attached to Zn and has high physical adhesion, so that the layer will not fall off during the later electrochemical cycle (Figure S14c,d). We further evaluated the hydrophilicity and electrolyte wettability of bare Zn, CMK‐5@Zn, and CMK‐5/PS@Zn anodes by contact angle measurements (Figure S15). CMK‐5/PS@Zn has the smallest contact angle (46.7°), which is lower than that of CMK‐5@Zn (77.3°) and bare Zn foil (100.5°). The improved hydrophilicity of the CMK‐5/PS layer is attributed to the carbon structure of CMK‐5 itself and the hydrophilicity of the sulfonic acid decoration (Figure S16, schematic structure of 1,3‐propanesulfonate) [51, 52], which can promote interfacial connectivity and the transport kinetics of Zn^2+^. As the decoration is specifically located in the pores of CMK‐5, the penetration of Zn^2+^ into the inner pores can be significantly enhanced. These results indicate that the CMK‐5/PS layer will directionally induce Zn deposition in the pores, thereby inhibiting dendrite growth.

### 2.3 Electrochemical Properties of Half Cells using CMK‐5/PS@Zn

**FIGURE 3:**
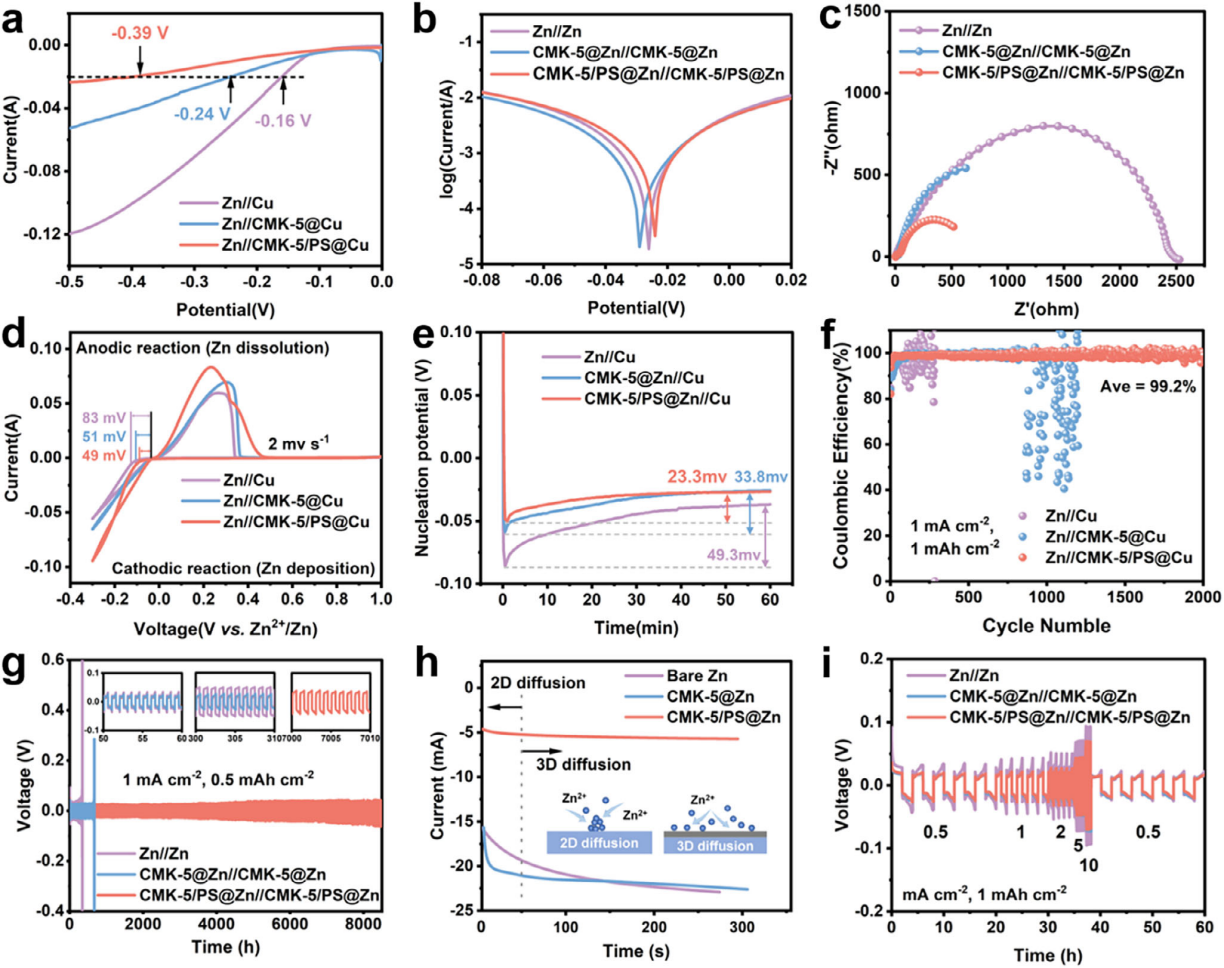
Electrochemical properties of half cells using CMK‐5/PS@Zn. (a) LSV curve of asymmetric cells. (b) Tafel curve test for symmetrical cells. (c) EIS diagram of a symmetric battery in its initial state. (d) CV curve of asymmetric battery at 2mv s^−1^. (e) Nucleation overpotential of Zn deposition on different substrates at 1 mA cm^−2^. (f) Coulomb efficiency of Zn//CMK‐5/PS@Cu, Zn//CMK‐5@Cu, and Zn//Cu asymmetric batteries at 1 mA cm^−2^. (g) Long cycle performance of CMK‐5/PS@Zn//CMK‐5/PS@Zn, CMK‐5@Zn// CMK‐5@Zn, Zn//Cu asymmetric batteries at 1 mA cm^−2^‐0.5 mAh cm^−2^. (h) CA curve at an overpotential of ‐150 mV within Zn||Zn cells (inset: schematic diagrams of 2D and 3D diffusion process of Zn^2+^). (i) Rate performance of symmetric cells at different current densities.

We evaluated the performance of CMK‐5/PS by assembling Zn//Cu asymmetric and Zn//Zn symmetric cells with the prepared electrodes in 2 M ZnSO_4_ electrolyte. We first performed linear sweep voltammetry (LSV) on various electrodes (Zn//interfacial layers@Cu) to investigate the interfacial layers to inhibit hydrogen evolution. The CMK‐5/PS@Cu electrode exhibits the lowest onset potential amongst all electrodes, indicating a much higher capability to suppress the hydrogen evolution reaction (HER) than the CMK‐5@Cu and bare Cu electrodes (Figure 3a). We have also performed the Tafel tests of the symmetric cells, which show the CMK‐5/PS@Zn electrode to have the best corrosion resistance, with the highest corrosion voltage (Figure 3b). In addition, CMK‐5/PS@Zn shows a smooth surface without any cracks after 7 days of aging in the electrolyte, while on the bare Zn electrode, byproducts have been accumulated obviously due to self‐corrosion (Figure S17). These results verify that CMK‐5/PS coating has an outstanding protective effect in suppressing surface corrosion and parasitic reactions of Zn anodes owing to the unique capability of confined growth from Janus structures and fast kinetics from multi‐channels.

The charge transfer and ion diffusion characteristics were studied by electrochemical impedance spectroscopy of the symmetrical cells. As shown in Figure 3c, the CMK‐5/PS@Zn electrode exhibits the smallest charge transfer resistance (R_ct_), referring to a fast charge transfer rate of the CMK‐5/PS interfacial layer. In addition, the CMK‐5/PS@Zn anode exhibits the smallest activation energy (23.35 kJ mol^−1^), which is much smaller than that of the bare Zn and CMK‐5@Zn anodes (40.55 and 35.43 kJ mol^−1^), indicating that the synergistic effects between the introduction of organic matter and multi‐channel design accelerate the charge transfer and desolvation process of Zn^2+^ (Figure S18) [53, 54, 55]. Similar phenomena are also revealed from cyclic voltammetry (CV) curves with these electrodes (Figure 3d; Figures S19–S21). CMK‐5/PS@Zn electrode exhibits the largest peak area and smallest voltage polarization compared to CMK‐5@Zn and bare Zn, and CMK‐5/PS@Zn at all voltage scanning rates, indicating a much enhanced Zn deposition kinetics and reversible plating/stripping process. To further verify the fast kinetics of the CMK‐5/PS@Zn, constant current deposition was also performed to test nucleation overpotential (Figure 3e). The results show that the nucleation overpotential of CMK‐5/PS@Zn (23.3 mV) is significantly lower than that of CMK‐5@Zn (33.8 mV) and bare Zn (49.3 mV), which is consistent with CV and EIS results. The Coulomb efficiency (CE) of Zn plating/stripping is a key parameter in evaluating the performance of metal anodes. As shown in Figure 3f and Figure S22, the Zn//CMK‐5/PS@Cu cell exhibits nearly 2000 stable deposition behaviors with an average CE of 99.2%. In sharp contrast, Zn//CMK‐5@Cu cells show reversible electroplating/stripping within 800 cycles, while Zn//Cu cells only last for less than 150 cycles, due to the dendrite formation and parasitic reactions. This proves the high reversibility of Zn deposition on CMK‐5/PS decorated substrate from the unique structures.

We further assembled symmetric cells to evaluate the reversibility of the modified Zn anode. As shown in Figure 3g, the cyclic stability of the cell with CMK‐5/PS@Zn anode can reach over 8500 h at 1 mA cm^−2^‐0.5 mAh cm^−2^ with no visible voltage fluctuation, superior to others reported (Table S2). The CMK‐5@Zn could maintain reversibility for 630 h, while the cell with the bare Zn anode can only last for 300 h with significant voltage fluctuation. The CMK‐5/PS@Zn anode exhibits an excellent cycling durability of ∼4500 h at 0.5 mA cm^−2^‐0.5 mAh cm^−2^, which is much longer than that of the CMK‐5@Zn anode (∼1500 h) and bare Zn anode (∼480 h) (Figure S23a). Moreover, the cell with CMK‐5/PS@Zn anode can still maintain close to 1500 h stability operation at an increased current density and reversible capacity (4 mA cm^−2^ and 2 mAh cm^−2^). On the other hand, both the cells with CMK‐5@Zn and bare Zn anodes exhibit an increasing trend in the hysteretic voltage during the cycling operation, with the sudden increase in voltage after 650 h (CMK‐5@Zn) and 150 h (bare Zn), respectively (Figure S23b). As the current density and capacitance continue to increase (5 mA cm^−2^ and 5 mAh cm^−2^), the CMK‐5/PS@Zn anode undoubtedly exhibits much longer cyclic stability than the CMK‐5@Zn and bare Zn electrodes (Figure S23c). It is worth mentioning that the CMK‐5/PS@Zn could maintain relatively stable cycling capability at elevated capacities from 2 to 10 mAh cm^−2^ when the CMK‐5@Zn and bare Zn electrode display much higher voltage hysteresis and short cycle lifespans (Figure S24). Furthermore, chronoamperometry (CA) was performed on the cells to show the different diffusion patterns of CMK‐5/PS@Zn, CMK‐5@Zn, and bare Zn anodes (Figure 3h). The results indicate that both CMK‐5/PS@Zn and CMK‐5@Zn are 3D diffusion, while bare Zn displays 2D patterns. It can be concluded that the high reversibility of CMK‐5/PS@Zn anode should be attributed to the unique properties of the CMK‐5/PS interface with Junas structure, which exhibits the combination of functional organic decoration to enhance ionic transport as well as directional confined Zn deposition and multi‐channel carbon substrate to provide electronic conductivity as well as fast Zn^2+^ transport through hierarchical porous structures. The design is highly beneficial for Zn anode as the enhancement of ionic and electronic conductivities ensures smooth Zn deposition to suppress dendrite growth and fast kinetics (Figure S25; Tables S3 and S4), while the confinement of the deposited Zn in the porous structure reduces the direct contact to the electrolyte, leading to a significant reduction in parasitic reactions. Therefore, as a result, CMK‐5/PS@Zn symmetric cells display the best rate capability with the current density increasing from 0.5 to 10 mA cm^−2^ and excellent capacity retention when returning to 0.5 mA cm^−2^, compared to the counterparts CMK‐5@Zn and bare Zn electrodes (Figure 3i; Figures S26–S28). Meanwhile, the SEM image and electrochemical impedance measurements of the symmetric cell after cycling at 1 mA cm^−2^‐1 mAh cm^−2^ further confirm the above results (Figures S29 and S30).

### 2.4 Postmortem Characterizations of Electrodes after Cycling

**FIGURE 4:**
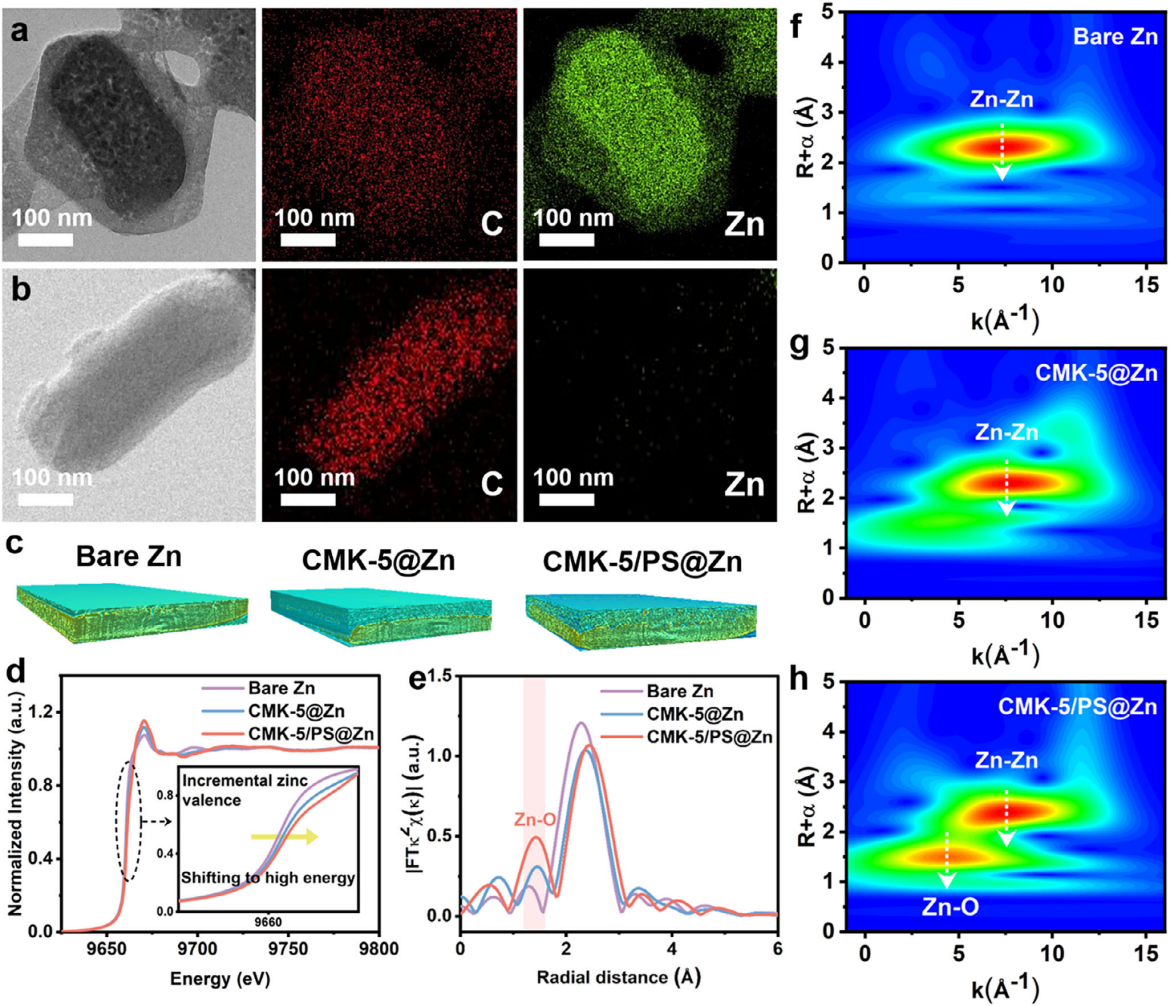
Postmortem characterizations of electrodes after cycling. TEM image and element distribution map of CMK‐5/PS@Zn anode after (a) Zn plating and (b) Zn stripping; (c) 3D rendering images with MCT of Zn plating/stripping interface in a Zn//Zn symmetric cell after cycling at 1 mA cm^−2^, 1 mAh cm^−2^, with the Zn in yellow and the secondary species other than Zn in green; The Synchrotron X‐ray absorbing near‐edge structures of bare Zn, CMK‐5@Zn and CMK‐5/PS@Zn for (d) K‐edge and (e) R‐space; X‐ray absorption near edge structure of (f) bare Zn, (g) CMK‐5@Zn and (h) CMK‐5/PS@Zn for wavelet transform.

In order to comprehensively verify the regulatory effects of CMK‐5/PS interfacial layer during Zn plating and stripping, we performed a series of post‐mortem characterizations on the anodes after cycling. Atomic force microscopy (AFM) was employed to reveal the surface morphology change after cycling compared to their pristine states (Figures S30 and S31). The surface of bare Zn displays a significant change before and after cycling, with obvious height fluctuations during plating and stripping. The surface morphology does not return to the original state on the bare Zn after stripping, indicating a low Coulombic Efficiency with significant parasitic reactions. CMK‐5@Zn and CMK‐5/PS@Zn, on the other hand, display relatively flat and uniform surfaces after plating, which return to the original height after stripping. Especially, CMK‐5/PS@Zn exhibits the lowest height variations (consistent with the 3D laser confocal microscopy in Figure S33), indicating that the Zn deposition patterns on CMK‐5/PS@Zn may be different from those of the other counterparts. To verify this, we conducted TEM mapping on the CMK‐5/PS after plating and stripping (Figure 4a,b). Intriguingly, Zn is primarily deposited in the inner pores of the carbon matrix, owing to the high Zn^2+^ affinity and low energy required for Zn deposition for the organic‐decorated surface in the inner pores. The hierarchical porous structure also facilitates the process, allowing the Zn^2+^ to diffuse through the pores more efficiently. The deposited Zn can be completely removed during the subsequent charging, referring to a high reversibility of Zn plating/stripping. In addition, we employed the synchrotron Micro‐Computed Tomography (MCT) to observe the topographical composition and morphology of the anodes after Zn plating/stripping cycles (Figure 4c; Figure S34). As shown in Figure 4c, all three anodes exhibit a two‐layer structure (Zn and non‐Zn components), but with markedly different features. For bare Zn, the thin surface layer corresponds to an SEI with lower X‐ray attenuation than metallic Zn, and its relatively large thickness reflects severe side reactions. In contrast, CMK‐5@Zn shows a much thicker upper layer derived from the CMK‐5 coating, with a sharp interface indicating that Zn deposits mainly on the underlying Zn metal rather than on the CMK‐5 due to their distinct attenuation contrasts. For CMK‐5/PS@Zn, however, the boundary between the two layers disappears. A homogeneous mixed‐color region emerges because of overlapping attenuation signals of Zn and the CMK‐5/PS coating, directly demonstrating that Zn deposits within the hierarchical porous framework. The smooth color transition further suggests preferential Zn‐ion migration into the porous layer and confined nucleation, rather than uncontrolled dendritic growth [56]. The results are consistent with the TEM results in Figure 4a,b, verifying a directional regulation effect on Zn deposition in the carbon matrix.

We also performed Synchrotron X‐ray absorption spectroscopy (XAS) on the anodes after Zn stripping/plating cycles (Figure 4d,e; Figure S35). The Synchrotron XANES results in Figure 4d show a shift in the absorption edge of Zn to higher energy, compared to the counterparts, which indicates an increase in Zn valence of the deposited Zn in the CMK‐5/PS layer [57]. This should be assigned to the interactions between the confined Zn and sulfonic anions in the porous structure of the carbon matrix. This is further verified by the extended X‐ray absorption fine structure (EXAFS) results and 2D wavelet transform results in Figure 4f–h that an additional Zn‐O bond appears in the CMK‐5/PS@Zn [4, 58]. These results clearly demonstrate the directional deposition of Zn in the Janus CMK‐5/PS structure, which is consistent with the microscopic results in Figure 4a–c.

### 2.5 In Situ Mechanistic Study of Interfacial Layers

**FIGURE 5:**
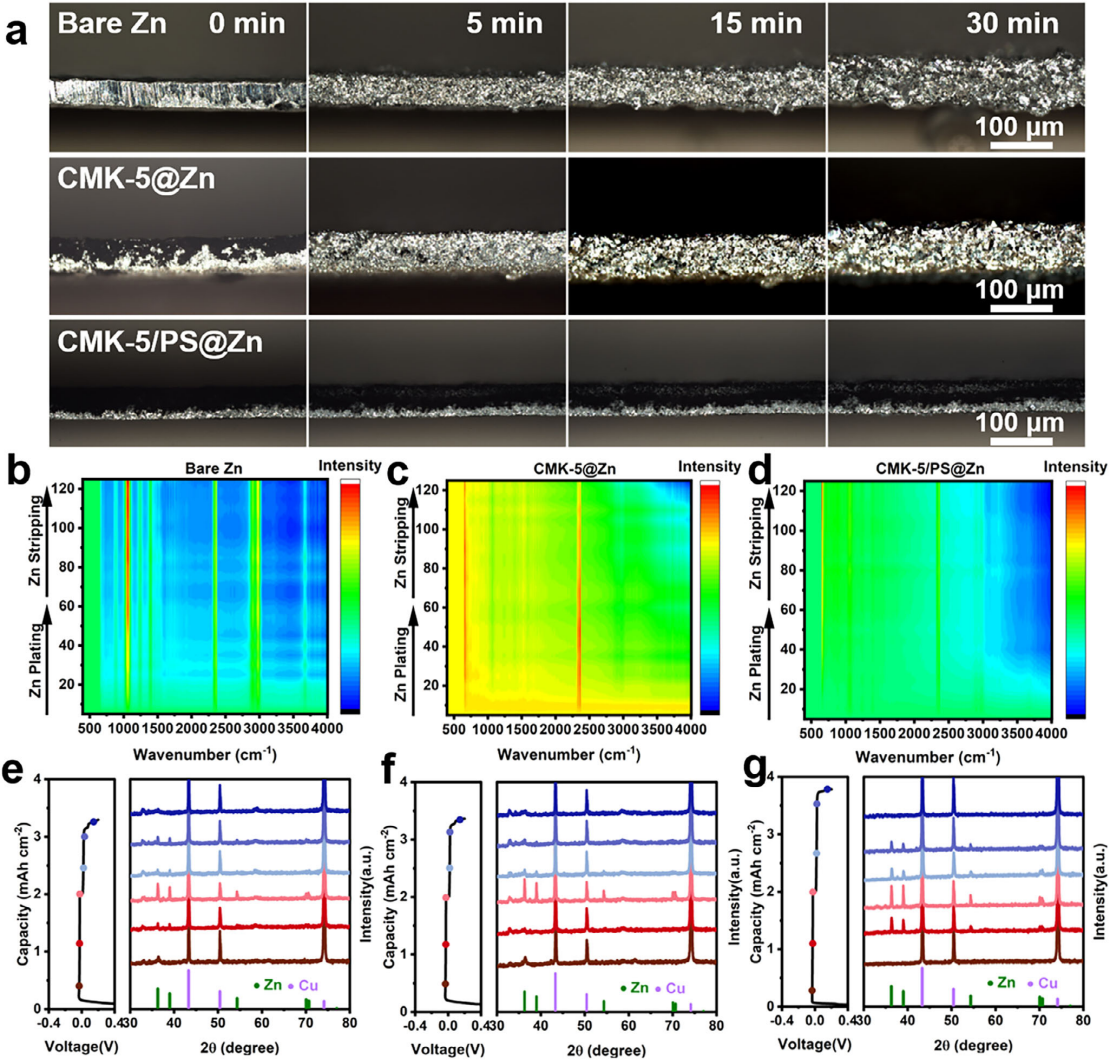
In situ mechanistic study of interfacial layers. In situ optical microscopy images of (a) bare Zn, CMK‐5@Zn and CMK‐5/PS@Zn symmetric batteries at 5 mA cm^−2^; Synchrotron radiation infrared spectrum analysis of (b) bare Zn, (c) CMK‐5@Zn and (d) CMK‐5/PS@Zn symmetric battery was performed by using In situ mode; XRD pattern of (e) bare Cu, (f) CMK‐5@Cu and (g) CMK‐5/PS@Cu electrode during the 10th Zn plating/stripping cycle.

We further performed in situ optical microscopy on the CMK‐5/PS@Zn, CMK‐5@Zn and Zn anodes to visualize the Zn deposition process (Figure S36). As shown in Figure 5a, small prominences appear on the bare Zn anode electrode surface just after 5 min of electroplating, and continue to grow into dendrites and bubbles after 30 min, referring to significant parasitic reactions. The morphology change of CMK‐5@Zn anode appears much better than the bare Zn anode, but the growth of Zn shows irregular shapes with visible protrusions on the surface after 30 min. This is mainly because the carbon substrate on the Zn surface can facilitate relatively smooth Zn deposition. However, the non‐directional growth of Zn may eventually lead to dendrite growth after longer exposure. The CMK‐5/PS@Zn anode, on the other hand, maintains a relatively smooth surface throughout the process without obvious dendrites. It is worth mentioning that the thickness of the anode does not increase significantly, and the growth of Zn appears to be inside the carbon matrix with the obvious change of color. This is consistent with the surface morphology changes in symmetric cells after deposition of different capacities (Figure S37). This indicates that the Janus nature of CMK‐5/PS interfacial layer can enable directional growth of Zn inside the pores, which can significantly reduce the parasitic reactions and dendrite growth.

In situ synchro‐FTIR tests were also performed to monitor the dynamic change in chemical species at the electrode/electrolyte interface during the Zn deposition/dissolution process. As shown in Figure 5b–d, the tensile vibration strength of O‐H at 3200 ∼ 3600 cm^−1^ gradually decreases, indicating the reduction of active water and the change of solvation structure during zinc plating [59], while the characteristic peak intensity at 1000–1100 cm^−1^ is reduced, indicating the amount of adjacent SO_4_^2−^ anion is reduced. The results show that SO_4_^2−^ anions are expelled from the surface due to the desolvation of Zn^2+^ during Zn plating, which is consistent with the variation of free water. The process reverses during the stripping process. Similar phenomenon occurs with CMK‐5@Zn//CMK‐5@Zn and Zn//Zn batteries, however, with higher concentration, indicating CMK‐5/PS facilitates the rapid removal of the fee water and SO_4_^2−^, enabling uniform distribution and nucleation of Zn^2+^.

In addition, we further performed X‐ray diffraction (XRD) on Zn//Cu cells to dynamically monitor the Zn deposition (Figure 5e–g). The results show that the Zn metal signal on CMK‐5/PS appears and increases with the progress of the deposition process. During the stripping process, the intensity of Zn decreases until it fully disappears. A similar trend is found on the CMK‐5 electrode, with slightly lower intensity and reversible capacity. In contrast, the intensity and reversible capacity of the bare Cu electrode have been significantly reduced, while the peaks related to parasitic reactions can be spotted during the discharge and charge process, due to the lower round‐trip efficiency. It can be concluded that the CMK‐5/PS interfacial layer on Zn anodes contributes significantly to regulating the growth and deposition of Zn, owing to the unique intrinsic properties. The multi‐channel carbon matrix of CMK‐5 facilitates the electronic conductivity and allows Zn^2+^ penetration through micropores to facilitate the Zn deposition. Organic‐sulfonic‐ion‐modified surface in the Janus structure can promote the ionic transport of Zn^2+^ while ensuring the directional deposition exclusively within the pores. This leads to the confinement of the deposited Zn inside the pores, resulting in shortened exposure of the unprotected Zn to the electrolyte and suppressed parasitic reactions. These findings highlight the importance of the interfacial design for Zn anodes, which has important implications for high‐performance Zn‐based batteries.

### 2.6 The Evaluation of Full Cell Performance

**FIGURE 6:**
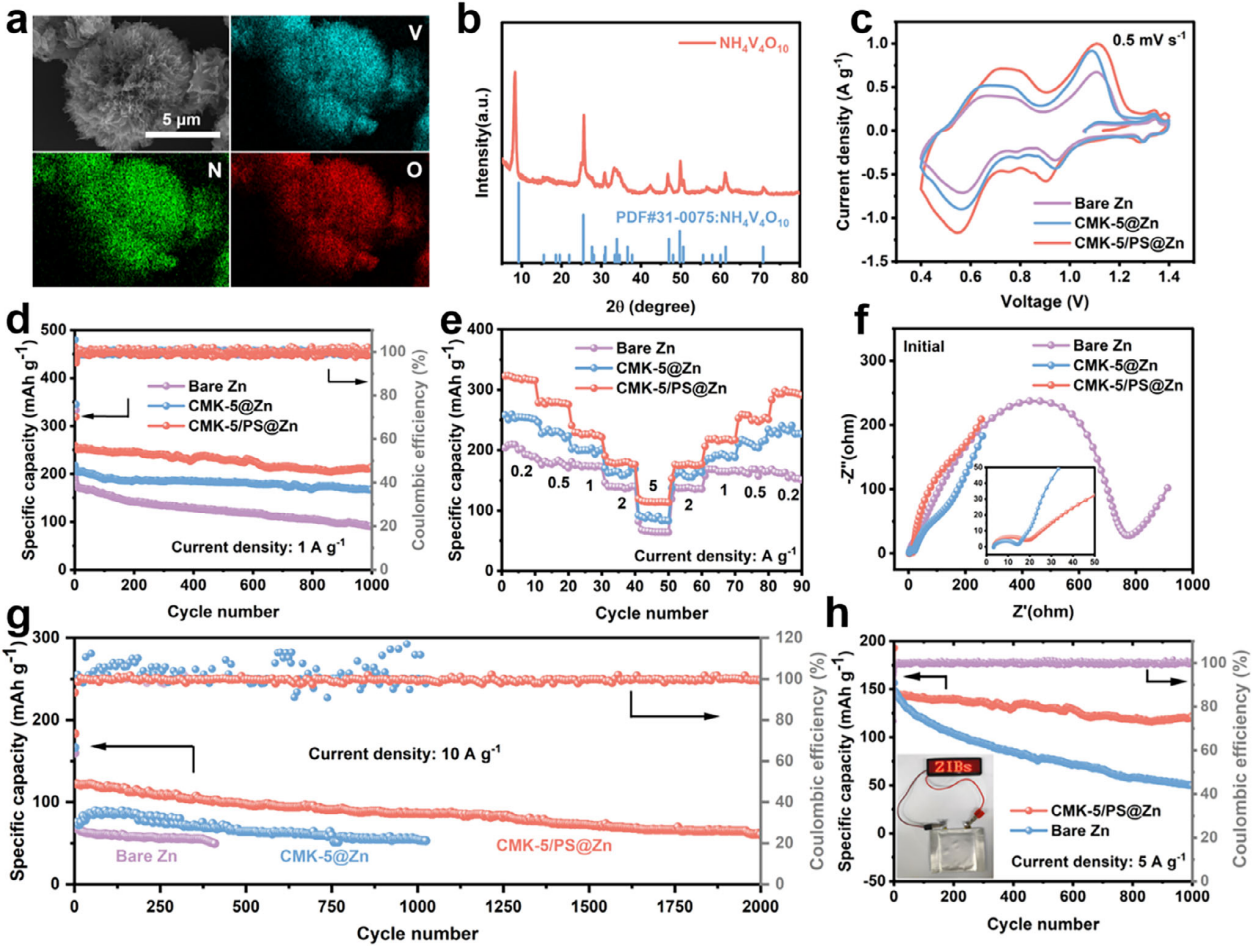
The evaluation of full cell performance. (a) SEM and elemental diagram; (b) XRD pattern of NH_4_V_4_O_10_ powder; (c) At a scanning rate of 0.5 mV s^−1^, CV curves of Zn||NH_4_V_4_O_10_, CMK‐5@Zn||NH_4_V_4_O_10_ and CMK‐5/PS@Zn||NH_4_V_4_O_10_ full cell; Long‐term cycling performance at current densities of (d) 1 A g^−1^, (g) 10 A g^−1^; (e) Rate capacities of Zn||NH_4_V_4_O_10_, CMK‐5@Zn||NH_4_V_4_O_10_ and CMK‐5/PS@Zn||NH_4_V_4_O_10_ full batteries ranging from 0.2 to 5A g^−1^ in 2 M ZnSO_4_ electrolyte; (f) EIS curve for the full cell; (h) Zn||NH_4_V_4_O_10_, CMK‐5/PS@Zn||NH_4_V_4_O_10_ pouch cell long cycle performance at 5A g^−1^ and Photo of CMK‐5/PS@Zn||NH_4_V_4_O_10_ full cell lit LED panel.

We further assembled full cells with the modified anode to evaluate the performance in the AZIB, by pairing them with synthesized NH_4_V_4_O_10_ as the cathode, due to its layered structure, multivalent redox properties, and excellent structural stability [60, 61, 62]. We performed SEM and XRD to confirm the successful synthesis of NH_4_V_4_O_10,_ which displays a morphology of a flower shape composed of nanosheets (Figure 6a,b). As shown in the CV results in Figure 6c, all CV curves exhibit two pairs of redox peaks at a scanning rate of 0.5 mV s^−1^, which refers to the insertion/extraction of Zn^2^^+^ ions within the layered structure and the multivalent redox reactions of vanadium [63, 64]. Slightly higher peak intensities can be observed from the CMK‐5/PS@Zn//NH_4_V_4_O_10_ battery, showing relatively higher zinc storage capacity than the counterparts, which is consistent with the discharge and charge performance of the Zn‐ion batteries. Furthermore, the introduction of the CMK‐5/PS interfacial layer significantly improves the stability of the battery, exhibiting excellent rate capability and cycling performance (Figure 6d; Figure S38). As the current density increases from 0.2 A g^−1^ to 5 A g^−1^ and back to 0.2 A g^−1^, the capacity of CMK‐5/PS@Zn//NH_4_V_4_O_10_ cell is consistently higher than that of CMK‐5/PS@Zn//NH_4_V_4_O_10_ and Zn//NH_4_V_4_O_10_ cells, corresponding to outstanding rate capabilities from the Janus carbon matrix (Figure 6e). Meanwhile at 1 A g^−1^, CMK‐5/PS@Zn//NH_4_V_4_O_10_ battery can maintain a high reversible capacity of 213.8 mAh g^−1^ after 1000 cycles, with a high capacity retention rate of 98.2%. In contrast, CMK‐5@Zn//NH_4_V_4_O_10_ and Zn//NH_4_V_4_O_10_ batteries display a low reversible capacity of 167.43 and 91.03 mAh g^−1^, respectively. We applied the electrochemical impedance (EIS) on these batteries (Figure 6f; Figure S39), which shows a significant reduction in charge transfer resistance (R_ct_) of the CMK‐5/PS@Zn//NH_4_V_4_O_10_ battery than the CMK‐5/PS@Zn//NH_4_V_4_O_10_ battery and Zn//NH_4_V_4_O_10_ battery, even after 50 cycles. This result indicates that the modified CMK‐5/PS interfacial layer can enhance and maintain charge transfer behavior during cycling. The morphology of the negative electrodes remains relatively smooth on the CMK‐5/PS@Zn after 50 cycles (Figure S40). CMK‐5@Zn shows slight protrusion growth, while a large number of dendrite is observed on the surface of the bare Zn electrode. This result is consistent with the symmetry cells in Figure 3. We further evaluated the performance of the full cells by increasing the current density to 5 and 10A g^−1^, revealing that CMK‐5/PS@Zn//NH_4_V_4_O_10_ batteries still maintain the highest capacity and stability than the ones with CMK‐5@Zn anode and bare Zn anode (Figure S41; Figure 6g). Figure S42 shows the self‐discharge performance when the cells were charged to 1.4 V and held at open circuit for 24 h. The bare Zn||NH_4_V_4_O_10_ full cell retained only 66.68% of its initial capacity after resting for 24 h, while the CMK‐5@Zn∥NH_4_V_4_O_10_ cell maintained 73.96%. In contrast, the CMK‐5/PS@Zn∥NH_4_V_4_O_10_ cell preserved as much as 86.99% of its initial capacity after the 24 h rest. This superior performance can be attributed to the CMK‐5/PS layer, which provides a highly stable interfacial environment and effectively suppresses parasitic reactions. It is worth mentioning that the pouch‐type cell of CMK‐5/PS@Zn//NH_4_V_4_O_10_ with enlarged electrode surface areas (25 cm^2^) exhibits excellent cycling capability to over 1000 cycles, and subsequently has the capability to light up an LED panel (Figure 6h), demonstrating that the introduction of the CMK‐5/PS interfacial layer can significantly improve the stability of Zn anode.

## 3 Conclusion

In summary, the Janus hierarchical CMK‐5 with a hierarchical multichannel design was developed through an organic modification strategy, which was applied to construct the functional interfacial layer on Zn metal anodes to suppress dendrite growth and parasitic reactions. The designed hierarchical structure presented a unique porous structure and a high specific surface area, which effectively facilitated the migration and deposition of Zn^2+^ ions. Meanwhile, the controlled immobilization of sulfonic anions in the pores reduced the energy barrier for Zn deposition and facilitated the directional Zn growth, demonstrated by DFT calculations. Benefiting from the unique properties and intrinsic high electronic/ionic conductivity from the anion‐modified carbon matrix, the interfacial layer exhibited high zincophilicity to allow fast Zn plating/stripping exclusively in the pores, effectively reducing the exposure time, which resulted in uniform Zn deposition with suppressed dendrite growth and parasitic reactions. Therefore, the resulting anode displayed a small nucleation overpotential, high reversibility, and excellent Coulomb efficiency up to 99.2% in an asymmetry Zn//Cu cell. The CMK‐5/PS@Zn symmetric battery exhibited an ultra‐long cycle stability of 8500 h at 1 mA cm^−2^‐0.5 mAh cm^−2^. Notably, the assembled CMK‐5/PS@Zn//NH_4_V_4_O_10_ battery showed a high specific capacity of 213.8 mAh g^−1^ at 1 A g^−1^ after 1000 cycles, providing a practical interface modification strategy to develop dendrite‐free metal anodes for aqueous Zn‐ion batteries.

## Author Contributions

H.L., Y.Z., and J.Z. supervised the research. L.G. and J.Z. designed the research. H.C. performed COMSOL simulations. Y.Z. conducted DFT calculations. L.G. performed the synthesis, characterizations, and electrochemical tests. D.L. L.Z., and Y.L. contributed to the synchrotron tests and analysis with the help of J.V. and B.J., L.G., Y.Z., and J.Z. analyzed the data. L.G., C.Q., C.Y., B.Y., H.G., J.Z., G.W., and H.L. contributed to the discussion and preparation of the manuscript.

## Conflicts of Interest

The authors declare no conflicts of interest.

## Supporting information




**Supporting File 1**: advs74054‐sup‐0001‐SuppMat.docx.

**Supporting File 2**: advs74054‐sup‐0002‐MovieS1.mp4.

**Supporting File 3**: advs74054‐sup‐0003‐MovieS2.mp4.

**Supporting File 4**: advs74054‐sup‐0004‐MovieS3.mp4.

**Supporting File 5**: advs74054‐sup‐0005‐MovieS4.mp4.

## Data Availability

Data sharing is not applicable to this article as no new data were created or analyzed in this study.
