# Supplementary material for: Directional Regulation of Zinc Deposition Through Constructing Hierarchical Janus Carbon Matrix with Organic‐Decorated Multi‐Channels
Source: Adv Sci (Weinh). 2026 Jan 27;13(19):e22959. doi: 10.1002/advs.202522959 (PMC13045425; doi:10.1002/advs.202522959)
Supplement: Supplementary file 1 — Supporting File 1: advs74054‐sup‐0001‐SuppMat.docx. [file ADVS-13-e22959-s003.docx]

**Directional regulation of zinc deposition through constructing hierarchical Janus carbon matrix with organic-decorated multi-channels**

***Li Gao^1,2^, Hongli Chen^3^,*** ***Dongfang Li^2^, Chen Qian^1^, Chao Yuan^1^, Bin Yu^1^, Jitraporn Vongsvivut^4^, Bernt Johannessen^4^, Lingfei Zhao^2^, Yaojie Lei^2^, Hong Gao^1^, Yufei Zhao*^2^, Jinqiang Zhang*^2^, Guoxiu Wang*^2^, Hao Liu*^2^***

^1^ Joint International Laboratory on Environmental and Energy Frontier Materials, School of Environmental and Chemical Engineering, Shanghai University, China.

^2^ Centre for Clean Energy Technology, University of Technology Sydney, Broadway, Sydney, NSW 2007, Australia.

^3^ State Key Laboratory of Chemical Engineering, East China University of Science and Technology, Shanghai 200237, P. R. China.

^4^ Australian Synchrotron, ANSTO, 800 Blackburn Road, Clayton, VIC, 3168, Australia

* Corresponding Authors. Emails: Yufei.Zhao@uts.edu.au; Jinqiang.Zhang@uts.edu.au; Guoxiu.Wang@uts.edu.au; Hao.Liu@uts.edu.au;

**Experimental section**

**1. Materials**

Zinc foil (99.999%, 0.1 mm) and titanium foil (99.999%, 0.02 mm) were procured from the High Purity Metals Research Institute. Acetylene black and polyvinylidene fluoride (PVDF) were sourced from Guangdong Chuglight New Energy Technology Co., Ltd. Zinc sulfate heptahydrate (ZnSO_4_·7H_2_O) (≥99.5%), 1,3-propanesultone (99%), and ammonium metavanadate (NH_4_VO_3_) were purchased from Macklin. Acetonitrile (AR), PEO-PPO-PEO (P123), Tetraethyl Orthosilicate (TEOS), Oxalic Acid (C_2_H_2_O_4_), furfuryl alcohol and Trimethylbenzene were acquired from China National Pharmaceutical Group Corporation. N-methyl-2- pyrrolidone (NMP) was obtained from GENERALREAGENT. Glass microfber flter (GF/D) was procured from Whatman. Deionized water was used for preparing aqueous electrolytes. The high-purity N_2_ (Shanghai Yujiali Special Gas Co., Ltd. 99.999%) was used directly.

**2. Material Synthesis**

***Preparation of CMK-5/PS@Zn electrode***

**Preparation of SBA-15**: Dissolve 0.4g PEO-PPO-PEO (P123) in 105 ml water and slowly stir magnetically until completely dissolved. Add another 20 ml 37% concentrated hydrochloric acid drops to the above solution, then transfer the solution to a 40℃ water bath and continue to stir magnetically for 20 minutes. Then add 9.1 ml tetraethyl orthosilicate (TEOS) drop by drop at 1000 rpm, and add it within 5 minutes, continue magnetic stirring for 10 minutes. Subsequently, the speed was changed to 700 rpm, and after stirring for 4h, the solution was transferred to a 200 ml reactor and reacted at 100°C for 24h After the reaction, it is naturally cooled to room temperature, cleaned with deionized water several times, and dried in vacuum overnight. The product was calcined in Muffle furnace at 550℃ for 6h, and the heating rate was 1℃/min.

**Synthesis of CMK-5**: CMK-5 material was prepared using SBA-15 as hard template.^[1]^ Put 0.037g oxalic acid (C_2_H_2_O_4_) into the sample bottle, remove 4 ml furfuryl alcohol and add it, after ultrasonic dissolution, add 6 ml trimethylbenzene and continue ultrasonic dissolution. Weigh 0.4g SBA-15 in sample bottle, add 900 µl oxalic acid solution and mix thoroughly. The mixed sample was sealed at 50℃ for 24h, then reacted at 90℃ for 24h, and then reacted at 90℃ unsealed for 30 minutes. The obtained sample was calcined in a nitrogen atmosphere at 850℃ for 4h. Finally, SiO_2_ is etched under the product alkali solution to obtain the final product, namely CMK-5.

**Synthesis of CMK-5/PS organic layer composite materials**: Dissolve 100 mg of unetched CMK-5 in 15 ml of acetonitrile, add 5 mmol of 1,3-propanesulfonate magnetically stir until dissolved, and then seal the solution at 75℃ for 24h. After cooling, the solution was poured into ether to precipitate, cleaned with ethanol several times, and then vacuum dried at room temperature. SiO_2_ is then etched in the alkali solution to obtain the final product, CMK-5/PS.

**Preparation of CMK-5/PS@Zn and CMK-5@Zn electrode**: Commercial zinc foil 0.1mm thick was cut into zinc sheets with a diameter of 12 mm, and then the zinc foil was continuously cleaned with alcohol ultrasonic for 10min. To prepare the electrode, CMK-5/PS powder, carbon black and polyvinylidene fluoride (PVDF) were fully mixed in N-methyl-2-pyrrolidone (NMP) solvent at a mass ratio of 8:1:1, and the slurry was obtained by magnetic stirring. Drops of the mixture are then coated on Zn foil and dried in a vacuum oven at 80°C for 12 h. CMK-5@Zn electrode was prepared by the same method.

***Preparation of NH_4_V_4_O_10_ cathode electrode***

NH_4_V_4_O_10_ powder was synthesized by a simple hydrothermal reaction method.^[2]^ 1.17g ammonium metavanadate (NH_4_VO_3_) was dissolved in 35ml water and stirred at 80℃ to form a light yellow solution. Then, 1.89g oxalic acid crystal (H_2_C_2_O_4_·_2_H_2_O) was added to the solution and magnetic stirring was continued until the solution became dark green. The solution was transferred to a 50ml reactor, kept at 140℃ for 48h, cooled to room temperature, washed several times with deionized water, and dried overnight under vacuum at 80℃ to obtain dark green NH_4_V_4_O_10_ powder. When preparing the electrode, NH_4_V_4_O_10_, carbon black and polyvinylidene fluoride (PVDF) were fully mixed in N-methyl-2-pyrodanone (NMP) solvent at a mass ratio of 7:2:1, and the slurry was obtained by magnetic stirring. The mixture is then pasted on a Ti foil and dried in a vacuum oven at 80°C for 12 h. The load mass of the positive electrode material is about 1 ~ 2 mg cm^-2^.

**3. Material Characteristic**

The microstructure and element distribution were studied by scanning electron microscopy (field emission SEM, 7500F, JEOL LTD), energy dispersive spectroscopy (EDS) and field emission transmission electron microscopy (TEM, JEM-2100F). The electrode structures at 40 kV and 30 mA were analyzed using 18KW X-ray diffractometer (XRD) under Cu target radiation. The specific surface area (BET) and pore size distribution of the materials were measured by nitrogen adsorption desorption apparatus. The functional groups were characterized by Fourier transform infrared spectroscopy (FTIR) of Thermo Scientific Nicolet iS20 FTIR spectrometer. Qualitative test of materials with Raman spectrometer. The thermogravimetric analyzer (TG209F1) tests the sulfur content of materials. The surface chemical composition was determined by X-ray photoelectron spectroscopy (XPS). The contact Angle measurement was performed on an OCA 25 contact Angle meter with an electrolyte dosage of 1μL. *In*-situ optical images were obtained on a high-performance optical microscopy system (Smart Zoom5) using a self-made in situ optical electrochemical cell in a 2 M ZnSO_4_ electrolyte with the current density of 5 mA cm^-2^ in a 100 μm scale. The electrode height was observed under atomic force microscope. Atomic force microscopy (AFM) images were obtained using a Korean Park Systems XE7 operating in tapping mode. The synchrotron MCT (Micro-Computed Tomography) experiment was carried out at Australia's Nuclear Science and Technology Organisation (ANSTO) with a mono beam detector of 20 keV, magnification of 20, and effective pixel size 0.325µm (a white beam detector of 39 keV, a magnification of 9, and an effective pixel size of 0.65 µm). Sulfur K-edge XANES measurements were performed at the MEX-2 beamline (Medium Energy XAS) of the Australian Synchrotron (ANSTO). A monochromator was employed to select the incident X-ray energy in the range of 2.45-2.55 keV. Spectra were collected in transmission/fluorescence mode depending on the sample concentration. The energy step was set to 0.2 eV in the near-edge region (2465-2490 eV) to obtain detailed edge features. All spectra were calibrated against reference compounds (Na_2_S_2_O_3_ for S^6+^ at 2482.0 eV, or ZnS for S^2-^ at 2470.0 eV) and normalized/background-subtracted using ATHENA software. Zn K-edge XAS measurements were performed at the XAS beamline of the Australian Synchrotron (ANSTO). In situ synchrotronbased FTIR experiments were performed on the Infra-red Microspectroscopy (IRM) beamline at ANSTO-Australian Synchrotron (Clayton, Victoria), using a Hyperion 3000 FTIR microscope coupled to a Vertex 70/70 v FTIR spectrometer (Bruker Optik GmbH, Ettlingen, Germany), equipped with a customised Si crystal (250 µm). The test was performed on a Zn//Zn symmetrical-electrode setup using a three-electrode at a charge/discharge current density of 10 mA cm^–2^ for 10 min.

**4. Electrochemical Measurements**

Using glass fiber filter (GF/D, Whatman) as separator, the CR-2032 coin cell was assembled and electrochemical test was carried out in air atmosphere. Zn//Zn, CMK-5@Zn//CMK-5@Zn and CMK-5/PS@Zn//CMK-5/PS@Zn symmetric cells, Zn//Cu, CMK-5@Zn//Cu, CMK-5/PS@Zn//Cu asymmetric cells and Zn//NH_4_V_4_O_10_, CMK-5@Zn//NH_4_V_4_O_10_ and CMK-5/PS@Zn//NH_4_V_4_O_10_ full cell were prepared as electrolytic solution with 2 M ZnSO_4_ aqueous solution. GCD cycle tests were carried out on the Neware battery test system. Cyclic voltammetry (CV), linear sweep voltammetry (LSV), Tafel curve and electrochemical impedance (EIS) were performed at the CHI 760E Electrochemical Workstation (CHI 760E, Shanghai Chenhua). Briefly, the CE was evaluated in asymmetric Zn//Cu coin cells, where a Cu foil served as the working electrode and a Zn foil was used as both the counter and reference electrode. The electrolyte was 2 M ZnSO_4_ aqueous solution, and a glass fiber membrane (Whatman GF/D) was used as the separator. In each cycle, Zn was first plated onto the Cu substrate with a fixed areal capacity of 1 mAh cm^-2^ at a current density of 1 mA cm^-2^, followed by stripping at the same current density to a cutoff voltage of 0.8 V (vs. Zn^2+^/Zn). The Coulombic efficiency was calculated as CE = Q_strip_/Q_plate_ × 100%, where Q_plate_ and Q_strip_ refer to the plating and stripping capacities, respectively. The CE values reported in this work were averaged over cycles 50-100 to exclude the initial activation process.

**5. DFT**

All density functional theory (DFT) calculations were implemented by the Vienna Ab initio Simulation Package (VASP).^[3]^ The electron exchange and correlation energies were handled using the Perdew-Burke-Ernzerhof (PBE) functionals.^[4]^ The projector augmented wave (PAW) potentials was used to describe the interactions between the cores and valence electrons.^[5]^ The expansion of the Kohn-Sham valence states was carried out with a 400 eV plane-wave cutoff energy. For Brillouin zone integration, 3 × 3 × 3 Γ-centered Monkhorst-Pack grids were performed. The Zn (002) surface unit cells were four layers thick, and the bottom two layers were fixed to the bulk position of Zn. The convergence criterion of structure optimization for energy and force were set as 10^-4^ eV and 0.03 eV Å^-1^, respectively. Constrained ab initio molecular dynamics (cAIMD) simulations with a SG sampling approach as implemented in VASP (SG-AIMD) are performed to evaluate the kinetic process of desolvation of zinc ions with imidazole.^[7,8]^ During SG-AIMD, the bottom two layers of the Zn metal substrate were fixed. Due to the complexity of the SEI composition on the zinc anodes, we simplified the models by constructing carbon layers with the same thickness on the zinc metal surface.

**6. COMSOL simulations.**

The current density and the Zn-ion concentration were simulated by the finite element method (FEM) using COMSOL Multiphysics 6.2 software with the “Tertiary Current Distribution” module. The size of the entire two-dimensional model for electric field distribution analysis was set to 40×30 μm. In this model, all the parameters of the interdigital electrode structure were consistent with those employed in the experiments.

The Zn^2+^ transfers by the concentration diffusion in model follow the Fick's law as shown in equation (1) and (2):

$N_{i}=J_{i}={-D}_{i}\nabla c_{i}$ (1)

$\frac{\partial c_{i}}{\partial t}+\nabla J_{i}=R_{i,tot}$ (2)

where $J_{i}$ is the ion flux, $D_{i}$ is the diffusion coefficient of electrolytes ($D_{Zn}=2\times{10}^{-9} m^{2}s^{-1}$), $c_{i}$ is the ion concentration of electrolytes ($c_{i}=2.0 M$), $\nabla c_{i}$ is concentration gradient.

The relation between the diffusion coefficient and electric mobility follows the Nernst-Einstein relation as shown in equation (3):

$N_{i}={-D}_{i}\nabla c_{i}-z_{i}u_{m,i}Fc_{i}{\nabla\emptyset}_{l}+uc_{i}=J_{i}+uc_{i}$ (3)

where $z_{i}$ is the transfer number ($z_{Zn}=2$), $u_{m,i}$ is the electric mobility coefficient, $F$ is the Faraday constant (96485 C mol^-1^), 𝜙 is the electrolyte potential.

**Finite element simulations.**

In order to simulate the zinc deposition behaviors, a two-dimensional mechano-electrochemical model with a size of 1.6×1.2 μm was established. The two phases and three components in this system were distinguished by a non-conserved order parameter ξ (electrolyte, ξ=0; Zn metal anode, ξ=1) and a concentration set ci (i=Zn, Zn^2+^, and SO_4_^2−^, respectively). The local electrostatic potential was denoted as ϕ_i_ (i=Zn and e, representing zinc metal electrode and electrolyte, respectively) and the displacement field was represented by u. The total free energy of this system was given by

$F=\int_{V} \left[ f_{grad}\left( \xi\right)+f_{ch}\left( \xi,c_{i} \right)+f_{elec}\left( \xi,c_{i},\phi_{i} \right) \right]dV$ (4)

where $f_{grad}$, $f_{ch}$, and$f_{elec}$ represent the local energy density from the gradient, chemical, and electrostatic contribution, respectively. More specifically, the gradient energy density $f_{grad}$ was expressed by

$f_{grad}\left( \xi\right)=\frac{1}{2}\kappa_{0}[1+\delta cos(\omega\theta)]\nabla^{2}\xi$ (5)

where $\kappa_{0}$ is the gradient energy coefficient, $\delta$ is the strength of anisotropy, $\omega$ is the mode of the anisotropy, and $\theta$ is the angle between the normal vector of the interface and the reference axis. The chemical and electrostatic energy density can be written as

$f_{ch}\left( \xi,c_{i} \right)=g\left( \xi\right)+RT\left( c_{{Zn}^{2+}}ln\left( \frac{c_{{Zn}^{2+}}}{c_{0}} \right)+c_{anion}ln\left( \frac{c_{anion}}{c_{0}} \right) \right)+\sum c_{i}\mu_{i}^{\Theta}$ (6)

$f_{elec}\left( \xi,c_{i},\phi_{i} \right)=\sum{z_{i}Fc}_{i}\phi_{i}$ (7)

where $\mu_{i}^{\Theta}$ is the reference chemical potential of species i, $z_{i}$ is the valence of species i, $R$ is the molar gas constant, $T$ is the temperature, $F$ is the Faraday constant, and $g\left( \xi\right)$ is the arbitrary double well function ($g\left( \xi\right)=W\xi^{2}\left( 1-\xi\right)^{2})$ with $W$ being the barrier height.

The electrochemical reaction (${Zn}^{2+}+{2e}^{-}\to Zn$) under the driving force of Equation (1) can be deduced from the Butler−Volmer equation, expressed as

$\frac{\partial\xi}{\partial t}=-L_{\sigma}\left( g^{'}\left( \xi\right)+{f^{'}}_{grad}\left( \xi\right) \right)-L_{\eta}h^{'}\left( \xi\right)\left( e^{\frac{2\left( 1-\alpha\right)F\eta}{RT}}-\frac{c_{{Zn}^{2+}}}{c_{0}}e^{\frac{-2\alpha F\eta}{RT}} \right)$ (8)

where $L_{\sigma}$ is the interfacial mobility, $L_{\eta}$ is the reaction constant, $\alpha$ and $1-\alpha$ are the charge-transfer coefficients, $c_{0}$ is the initial concentration of the electrolyte, $h\left( \xi\right)=\xi^{3}\left( 6\xi^{2}-15\xi+10 \right)$ is an interpolating function, and $\eta=\phi_{Zn}-\phi_{e}-E_{eq}$ is the overpotential ($\phi_{Zn}$, $\phi_{e}$, and $E_{eq}$ denote the potential of the zinc metal anode, the potential of the electrolyte, and the equilibrium potential of electrochemical reaction, respectively). The electrochemical reaction is divided into two parts. The front half ($-L_{\sigma}\left( g^{'}\left( \xi\right)+{f^{'}}_{grad}\left( \xi\right) \right)$) corresponds to the interfacial energy, while the second half $(-L_{\eta}h^{'}\left( \xi\right)\left( e^{\frac{2\left( 1-\alpha\right)F\eta}{RT}}-\frac{c_{{Zn}^{2+}}}{c_{0}}e^{\frac{-2\alpha F\eta}{RT}} \right))$is related to the electrode reaction affinity. The evolution of $c_{{Zn}^{2+}}$ in the electrolyte was described by the Nernst−Planck equation:

$\frac{\partial c_{{Zn}^{2+}}}{\partial t}=\nabla\cdot\left( D_{{Zn}^{2+}}\nabla c_{{Zn}^{2+}}+\frac{2FD_{{Zn}^{2+}}c_{{Zn}^{2+}}}{RT}\nabla\phi_{e} \right)-c_{Zn}\frac{\partial\xi}{\partial t}$ (9)

where $D_{{Zn}^{2+}}$ represents the diffusion coefficient of ${Zn}^{2+}$, and $c_{Zn}$ refers to the initial concentration of electrode. Both diffusion and electromigration were considered. The electrostatic potential distribution can be expressed by

$\nabla\cdot\left( -\sigma_{eff}\nabla\phi_{e} \right)=0$ (10)

$\nabla\cdot\left( \sigma_{eff}\nabla\phi_{e} \right)=2Fc_{Zn}\frac{\partial\xi}{\partial t}$ (11)

Herein, $\sigma_{eff}=h\left( \xi\right)\sigma_{Zn}+\left( 1-h\left( \xi\right) \right)\sigma_{e}$ is the effective electric conductivity, where $\sigma_{Zn}$ and $\sigma_{e}$ represent the electric conductivity of electrode and electrolyte, respectively.

**Supporting Figures**

**
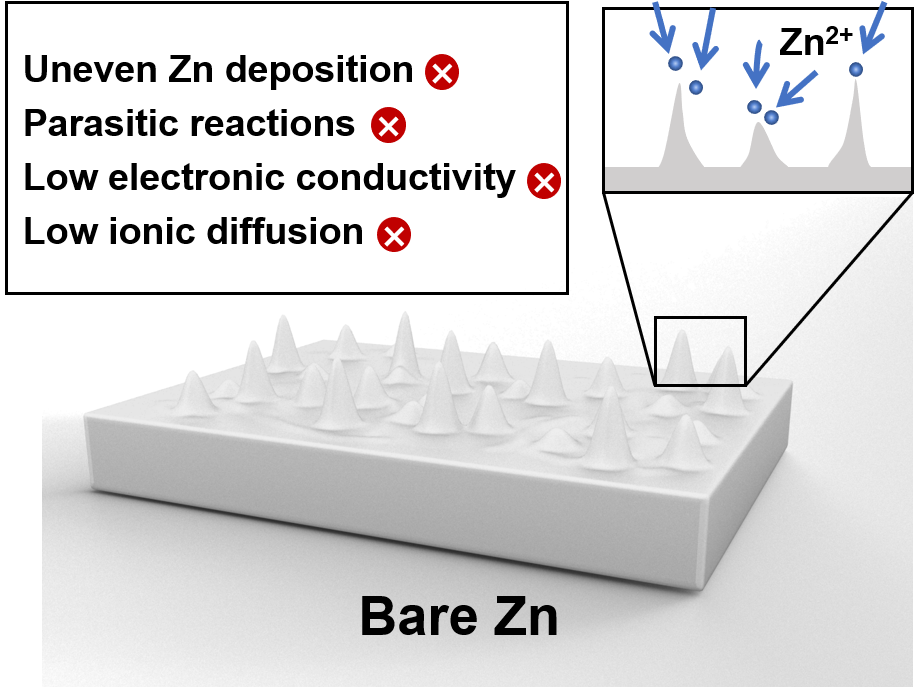
**

**Figure S1.** Schematic diagram of the deposition on the bare Zn anode.

**
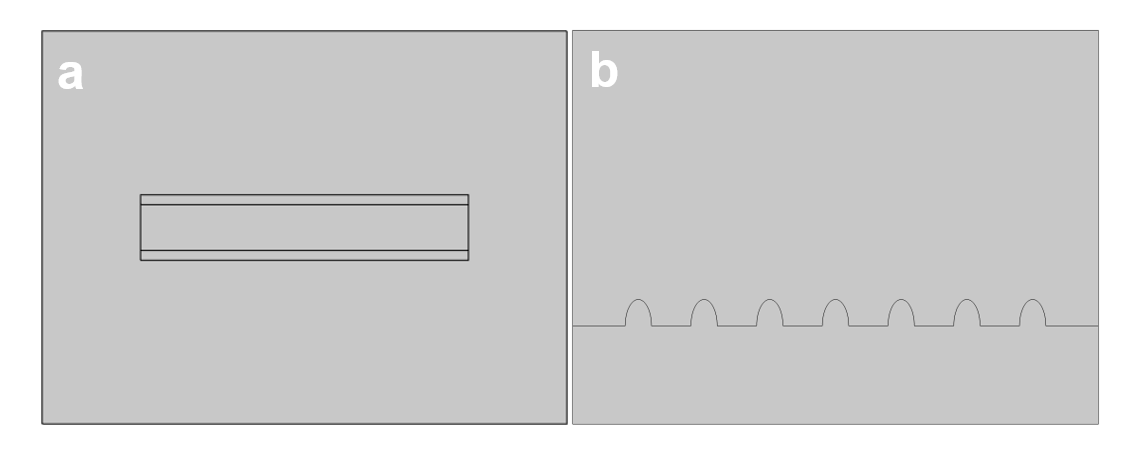
**

**Figure S2.** COMSOL physical model for simulating **a.** Zn deposition in pores, and **b.** surface electric and concentration fields.

**
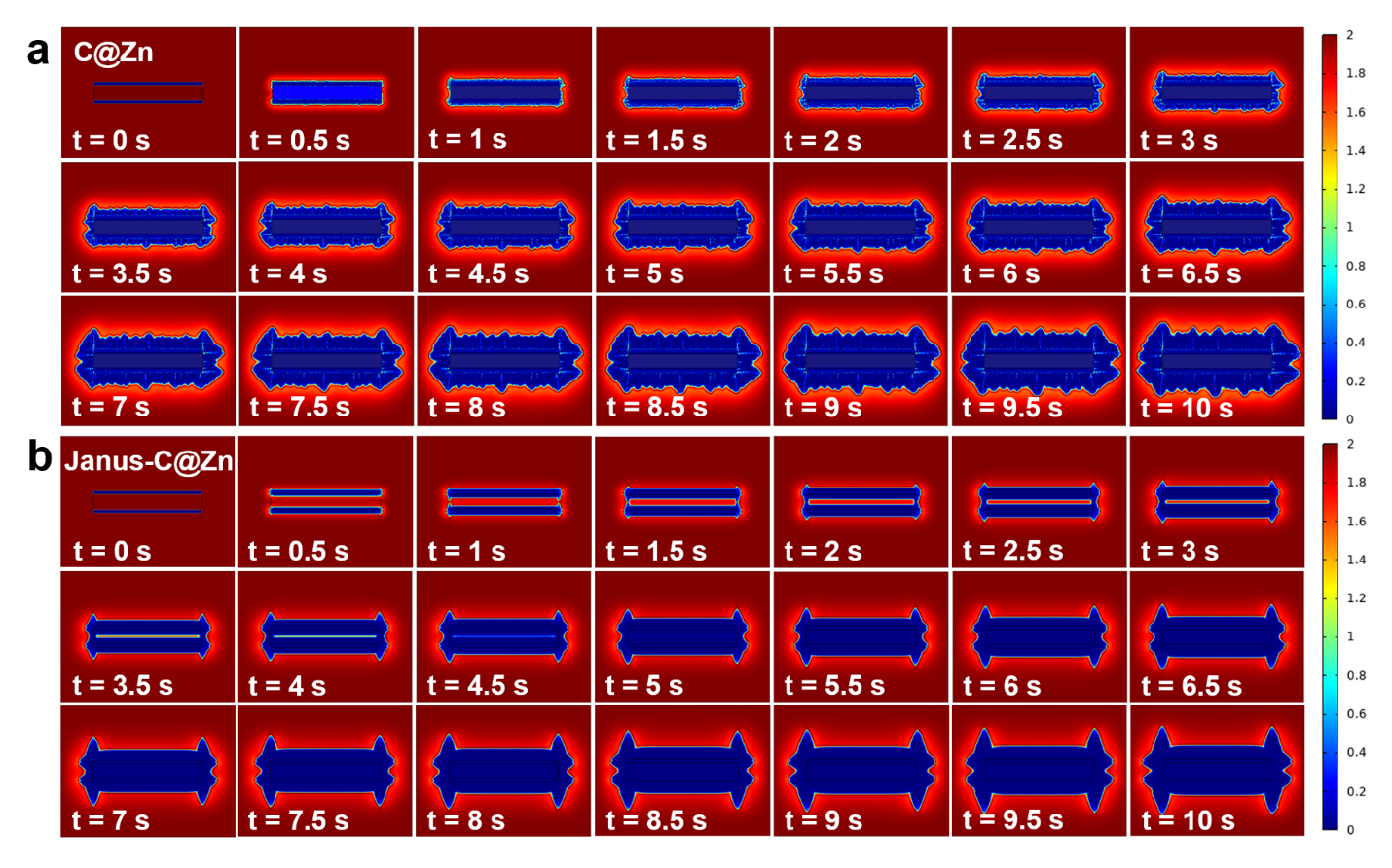
**

**Figure S3.** COMSOL simulation of Zn^2+^ concentration inside and outside channels at 0-10s in **a.** C and **b.** Janus-C.

Zn^2+^ is primarily located outside of the channels in the untreated porous carbon, which increases with time. The Janus carbon structure, however, displays a high Zn^2+^ concentration in the inner channel, indicating Zn will be initially deposited in the inner pores.

**
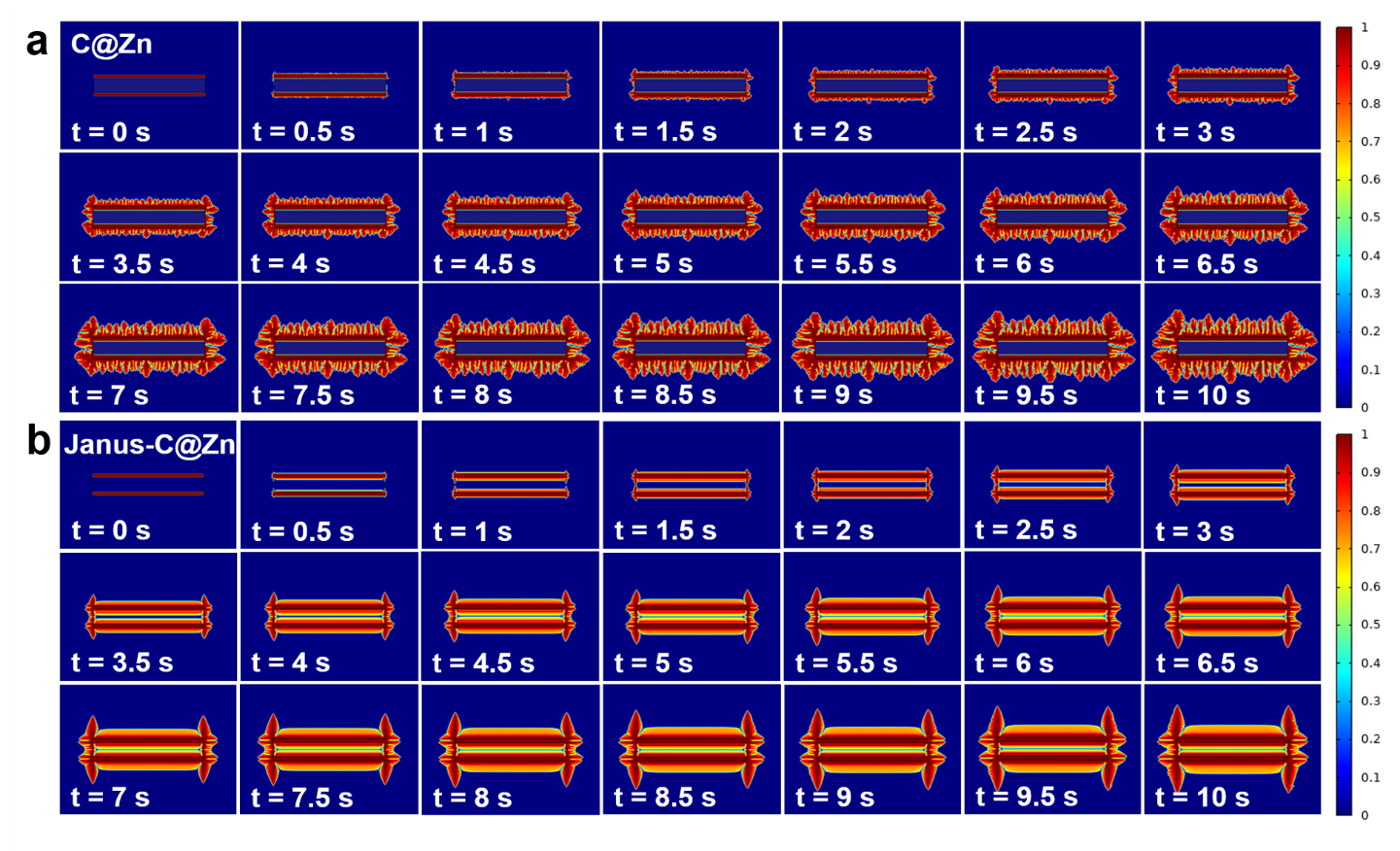
**

**Figure S4.** COMSOL simulation of zinc dendrites growth inside and outside pores during deposition of Zn^2+^ at 0-10s in **a.** C and **b.** Janus-C.

Similar to the concentration map in Figure S3, Zn is primarily deposited on the outside surface of the untreated carbon surface and the inner pores of the Janus carbon.

**
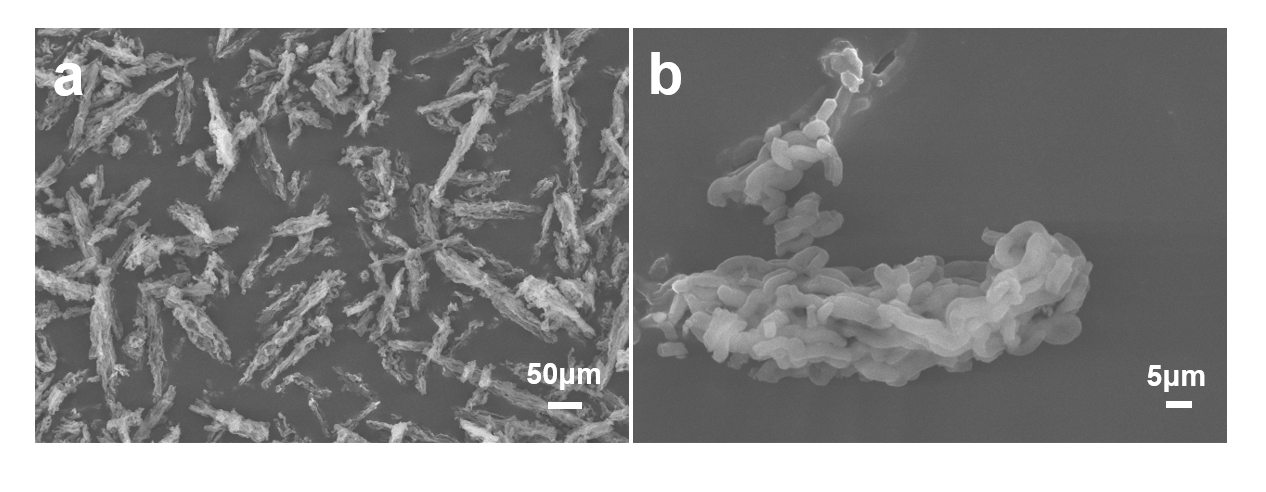
**

**Figure S5.** SEM image of SBA-15 at **a.** low and **b.** high magnification.


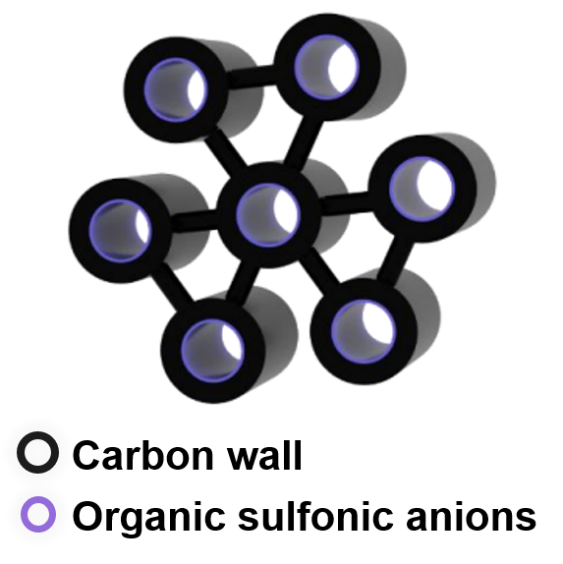


**Figure S6.** Structural schematic diagram of the synthesis of CMK-5/PS.

The organic functional groups are decorated in the tubular pores of CMK-5.

**
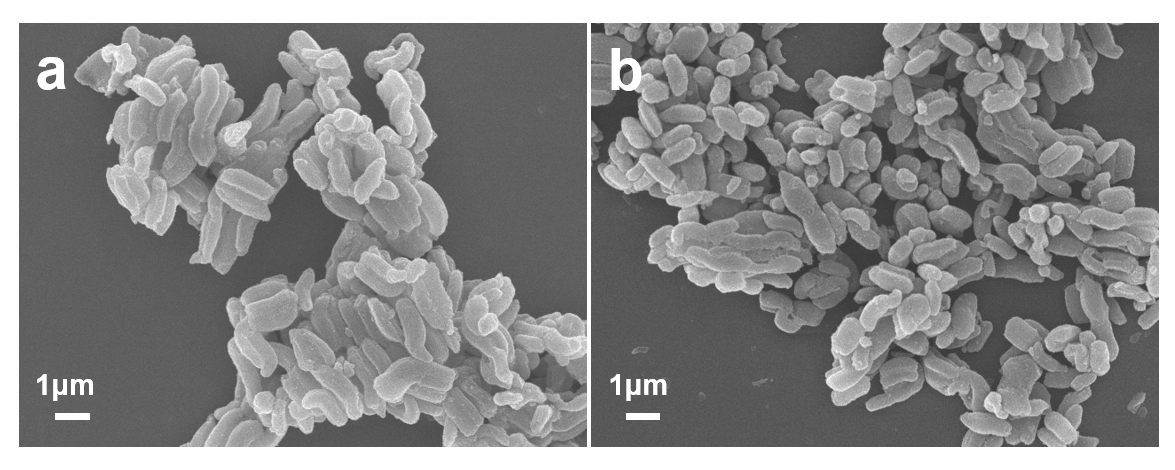
**

**Figure S7.** SEM images of **a.** CMK-5 and **b.** CMK-5/PS.

**
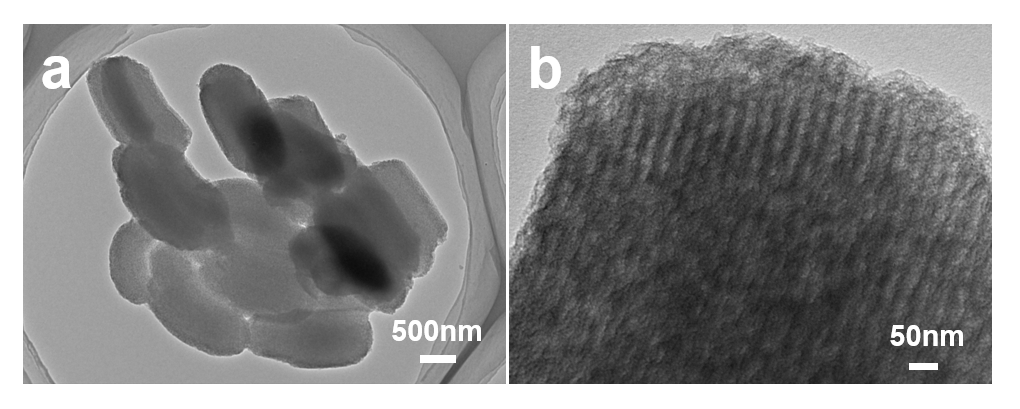
**

**Figure S8.** TEM of CMK-5 at **a.** low and **b.** high magnification.

**
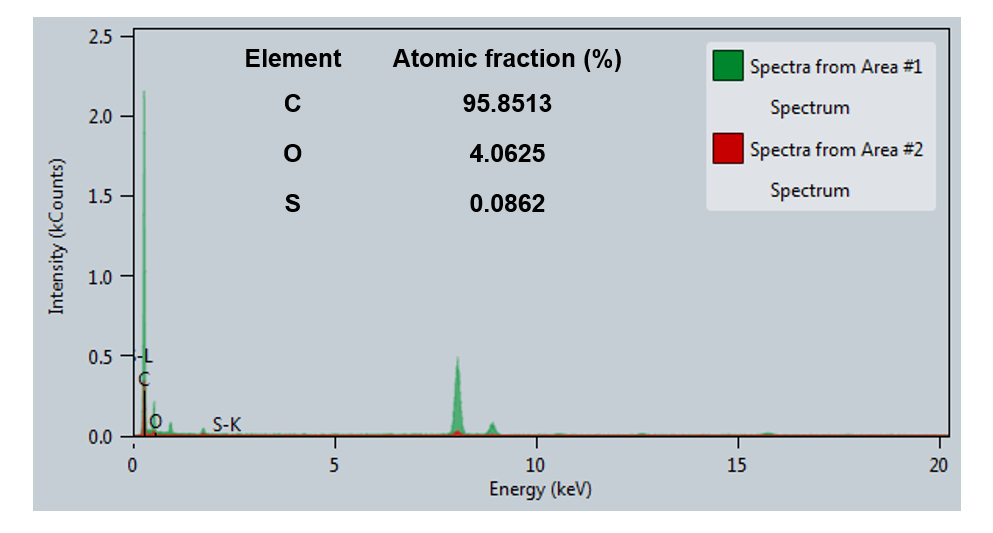
**

**Figure S9.** Element mapping of CMK-5/PS.

**
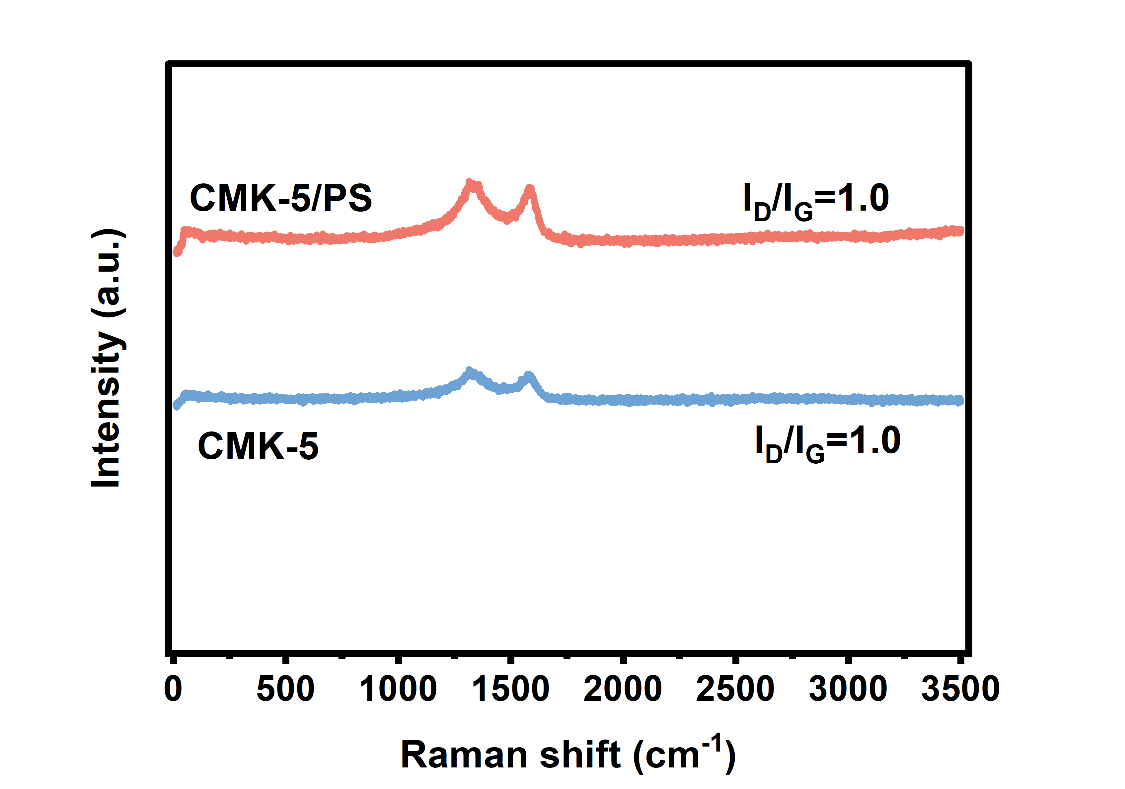
**

**Figure S10.** Raman spectra of CMK-5 and CMK-5/PS.

The Raman spectrum shows two prominent peaks near 1330 cm^-1^ and 1580 cm^-1^, which belong to the D band of carbon material defects and partial amorphous state and the G band related to graphitic carbon.^[9,10]^ The relative intensity ratio of I_D_/I_G_ is about 1.0, indicating that the prepared mesoporous carbon material is mainly amorphous, which is consistent with the XRD results.

**
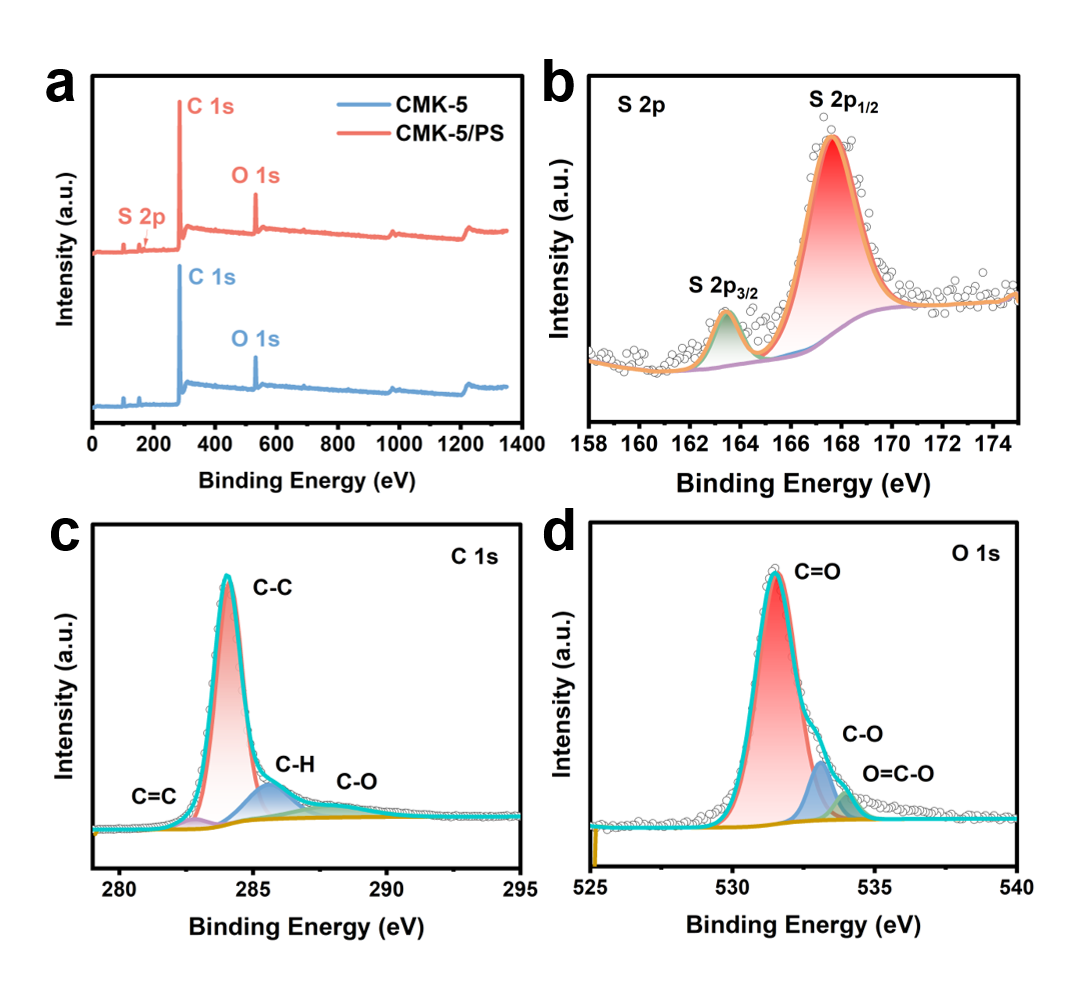
**

**Figure S11.** XPS test of CMK-5 and CMK-5/PS with **a.** full-spectrum elements, **b.** S in organic modified carbon materials, **c.** C 1s and **d.** O 1s.


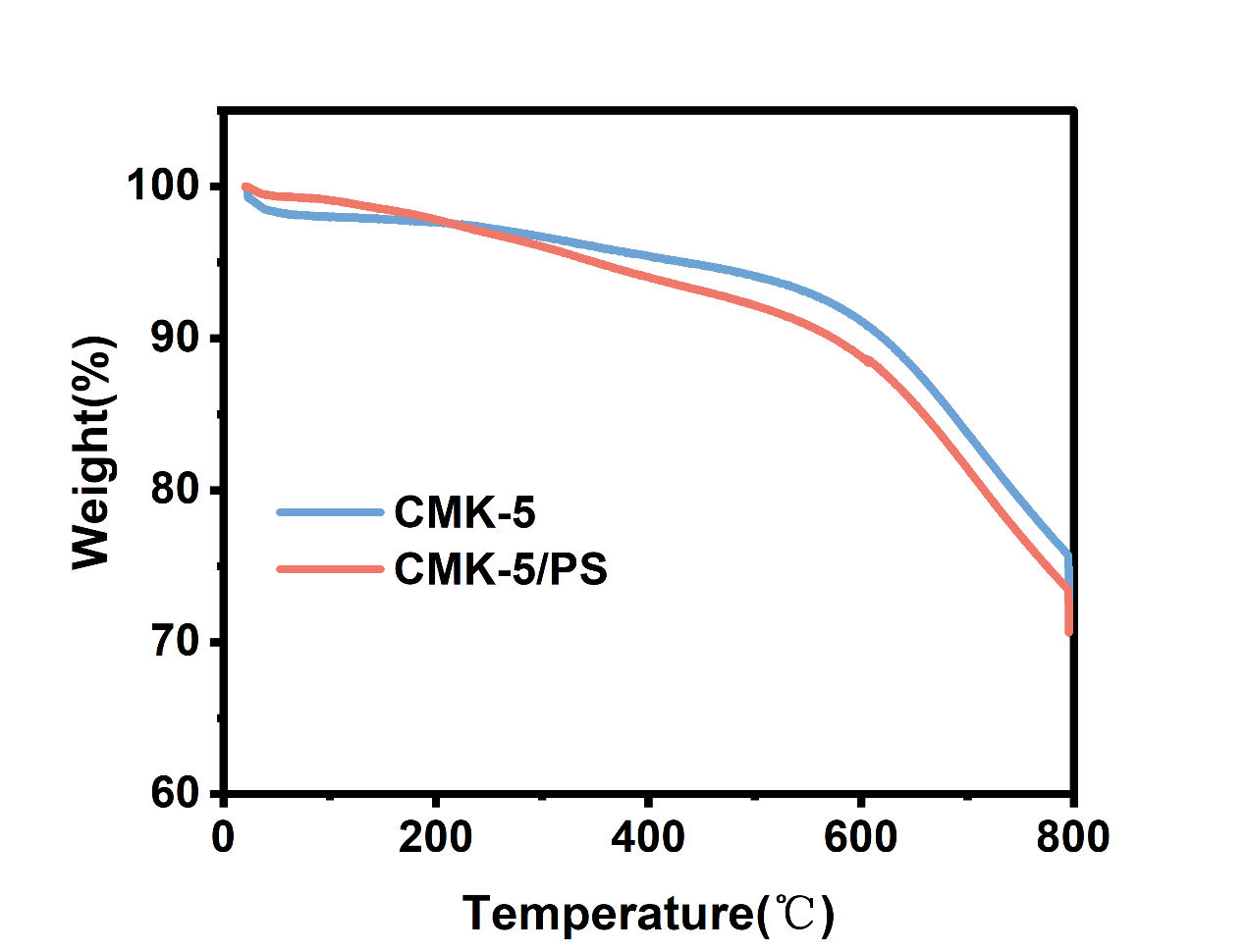


**Figure S12.** TGA analysis of carbon materials with and without organic modifications.

**
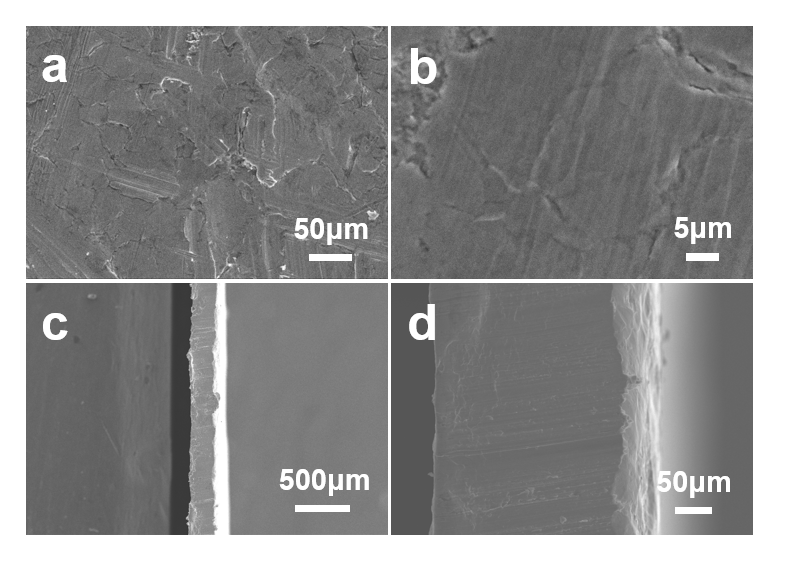
**

**Figure S13.** SEM images of **a-b.** bare Zn foil at plane and **c-d.** cross-section.

**
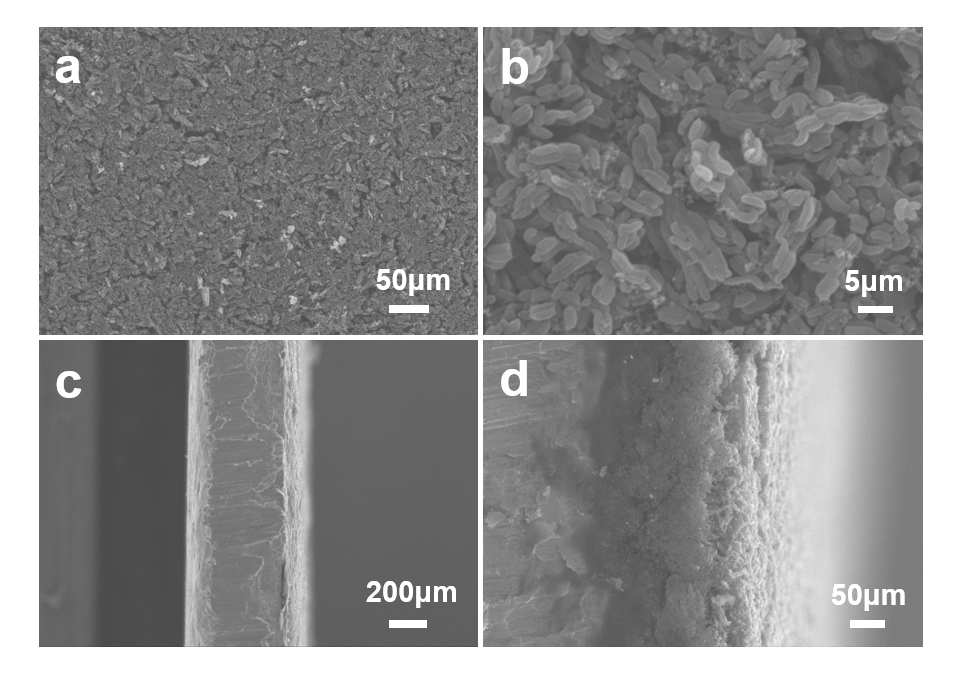
**

**Figure S14.** SEM images of **a-b.** CMK-5/PS@Zn at plane and **c-d.** cross-section.

**
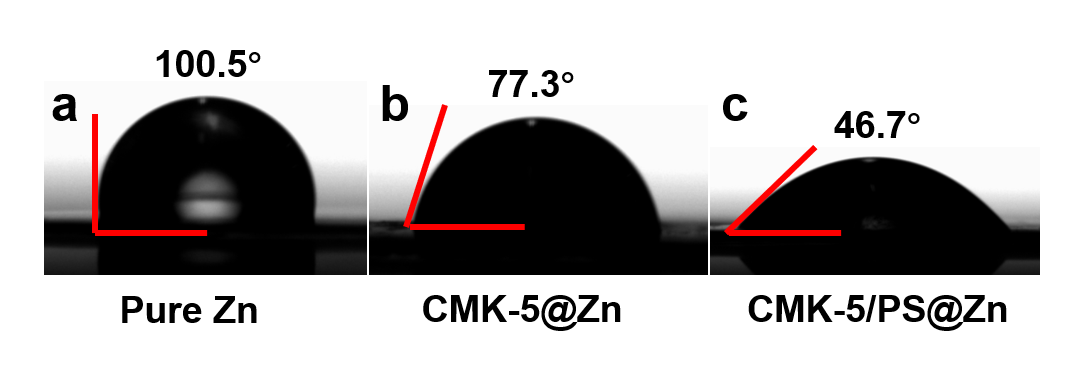
**

**Figure S15.** Contact angles of **a.** bare Zn anode, **b.** CMK-5@Zn anode and **c.** CMK-5/PS@Zn anode.


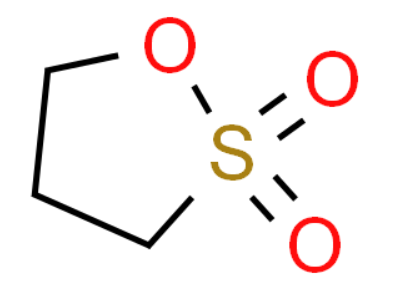


**Figure S16.** Structure diagram of 1, 3-propanolactone.

**
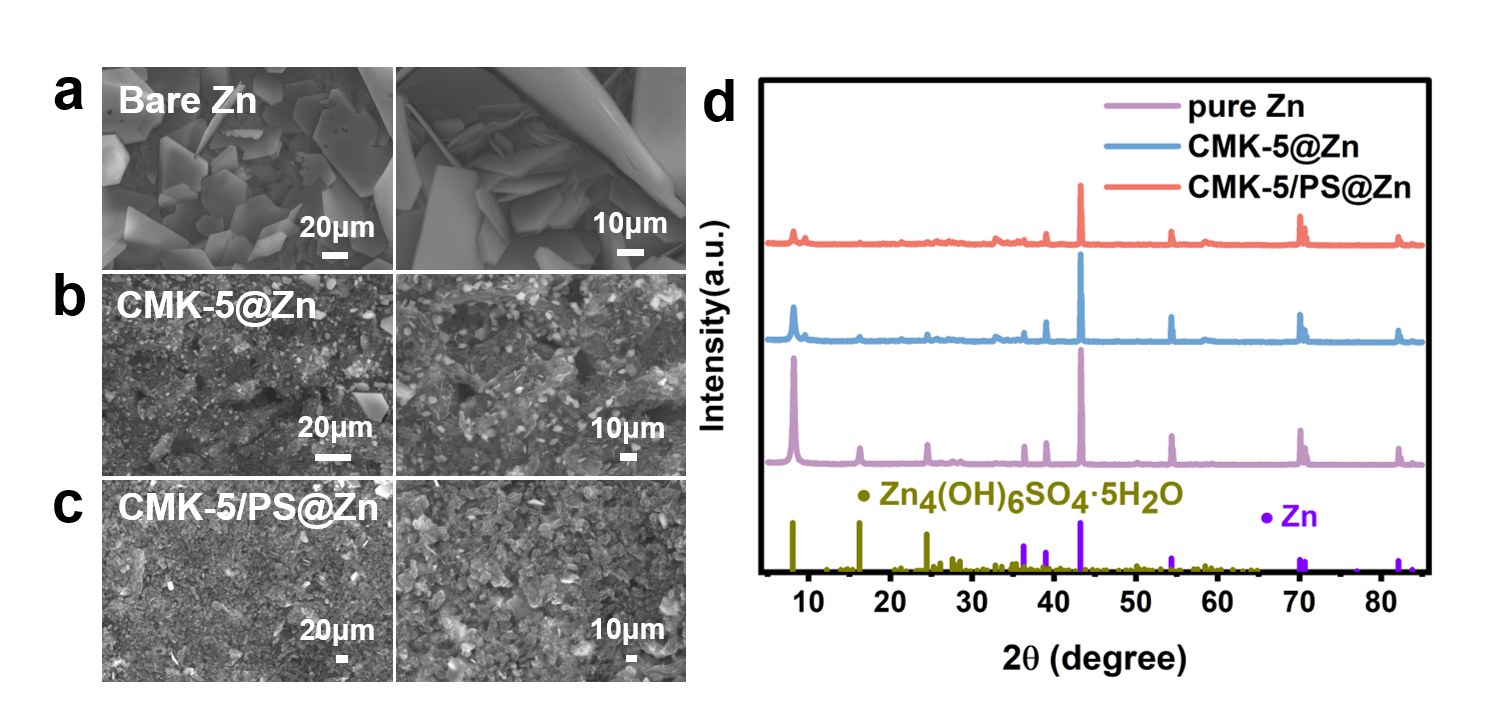
**

**Figure S17.** The aging experiment by immersing the Zn anodes in the electrolytes for 7 days. SEM images of **a.** bare Zn anode, **b.** CMK-5@Zn anode and **c.** CMK-5/PS@Zn anode aged in 2M ZnSO_4_ electrolyte for 7 days. **d.** XRD images of bare Zn anode, CMK-5@Zn anode and CMK-5/PS@Zn anode aged in 2M ZnSO_4_ electrolyte for 7 days.

**
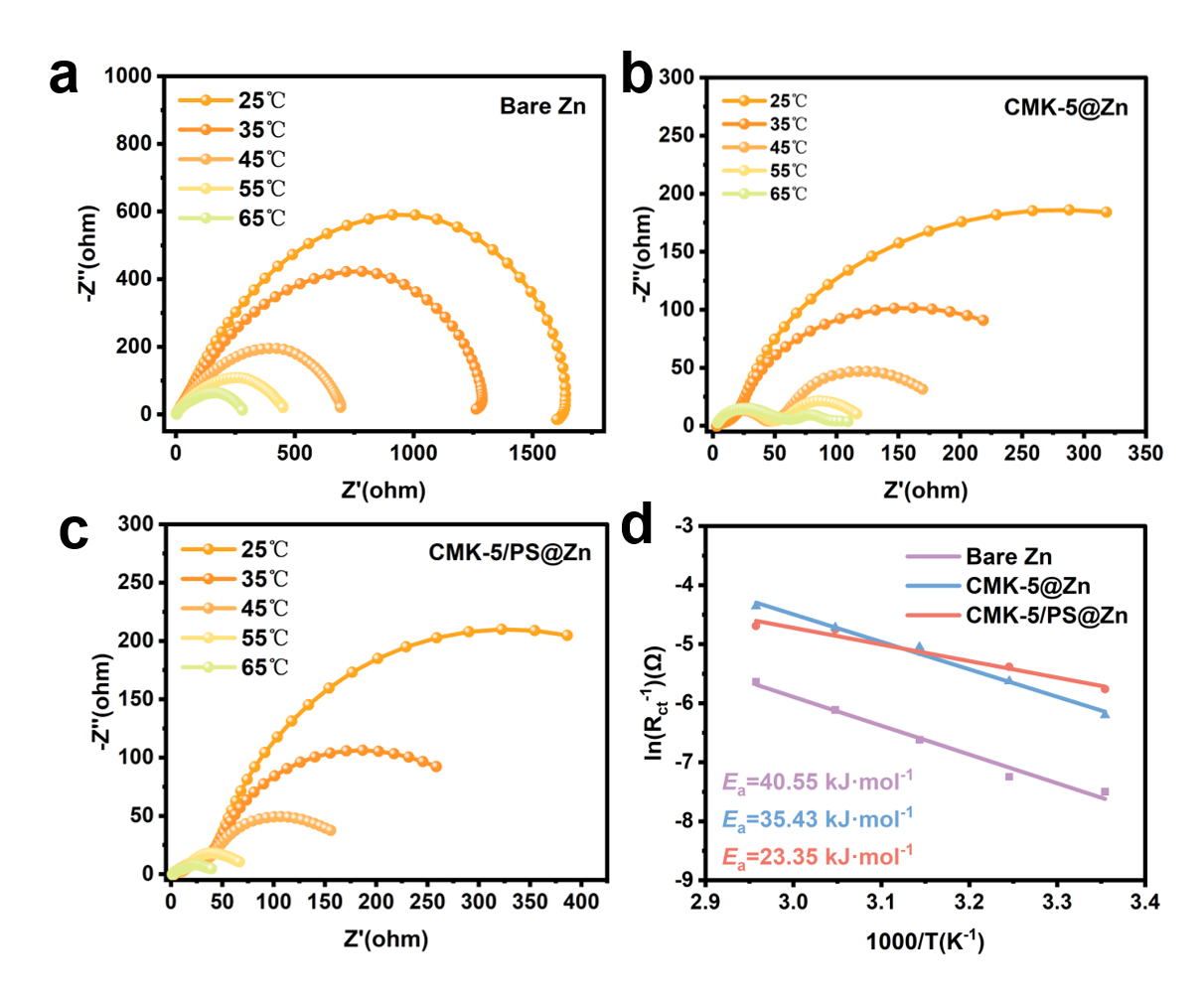
**

**Figure S18.** Nyquist diagram of **a.** bare Zn, **b.** CMK-5@Zn, and **c.** CMK-5/PS@Zn anodes at different temperatures. **d.** Comparison of Nyquist curves and activation energies of different Zn anodes.

In order to further investigate the effect of CMK-5/PS coating on the charge transfer process of Zn electrodes, the transport behaviors of bare Zn, CMK-5@Zn and CMK-5/PS@Zn electrodes were further tested, and the corresponding Zn^2+^ activation energy (E_a_) was calculated from the AC impedance spectroscopy (EIS) of symmetric batteries at different temperatures. The calculation is based on the Arrhenius equation:^[11]^

$\frac{1}{R_{ct}}=A exp(-\frac{E_{a}}{RT})$ (12)

Where R_ct_ is the interface resistance, A refers to the pre-factor, E_a_, R and T are respectively the activation energy, molar gas constant and absolute temperature.

**
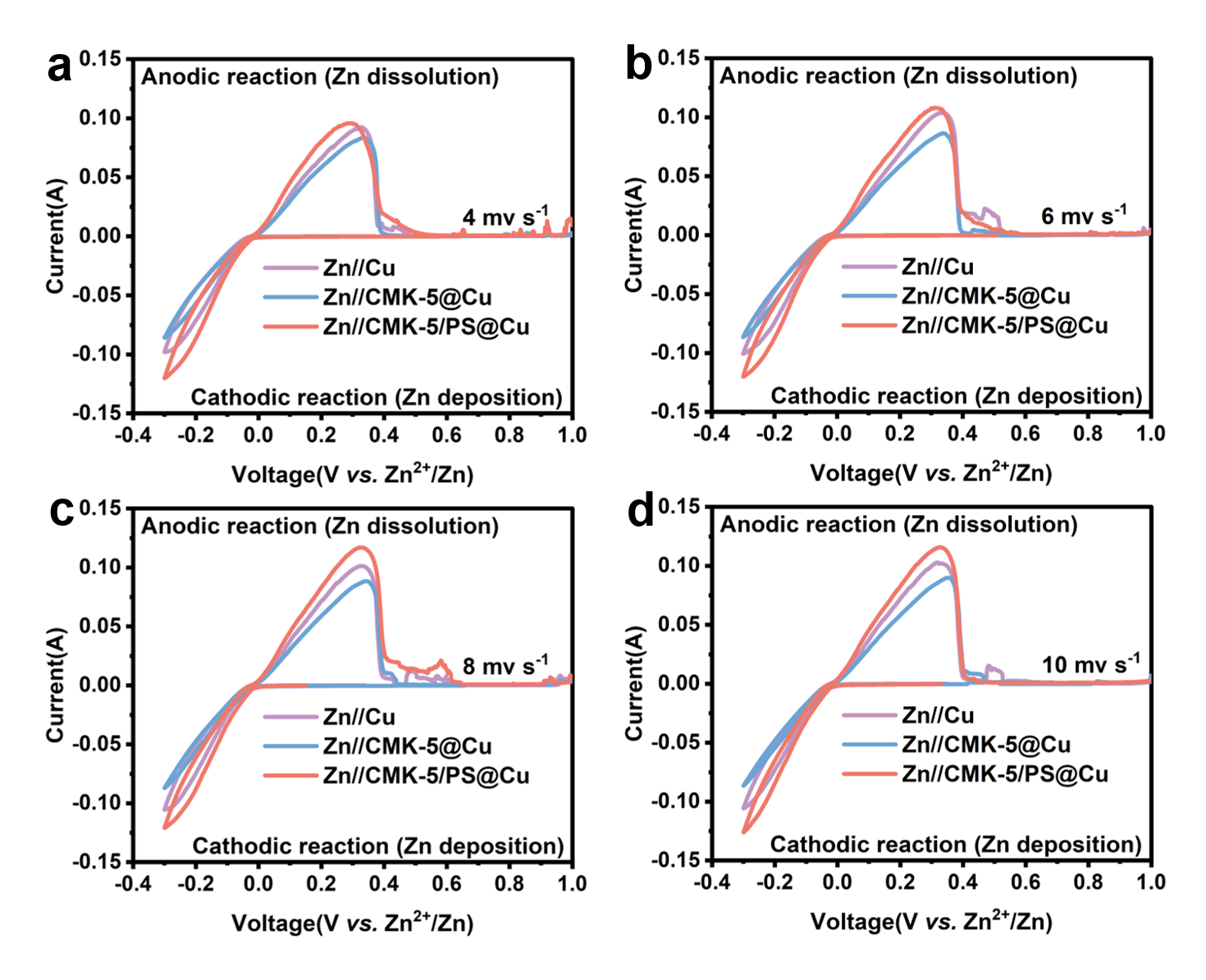
**

**Figure S19.** Comparison of CV curves of different Cu cathodes at the same sweep speed with **a.** 4 mV s^-1^, **b.** 6 mV s^-1^, **c.** 8 mV s^-1^, **d.** 10 mV s^-1^.


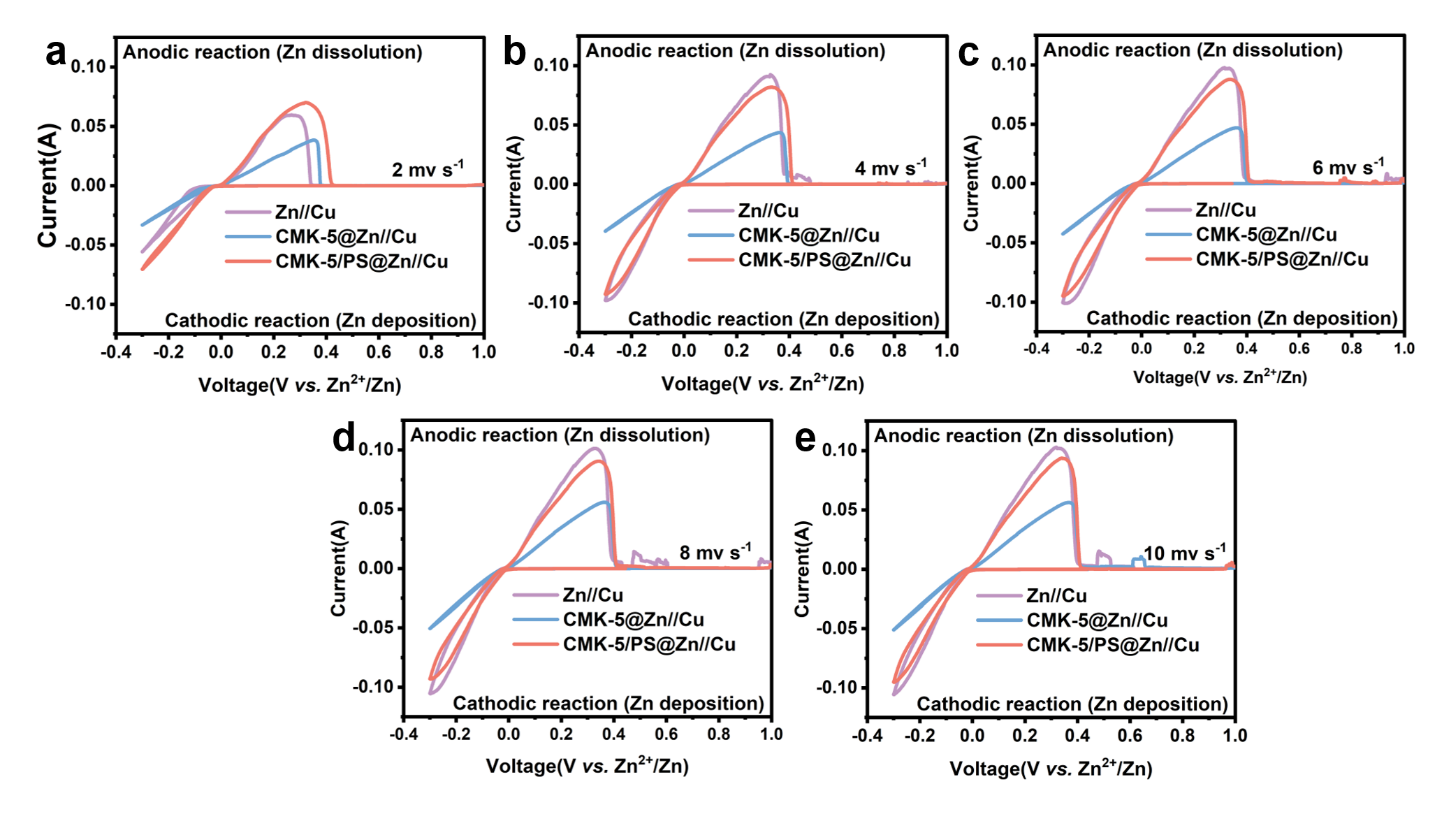


**Figure S20.** Comparison of CV curves of different Zn anodes at the same sweep speed with **a.** 2 mV s^-1^, **b.** 4 mV s^-1^, **c.** 6 mV s^-1^, **d.** 8 mV s^-1^, **e.** 10 mV s^-1^.

**
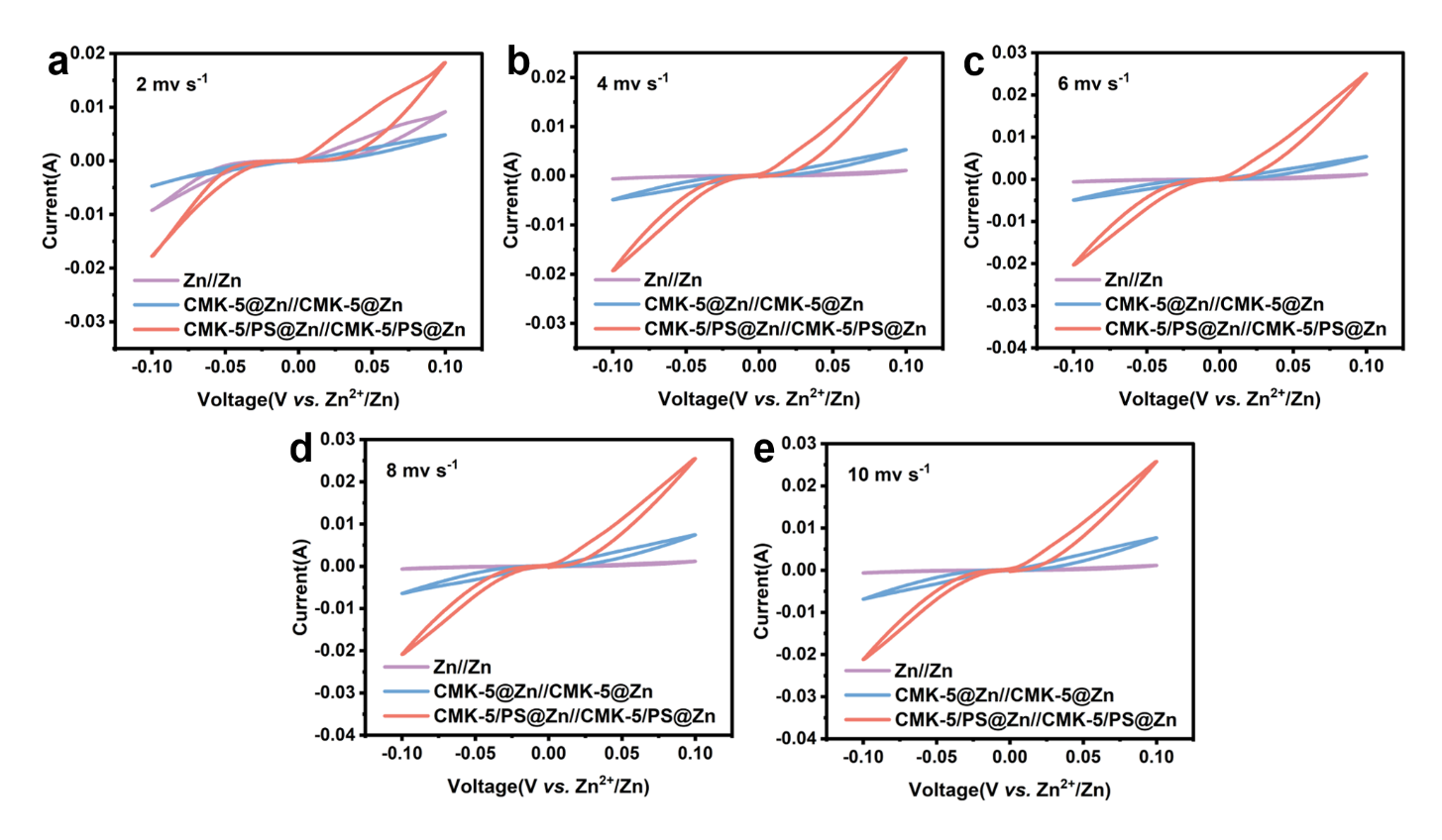
**

**Figure S21.** CV curves of different Zn anode Symmetrical cells at different scanning speeds with **a.** 2 mV s^-1^, **b.** 4 mV s^-1^, **c.** 6 mV s^-1^, **d.** 8 mV s^-1^, **e.** 10 mV s^-1^.

Cyclic voltammetry (CV) curves for symmetric cells (Zn//Zn, CMK-5@Zn//CMK-5@Zn and CMK-5/PS@Zn//CMK-5/PS@Zn) demonstrate that the CMK-5/PS@Zn//CMK-5/PS@Zn symmetric cell exhibits the highest current density, indicating enhanced interfacial activity for Zn deposition.

**
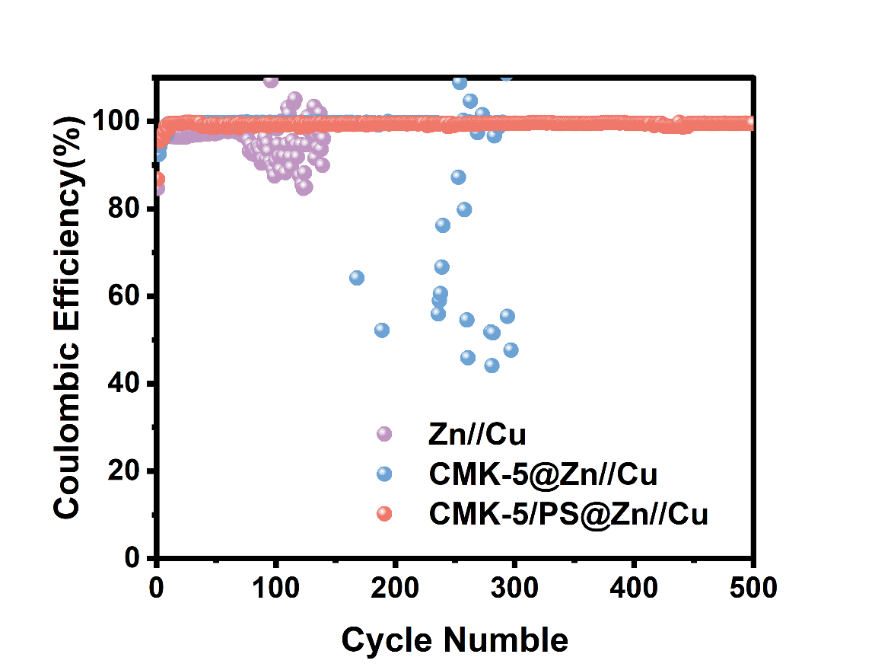
**

**Figure 22.** Electroplating/stripping Coulomb efficiencies of CMK-5/PS@Zn//Cu cells, CMK-5@Zn//Cu cells and bare Zn//Cu cells at 1 mA cm^-2^-1 mAh cm^-2^.

CMK-5/PS@Zn//Cu cells also show stable deposition behavior over 500 cycles with an average CE of 99.81%, which is much better than CMK-5@Zn//Cu cells and bare Zn//Cu cells.


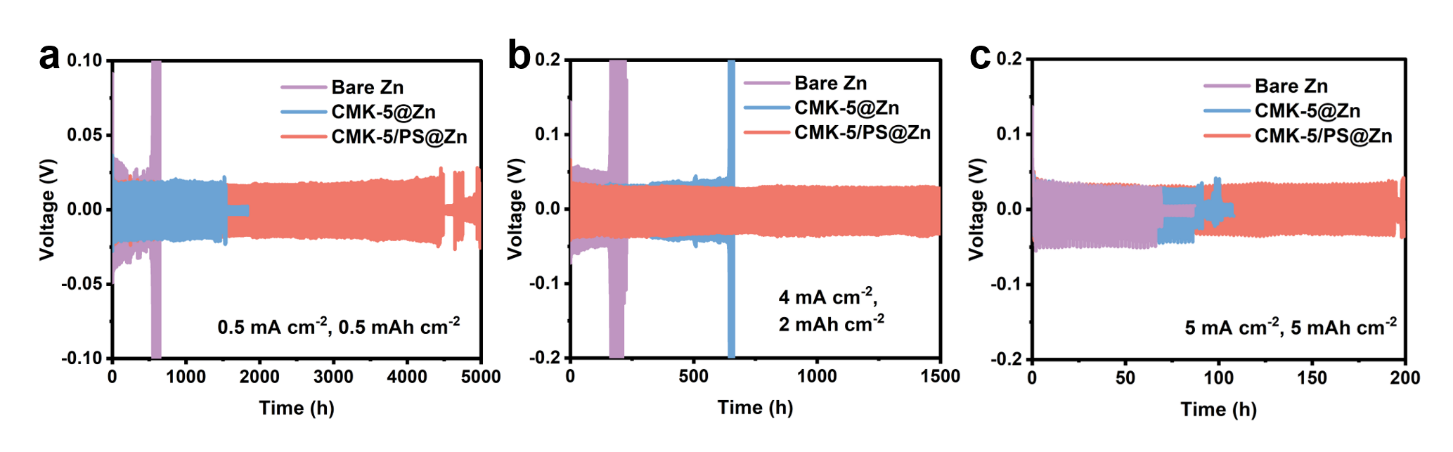


**Figure 23.** Cycle performance of CMK-5/PS@Zn//CMK-5/PS@Zn, CMK-5@Zn// CMK-5@Zn, Zn//Zn symmetric batteries at **a.** 0.5 mA cm^-2^-0.5 mAh cm^-2^, **b.** 4 mA cm^-2^-2 mAh cm^-2^, **c.** 5 mA cm^-2^-5 mAh cm^-2^.

**
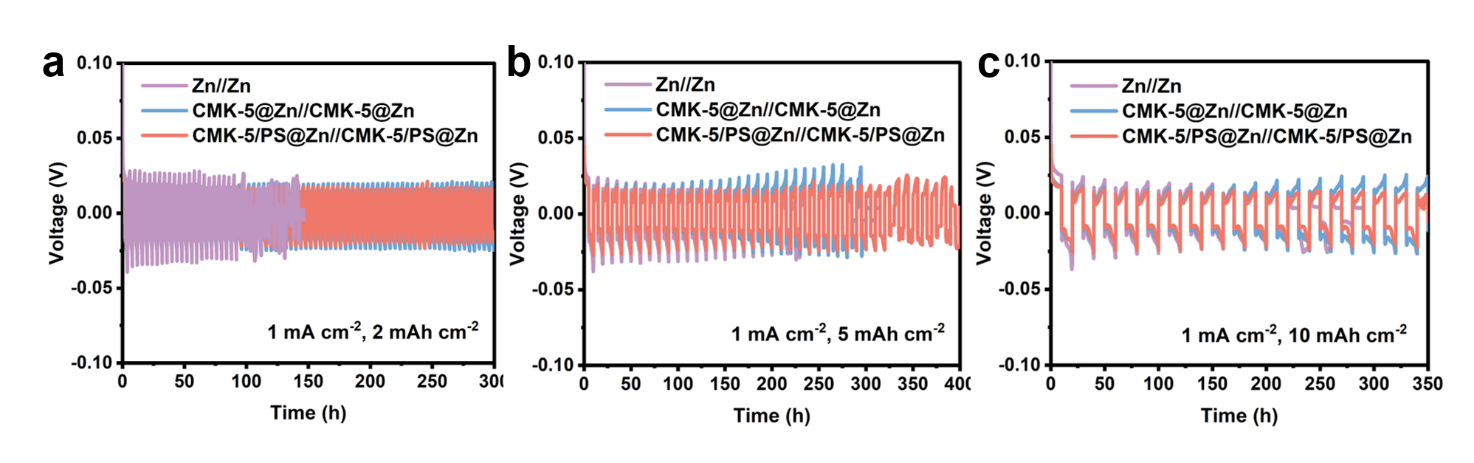
**

**Figure S24.** Cycle performance of CMK-5/PS@Zn//CMK-5/PS@Zn, CMK-5@Zn//CMK-5@Zn and Zn//Zn symmetric batteries at 1 mA cm^-2^ and different capacities of **a.** 2 mAh cm^-2^, **b.** 5 mAh cm^-2^, **c.** 10 mAh cm^-2^.

**
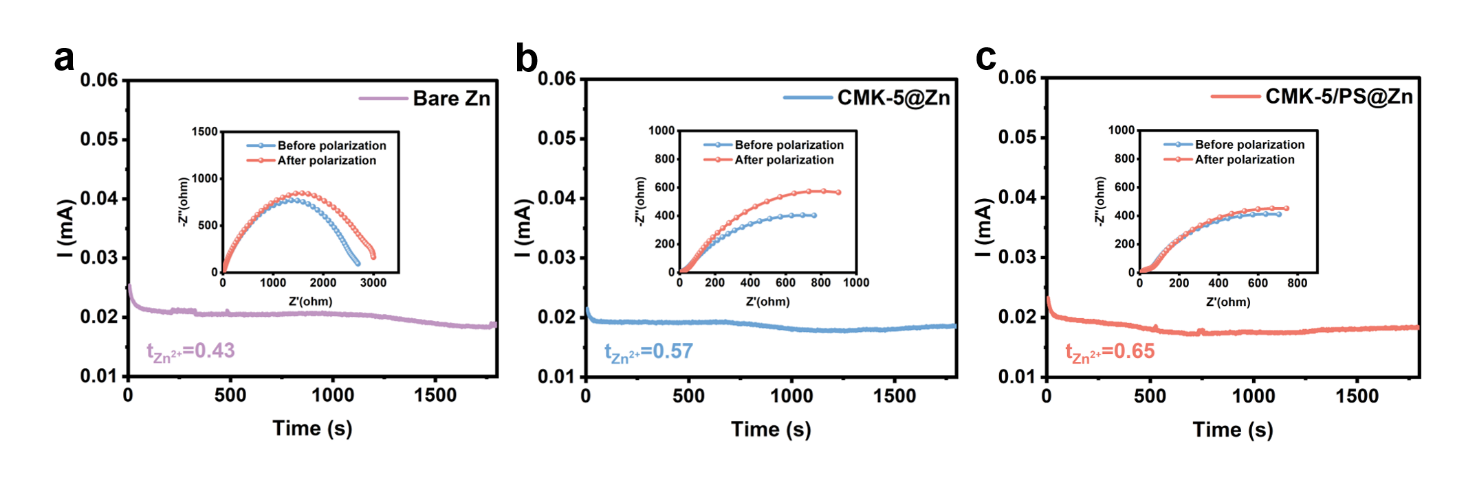
**

**Figure S25.** Current variation during polarization of **a.** bare Zn, **b.** CMK-5@Zn and **c.** CMK-5/PS@Zn symmetric cells before and after polarization. (inset: Nyquist polarization maps of bare Zn, CMK-5@Zn and CMK-5/PS@Zn symmetric cells).

The Zn^2+^ transference number was evaluated in the symmetric cells by EIS measurements before and after polarization. The following is the computational formula:^[12]^

t_Zn_^2+^=$\frac{I_{S}\text{(∆V-}I_{0}R_{0}\text{)}}{I_{0}(\Delta V-I_{S}R_{S})}$ (13)

Where 𝛥𝑉 is the applied voltage (0.01 V), *I_0_* and *R_0_* are the initial current and charge transfer resistance, *Is* and *Rs* refer to the steady-state current and charge transfer resistance.

The calculated Zn^2+^ migration number of CMK-5/PS@Zn is 0.65, which is higher than that of CMK-5@Zn and the bare Zn anode (Figure S24 and Table S3), which can be attributed to the cation-selective ability of the CMK-5/PS layer to prevent the infiltration of SO_4_^2-^ and OH^−^ ions. In addition, as shown in Table S4 (Supporting information), the electronic conductivity of the CMK-5/PS layer is also improved compared to the CMK-5 counterpart.


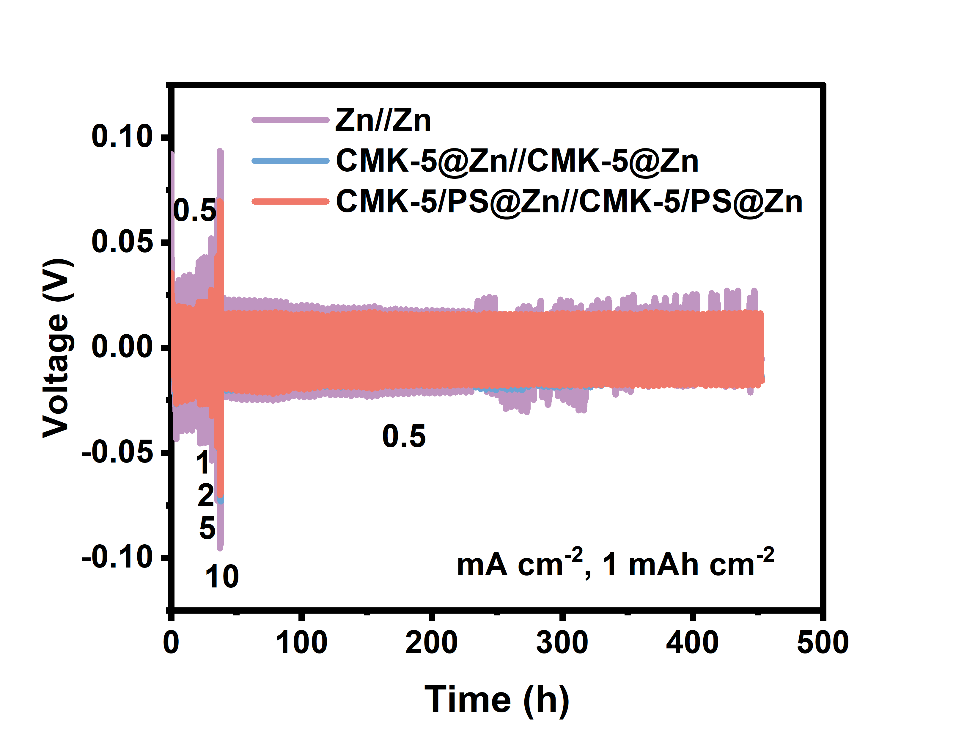


**Figure S26.** Rate performance of symmetric batteries at different current densities.

**
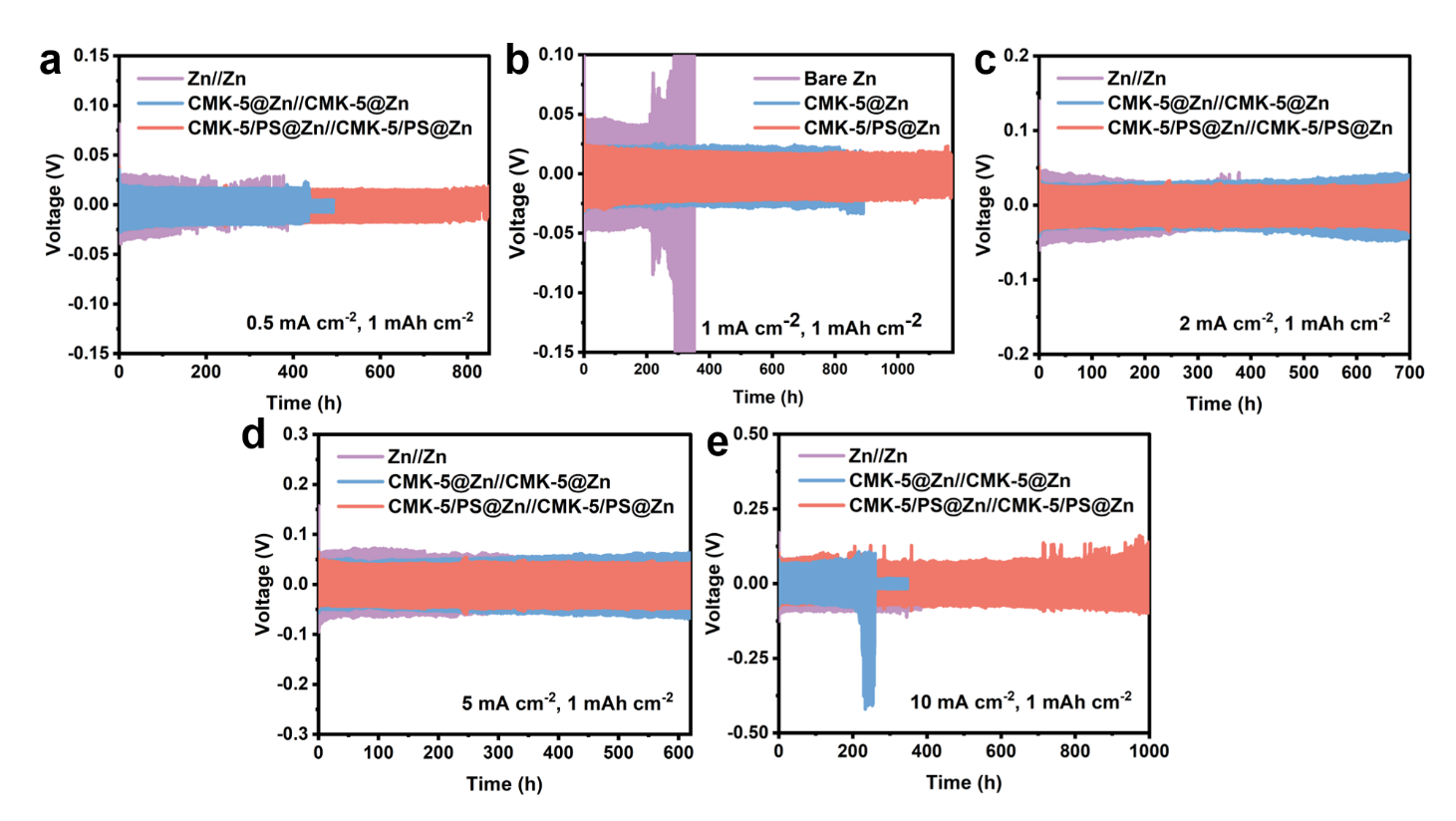
**

**Figure S27.** Long cycle performance of bare Zn, CMK-5@Zn and CMK-5/PS@Zn symmetric batteries with different current densities of **a.** 0.5, **b.** 1, **c.** 2, **d.** 5, **e.** 10 mA cm^-2^ at fixed capacity of 1 mAh cm^-2^.

We tested the durability of the symmetric battery under various current densities when the capacitance is 1 mAh cm^-2^. When the fixed capacitance is 1 mAh cm^-2^, the CMK-5/PS symmetrical cell exhibits low voltage lag and excellent long-cycle performance as the current increases from 0.5 mA cm^-2^ to 10 mA cm^-2^. Especially when the current density is 10 mA cm^-2^, the CMK-5/PS@Zn anode exhibits an excellent cycling durability of ~1000 h, which is much longer than that of CMK-5@Zn anode (~200 h) and bare Zn anode (~400 h). The results show that the CMK-5/PS@Zn anode has lower hysteresis voltage and cycle stability, and the CMK-5/PS coating effectively enhances the Zn^2+^ dynamics.

**
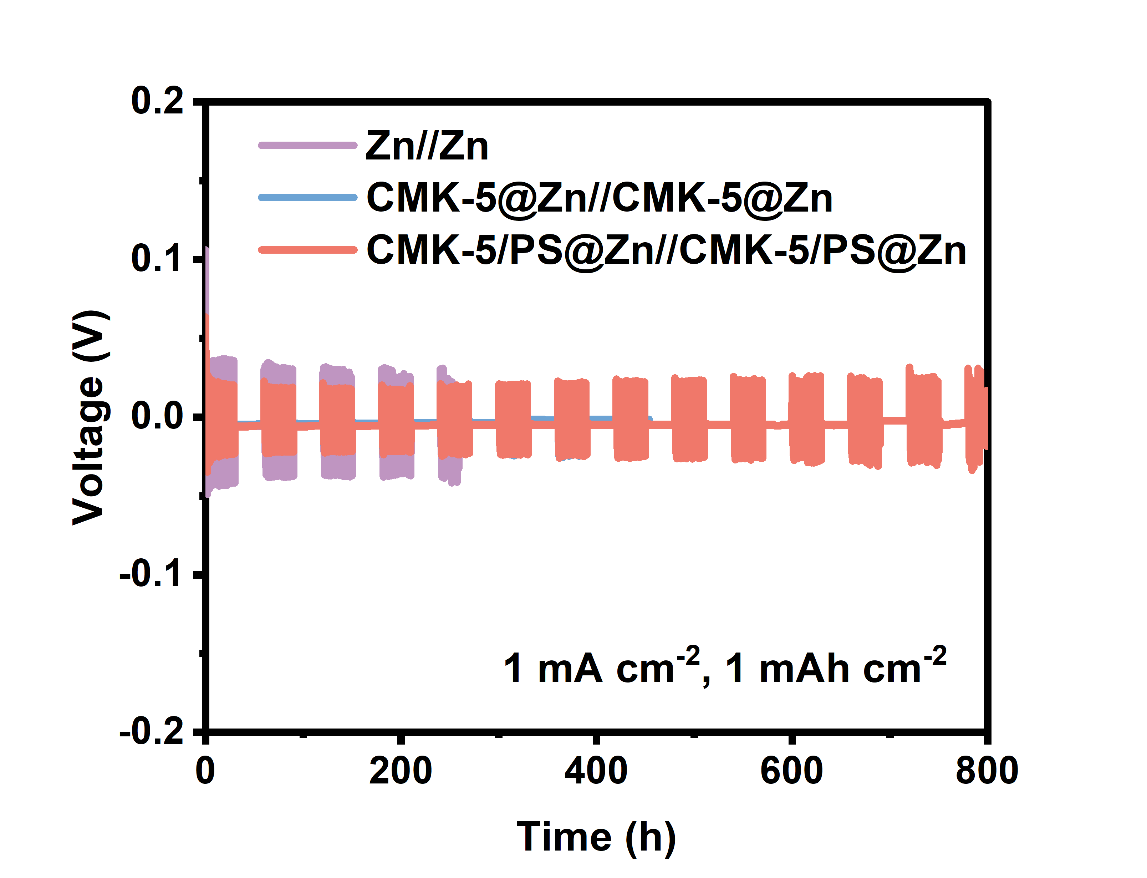
**

**Figure S28.** Shelf-recovery performance of bare Zn, CMK-5@Zn and CMK-5/PS@Zn symmetric batteries at 1 mA cm^-2^-1 mAh cm^-2^.

Shelved recovery tests show that the CMK-5/PS@Zn anode has excellent structural stability and sensitive current response.

**
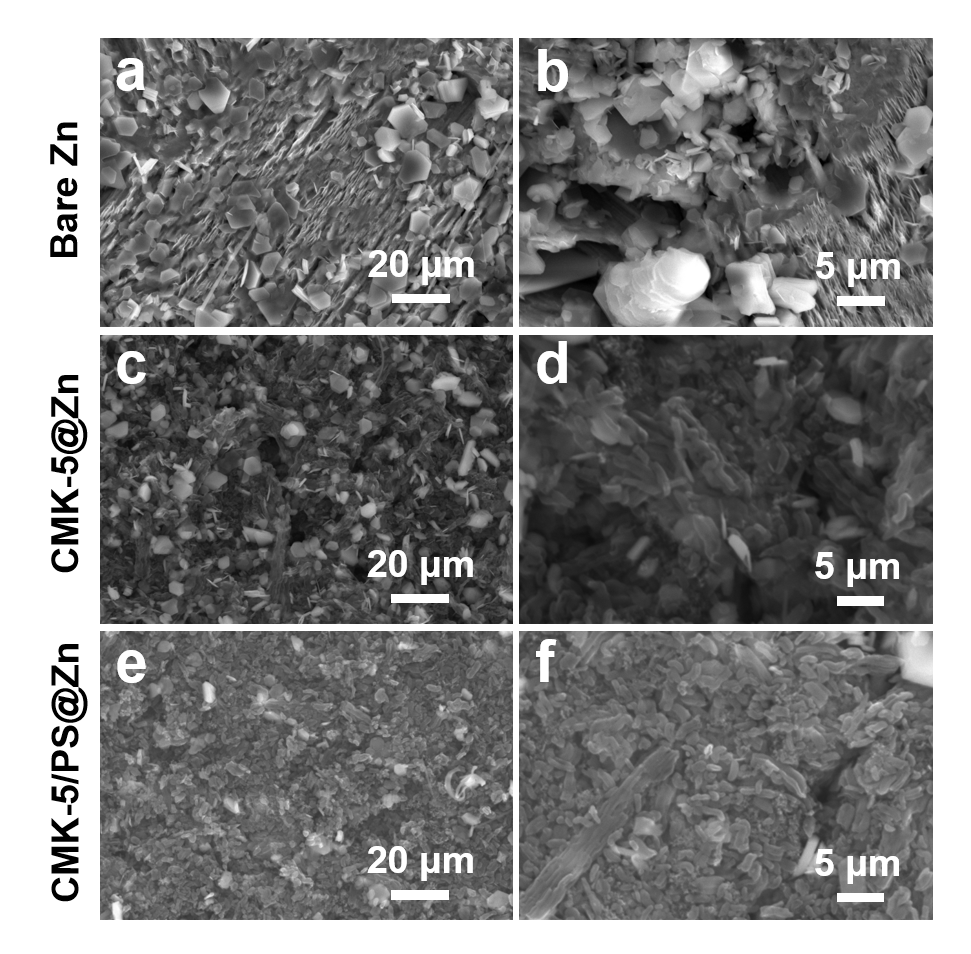
**

**Figure S29.** Top view of a symmetrical battery after 100 cycles at 1 mA cm^-2^/1 mAh cm^-2^. SEM images of **a.** and **b.** Zn//Zn, **c.** and **d.** CMK-5@Zn//CMK-5@Zn, **e.** and **f.** CMK-5/PS@Zn//CMK-5/PS@Zn.

**
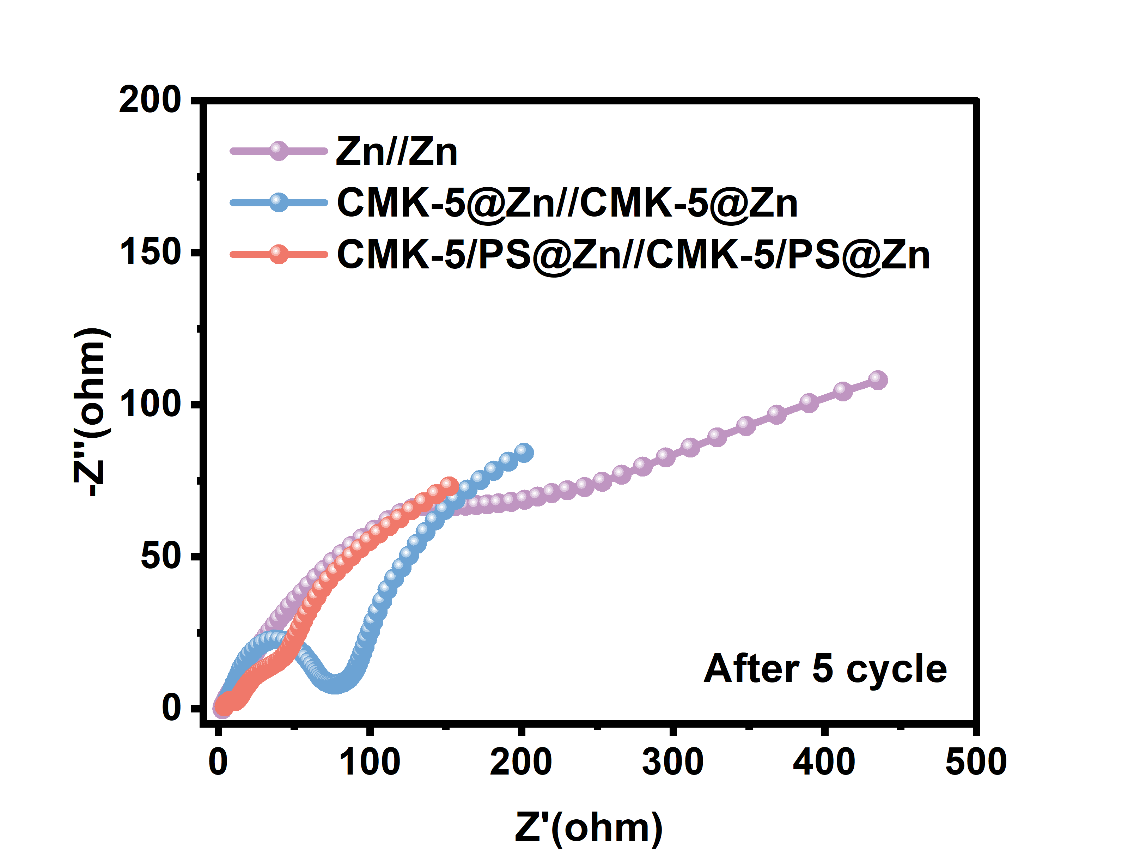
**

**Figure S30.** Zn//Zn, CMK-5@Zn//CMK-5@Zn, CMK-5/PS@Zn//CMK-5@Zn/PS Nyquist diagram after 5 cycles of a symmetric battery.

**
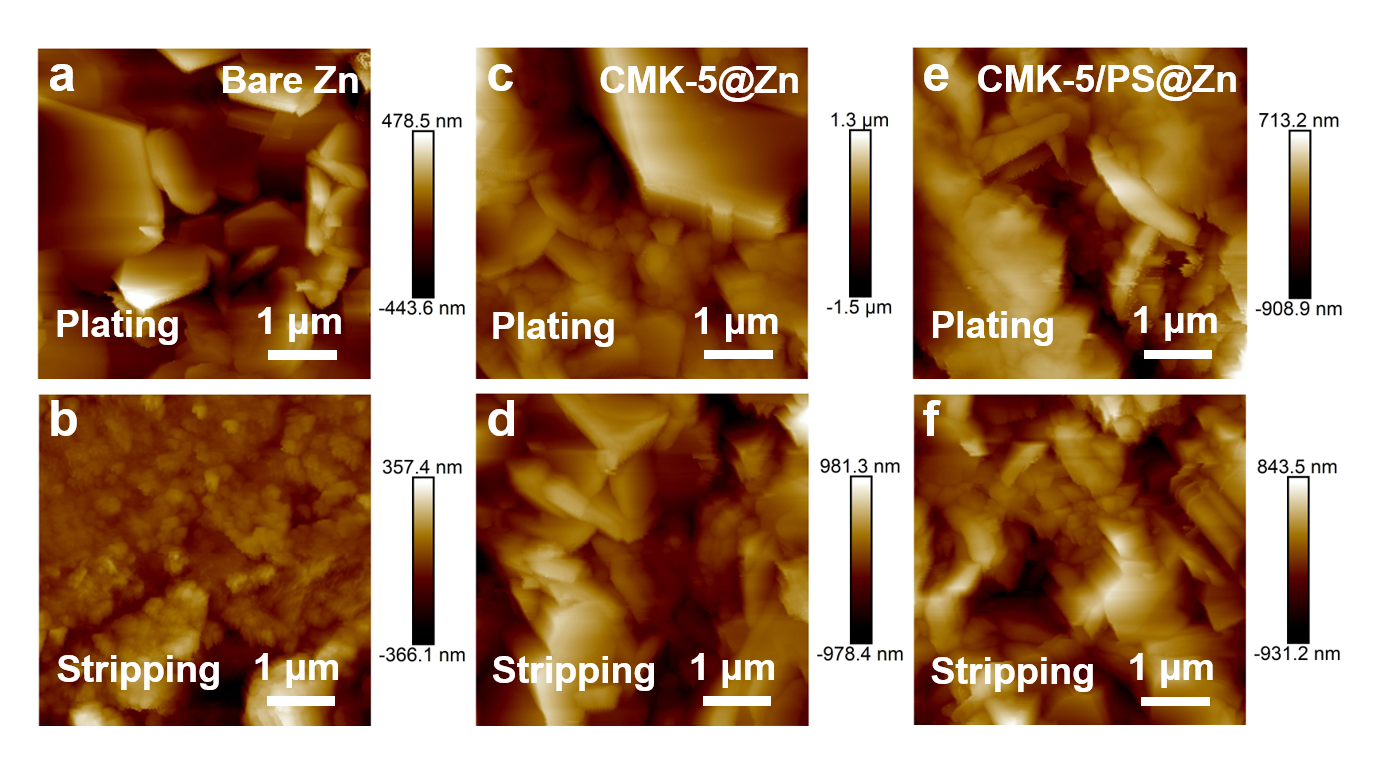
**

**Figure S31.** AFM images of **a.** and **b.** bare Zn, **c.** and **d.** CMK-5@Zn, **e.** and **f.** CMK-5/PS@Zn anode at the first Zn plating/stripping state.

**
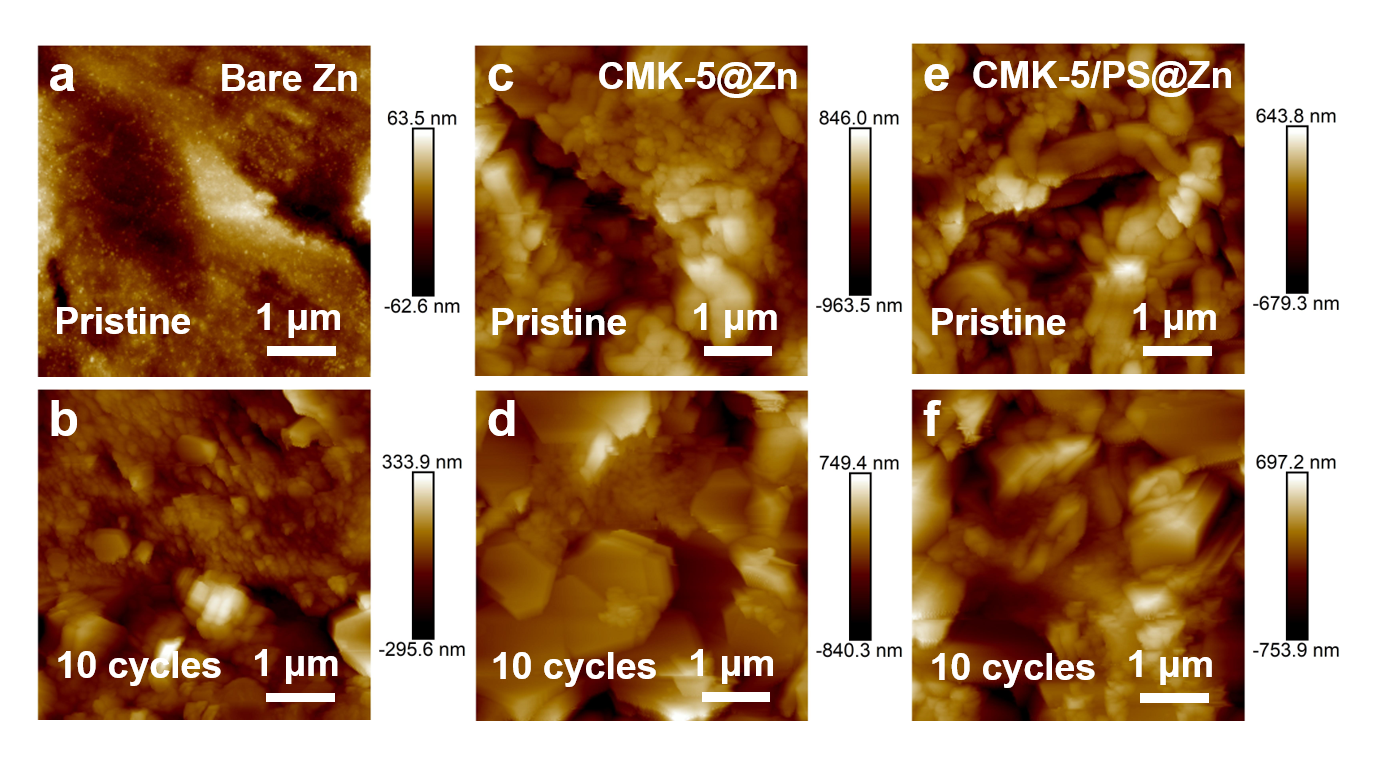
**

**Figure S32.** AFM images of **a.** and **b.** bare Zn, **c.** and **d.** CMK-5@Zn, **e.** and **f.** CMK-5/PS@Zn anode after 10 times Zn^2+^ electroplating/stripping.

**
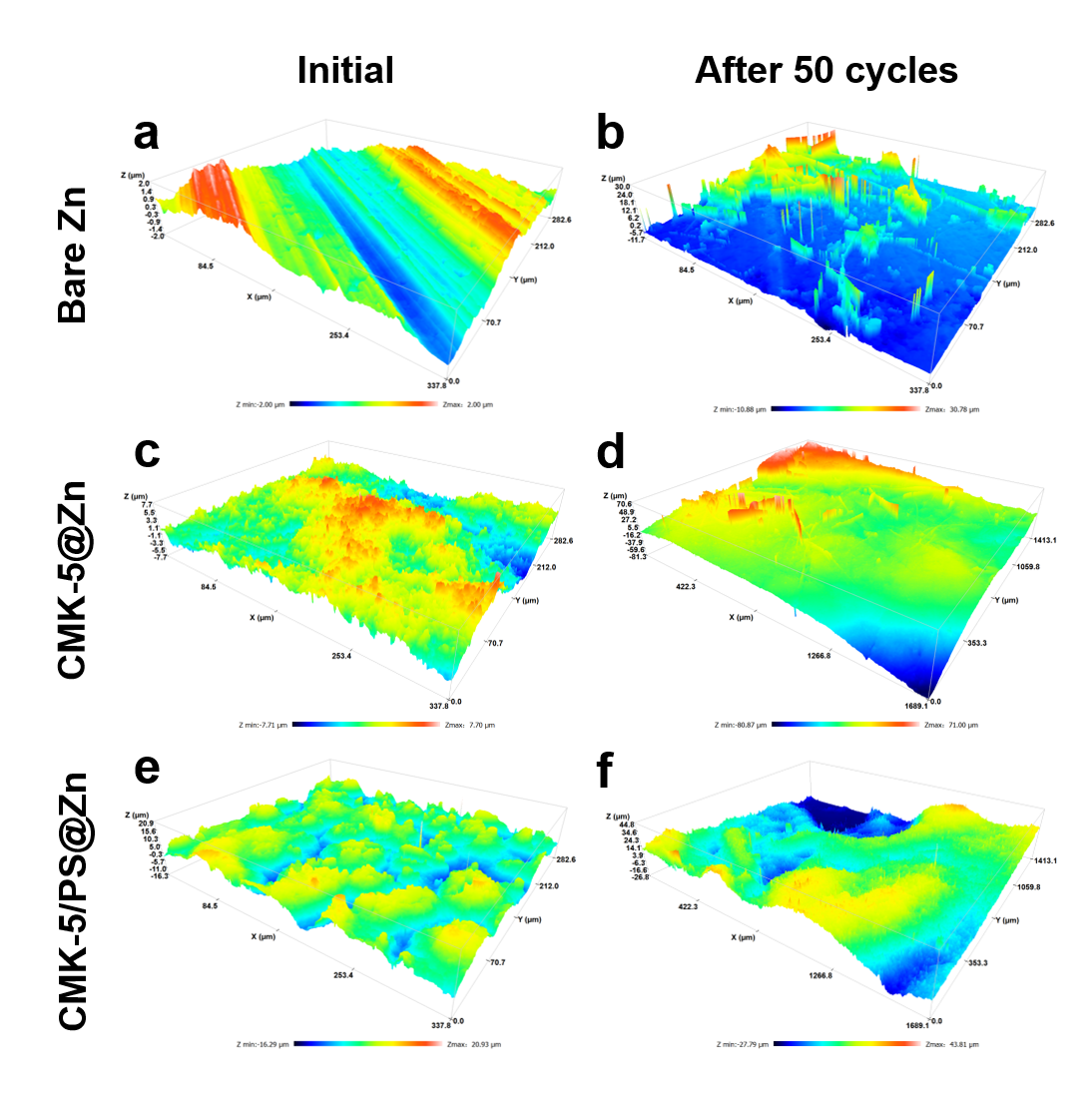
**

**Figure S3****3.** 3D laser confocal microscopy images of **a-b.** bare Zn, **c-d.** CMK-5@Zn and **e-f.** CMK-5/PS@Zn anode at initial and after 50 cycles state.

Three-dimensional laser confocal microscopy was used to test the topography of the three negative electrodes at initial state. After coating the surface of Zn foil with CMK-5/PS, the overall roughness was reduced and a relatively smooth morphology was exhibited.

**
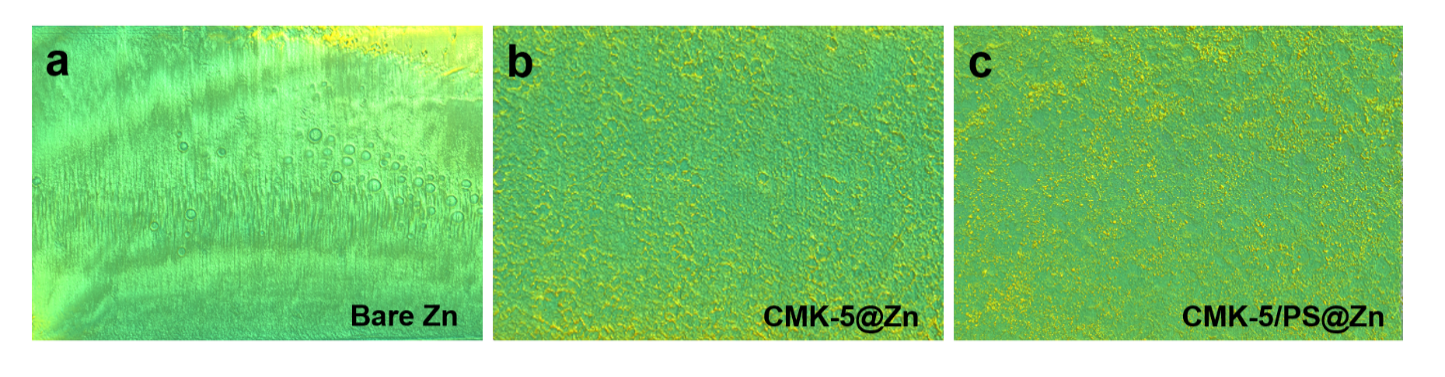
**

**Figure S34.** The top views corresponding to the 3D rendering images of **a.** bare Zn, **b.** CMK-5@Zn and c. CMK-5/PS@Zn.


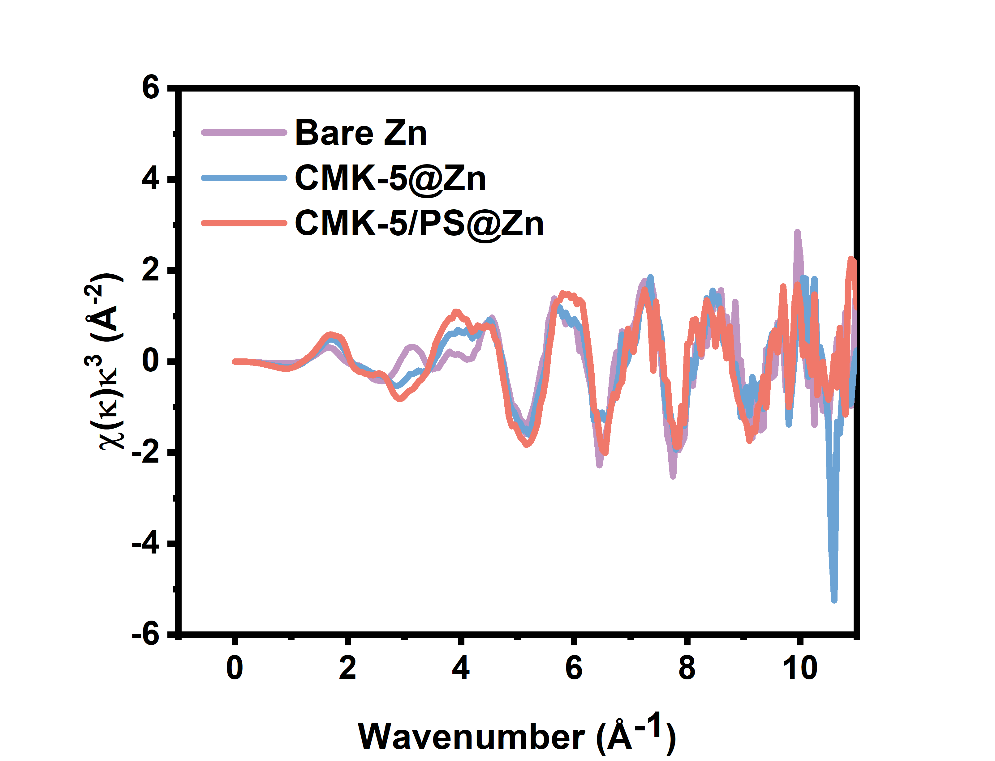


**Figure S35.** The Synchrotron X-ray absorbing near-edge structures of bare Zn, CMK-5@Zn and CMK-5/PS@Zn for K-space.


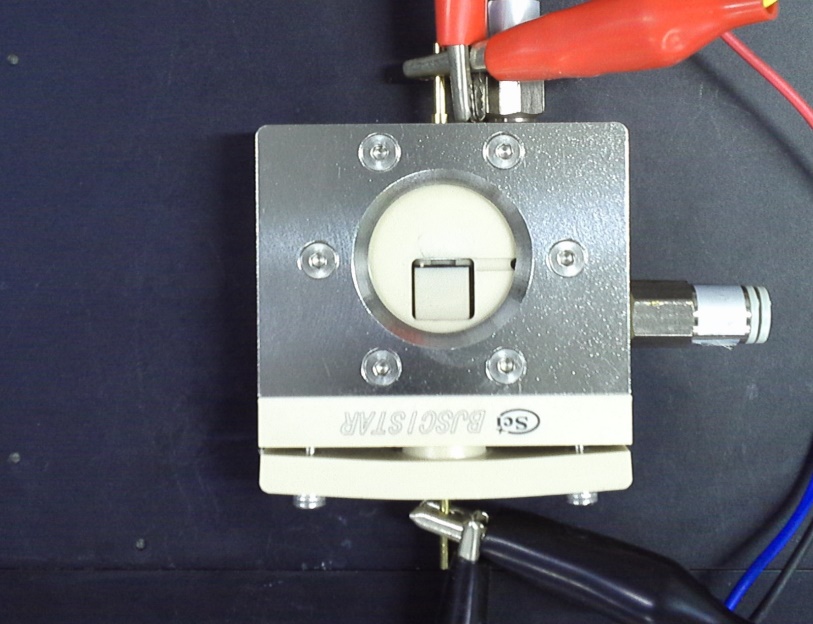


**Figure S36.** Digital images of the combined *in-situ* optical cell.

**
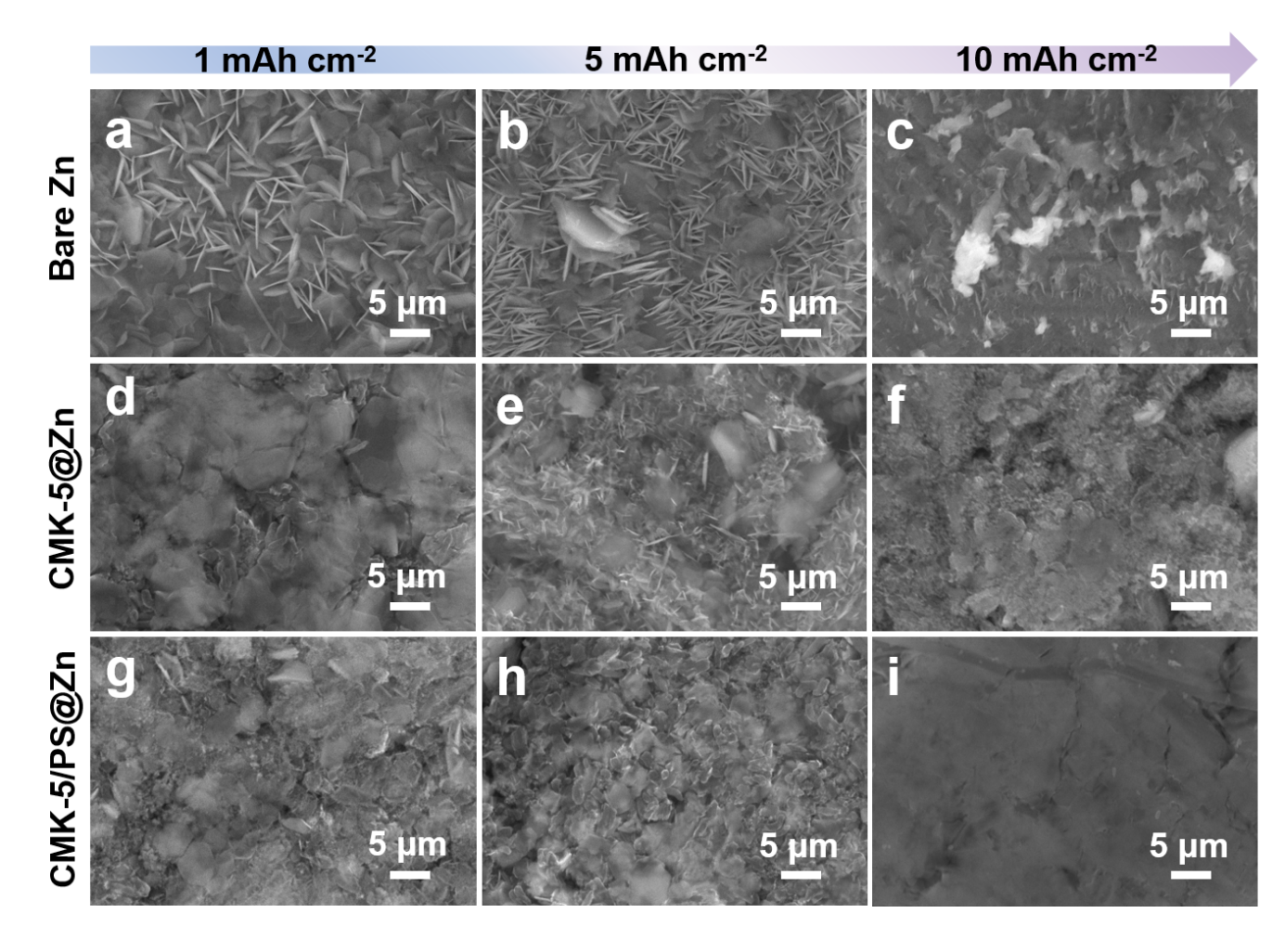
**

**Figure S37.** SEM images of **a-c**. bare Zn, **d-f.** CMK-5@Zn, **g-i.** CMK-5/PS@Zn deposited at different capacities (1, 5, 10 mAh cm^-2^).

When the deposition capacity was 1mAh cm^-2^, flake aggregates appeared in the bare Zn anode, and when the deposition capacity increased to 10mAh cm^-2^, flake aggregates formed large dendrites. CMK-5@Zn anode is relatively smooth during the whole deposition process. In contrast, it is obvious that the CMK-5/PS@Zn anode is completely covered by Zn and forms a very smooth surface with the increase of deposition capacity.

**
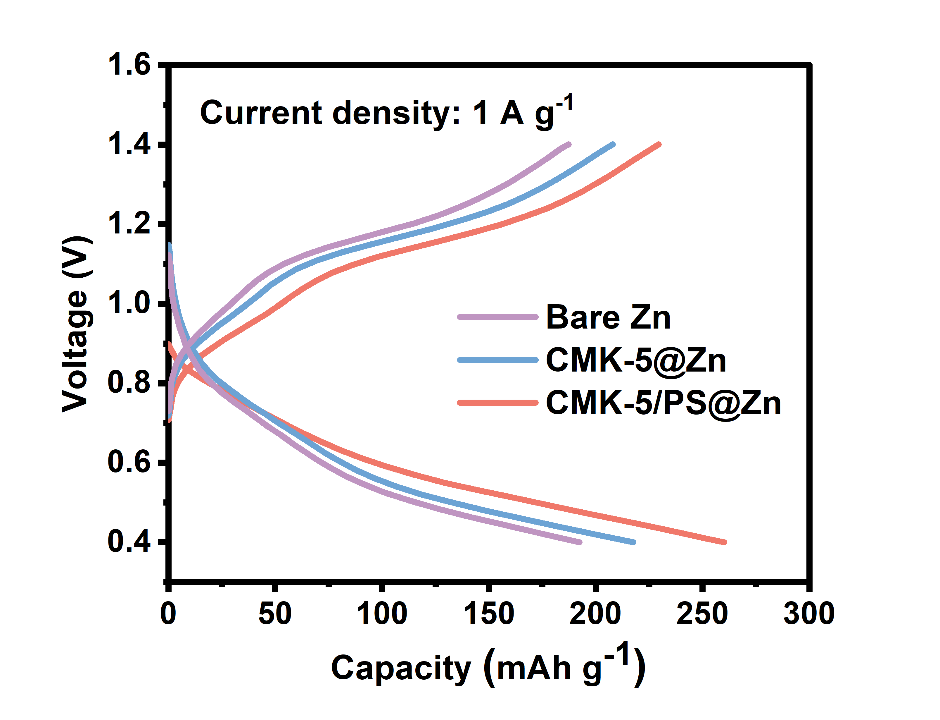
**

**Figure S38.** Initial constant current charge-discharge curve at a current density of 1 A g^-1^.

**
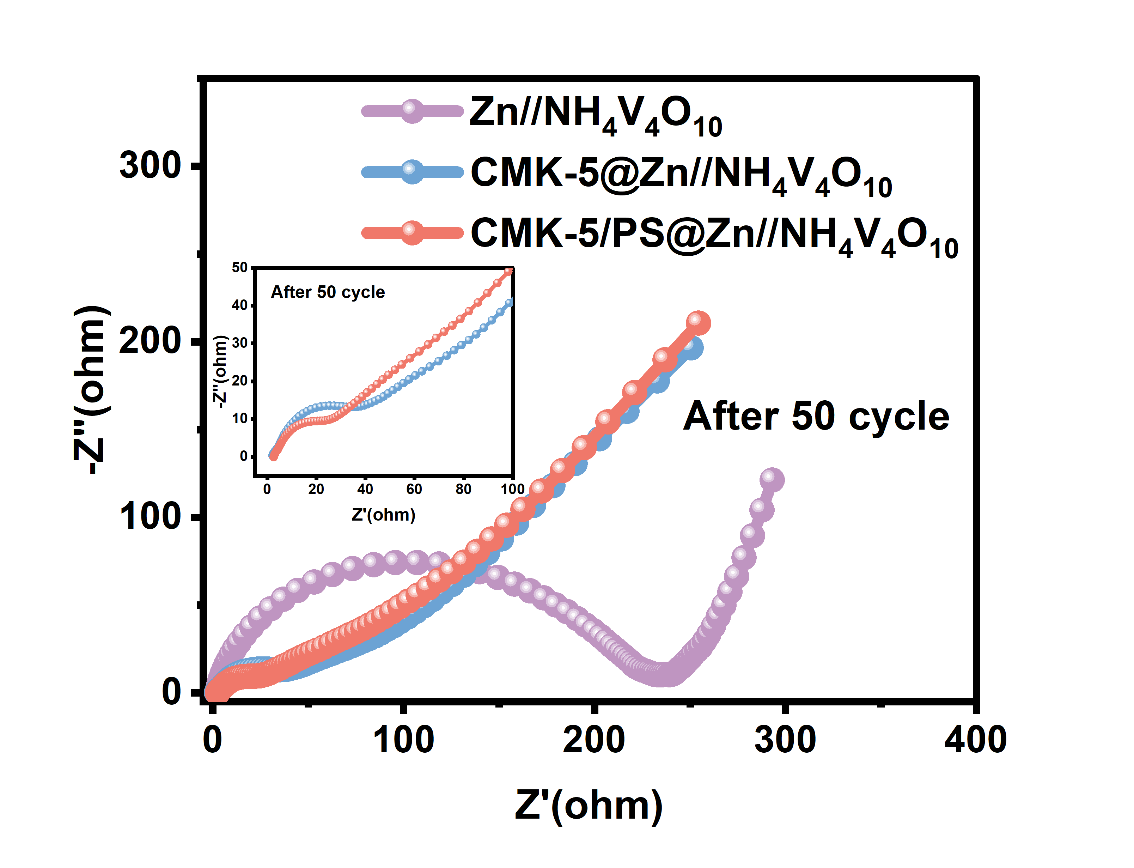
**

**Figure S39.** The impedance of the full cell after 50 cycles.

**
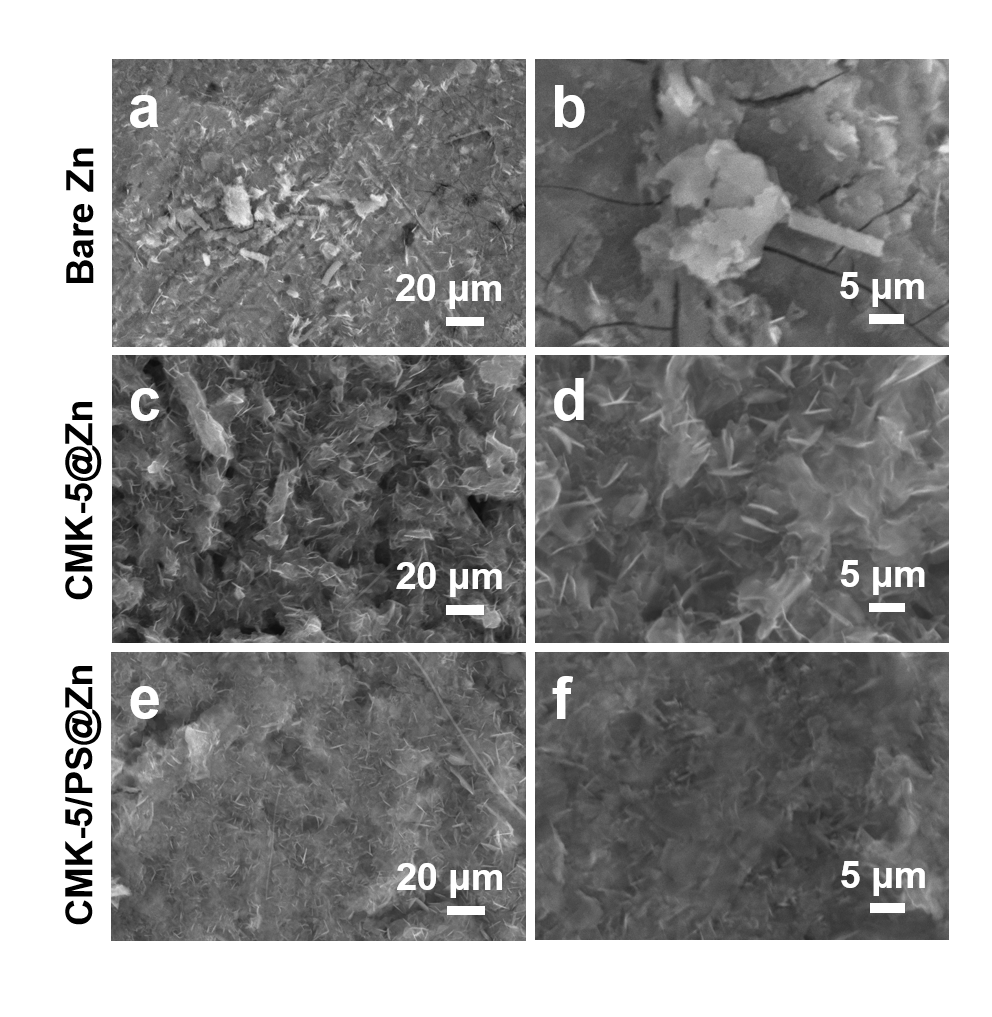
**

**Figure S40.** The SEM of **a-b.** bare Zn, **c-d.** CMK-5@Zn, **d-e.** CMK-5/PS@Zn anode at the full cell after 50 cycles.

**
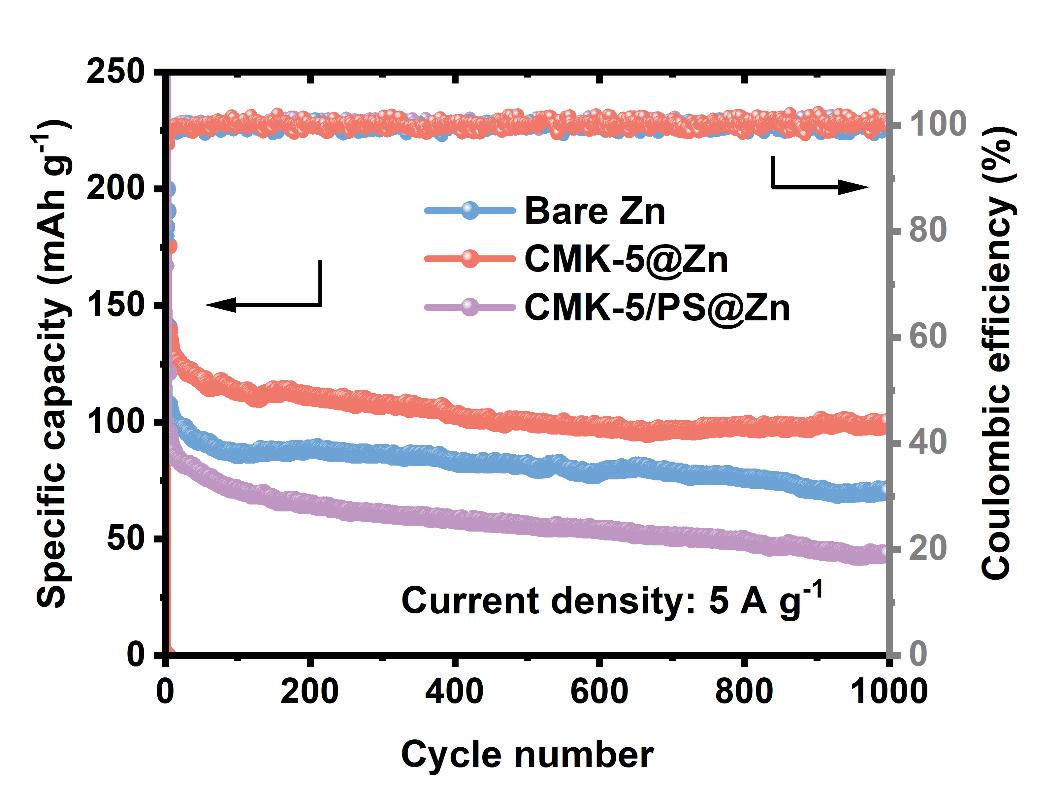
**

**Figure S41.** Comparison of full battery performance at current density of 5 A g^-1^.


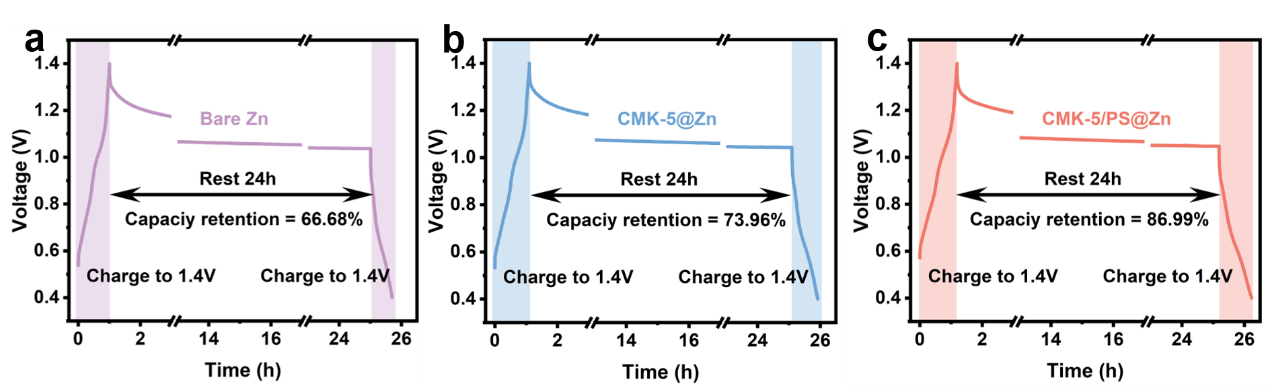


**Figure S42.** Self-discharge curves of **a.** Zn||NH_4_V_4_O_10_, **b.** CMK-5@Zn||NH_4_V_4_O_10_ and **c.** CMK-5/PS@Zn||NH_4_V_4_O_10_ full cell.

**Supporting Tables**

**Table S1.** Physiochemical properties of CMK-5/PS and CMK-5.

| Sample | S_BET_ (m^2/^g) | | Pore volume (cm^3/^g) | Pore size (nm) |
| --- | --- | --- | --- | --- |
| CMK-5 | | 1836.8674 | 1.993989 | 2.9; 4.6 |
| CMK-5/PS | | 1667.2092 | 1.802470 | 3.1; 4.6 |

The nitrogen adsorption-to-desorption isotherms of CMK-5/PS and CMK-5 show a characteristic type IV curve, with obvious capillary condensation at P/P_0_=0.40-0.65, indicating uniform mesoporous presence in the prepared CMK-5/PS products. The figure shows the corresponding pore size distribution curve. Two peaks appear at about 2.9 nm and 4.6 nm, corresponding to the mesopore formed after removing the silica template and the internal pore of carbon nanotubes, respectively, which are in good agreement with TEM results. These results indicate that CMK-5/PS has a hollow mesostructure similar to CMK-5. Notably, BET estimates CMK-5/PS has a high specific surface area (1667.2092 m^2^/g) and a high pore volume (1.802470 cm^3^/g) (Table S1). The results showed that the introduction of the organic layer maintained the unique bimodal pore size structure of CMK-5 and provided more active sites for the deposition of Zn^2+^.

**Table S2.** Comparison of the cycling performance recently reported Zn anode via the Carbon protective layer.

| **Anode** | **Voltage hysteresis**  **(mV)** | **Current density**  **(mA cm**^–^**^2^)** | **Capacity**  **(mAh cm**^–^**^2^)** | **Life (h)** | **References** |
| --- | --- | --- | --- | --- | --- |
| **CMK-5/PS@Zn** | **21.7** | **1** | **0.5** | **8500** | **This work** |
| **CMK-5/PS@Zn** | **16.7** | **0.5** | **0.5** | **4500** | **This work** |
| **CMK-5/PS@Zn** | **18.6** | **1** | **1** | **1200** | **This work** |
| **CMK-5/PS@Zn** | **28.8** | **4** | **2** | **1500** | **This work** |
| **CMK-5/PS@Zn** | **72.5** | **10** | **1** | **1000** | **This work** |
| NOC@Zn | ~30 | 1 | 1 | 3040 | ^[13]^ |
| c-PLA@Zn | ~50 | 1 | 1 | 1600 | ^[14]^ |
| Sn@NHCF-Zn | 21 | 1 | 1 | 370 | ^[15]^ |
| BCK-7@Zn | ~50 | 1 | 0.15 | 950 | ^[16]^ |
| Zn@MGs | ~100 | 10 | 1 | 600 | ^[17]^ |
| Zn@graphene | 32 | 0.2 | 0.2 | 4000 | ^[18]^ |
| Zn@Cu-Ps/EG | 92.6 | 5 | 5 | 3250 | ^[19]^ |
| Sn@NCNFs@Zn | ~40 | 1 | 1 | 3500 | ^[20]^ |
| CF-Cu@Zn | 14.6 | 0.5 | 0.25 | 2200 | ^[21]^ |
| Zn@C-5 | 32 | 1 | 1 | 880 | ^[22]^ |
| Zn@F-CDs | ~40 | 1 | 1 | 3500 | ^[23]^ |

**Table S3.** The corresponding impedances and currents before and after polarization process.

| Anode | ΔV (mV) | *R_0_* (Ω) | *R_s_* (Ω) | *I_0_* (μA) | *I_s_* (μA) | *t_Zn_^2+^* |
| --- | --- | --- | --- | --- | --- | --- |
| CMK-5/PS@Zn | 10 | 706 | 744 | 22.9 | 18.2 | 0.65 |
| CMK-5@Zn | 10 | 761 | 899 | 21.2 | 18.5 | 0.57 |
| Bare Zn | 10 | 2691 | 3000 | 24.7 | 18.6 | 0.43 |

**Table S4.** The conductivity was measured using a four-probe resistance meter.

| Anode | (Ω·cm) | (Ω·cm) | (Ω·cm) | (Ω·cm) | (Ω·cm) | Average |
| --- | --- | --- | --- | --- | --- | --- |
| CMK-5/PS | 73.45 | 61.15 | 85.96 | 78.88 | 69.76 | 73.84 |
| CMK-5 | 271.92 | 229.65 | 264.33 | 302.82 | 254.57 | 264.66 |

The electronic conductivity of CMK-5/PS and CMK-5 powders was measured using a four-probe powder resistivity tester equipped with a high resistance meter (ST2255).

**Table S5.** Electrochemical performance of the modified Zn anodes in full cells.

| **Anode** | **Cathode** | **Current density** **(A g**^-^**^1^)** | **reversible capacity (mAh g^-1^)** | **Cycle number** | **capacity retention** | **References** |
| --- | --- | --- | --- | --- | --- | --- |
| **CMK-5/PS@Zn** | **NH_4_V_4_O_10_** | **1** | **213.8** | **1000** | **98.2%** | **This work** |
| **CMK-5/PS@Zn** | **NH_4_V_4_O_10_** | **5** | **99.4** | **1000** | **85.5%** | **This work** |
| **CMK-5/PS@Zn** | **NH_4_V_4_O_10_** | **10** | **61.86** | **2000** | **50.7%** | **This work** |
| Zn@MGs | NaV_3_O_8_⋅1.5H_2_O | 5 | 104 | 1370 | 46.2% | ^[24]^ |
| 502-coated Zn | V_2_O_5_⋅1.6H_2_O | 1 | 95.4 | 550 | 59.3% | ^[25]^ |
| Zn-G | V_2_O_5_⋅xH_2_O | 5 | 98 | 1500 | 84% | ^[26]^ |
| 3D Zn-0.1 | NVO | 5 | 100.1 | 2000 | 73.7% | ^[27]^ |
| ZnBTC@Zn | NHVO | 3 | 91.5 | 10000 | 65.8% | ^[28]^ |
| Zn@ZSO | NH_4_V_4_O_10_ | 5 | 79 | 1000 | 80.1% | ^[29]^ |
| CFx-PVDF@Zn | NH_4_V_4_O_10_ | 1 | 175.2 | 1000 | 61.7% | ^[30]^ |
| CFx-PVDF@Zn | NH_4_V_4_O_10_ | 5 | 74.6 | 3000 | 50.7% | ^[30]^ |

**Legends for Movies S1, S2, S3 and S4.**

**Movie S1.** Comsol simulation of concentration fields inside and outside channels of Zn^2+^ at 0-10s deposited by C@Zn anode.

**Movie S2.** Comsol simulates the growth of zinc dendrites inside and outside pores during deposition of Zn^2+^ at 0-10s by C@Zn anode.

**Movie S3.** Comsol simulation of concentration fields inside and outside channels of Zn^2+^ at 0-10s deposited by Janus-C@Zn anode.

**Movie S4.** Comsol simulates the growth of zinc dendrites inside and outside pores during deposition of Zn^2+^ at 0-10s by Janus-C@Zn anode.

**References**

[1] Z. Liang, Y. Peng, X. Zhang, K. Cao, W. Xiao, D. Gu, *Chin. Chem. Lett.* **2023**, 34, 108054.

[2] C. Zhou, L. Shan, Q. Nan, J. Zhang, Z. Fan, B. Tang, J. Li, J. Yang, H. Zhang, Z. Kang, X. Tian, X. Shi, *Adv. Funct. Mater.* **2024**, 34, 2312696.

[3] G. Kresse, J. Hafner, *Phys. Rev. B* **1994**, 49, 14251.

[4] K. B. J.P. Perdew, *Phys. Rev. Lett.* **1996**, 77, 3865.

[5] D. J. G. Kresse, *Phys. Rev. B* **1999**, 59, 3.

[6] P. E. Blochl, *Phys Rev B Condens Matter* **1994**, 50, 17953.

[7] Tom K. Woo, *J. Phys. Chem. B* **1997**, 101, 40.

[8] C. Jarzynski, *Phys. Rev. Lett.* **1997**, 78, 14.

[9] L. Bokobza, J.-L. Bruneel, M. Couzi, *Chem. Phys. Lett.* **2013**, 590, 153.

[10] R. Yuan, Y. Guo, I. Gurgan, N. Siddique, Y.-S. Li, S. Jang, G. A. Noh, S. H. Kim, *Carbon* **2025**, 238, 120214.

[11] X. Zhou, B. Wen, Y. Cai, X. Chen, L. Li, Q. Zhao, S. L. Chou, F. Li, *Angew. Chem. Int. Ed.* **2024**, 63, e202402342.

[12] S. Chen, M. Zhang, P. Zou, B. Sun, S. Tao, *Energy Environ. Sci.* **2022**, 15, 1805.

[13] X. Yang, J. Lv, C. Cheng, Z. Shi, J. Peng, Z. Chen, X. Lian, W. Li, Y. Zou, Y. Zhao, M. H. Rümmeli, S. Dou, J. Sun, *Adv. Sci.* **2022**, 10, 2206077.

[14] M. Abouali, S. Adhami, S. A. Haris, R. Yuksel, *Angew. Chem. Int. Ed.* **2024**, 63, e202405048.

[15] H. Yu, *Science* **2022**, 8, eabm5766.

[16] W. Zheng, H. Xie, L. Zhu, H. Zhou, K. Zhang, *J. Energy Storage* **2024**, 76, 109808.

[17] S. You, D.-S. Liu, M. Ye, Y. Zhang, Y. Tang, X. Liu, C. Chao Li, *Chem. Eng. J.* **2023**, 454, 139907.

[18] M. Wang, Q. Wang, H. Yao, F. Su, Z. Shan, H. Shen, T. Liu, J. Zhao, C. Ding, *J. Alloys Compd.* **2023**, 947, 169678.

[19] G. Chen, Z. Sang, J. Cheng, S. Tan, Z. Yi, X. Zhang, W. Si, Y. Yin, J. Liang, F. Hou, *Energy Storage Mate.* **2022**, 50, 589.

[20] B. Wang, J. Hao, H. Xu, M. Sun, C. Wu, W. Qin, X. Wu, Q. Wei, *ACS Appl. Mater. Interfaces* **2024**, 16, 46879.

[21] H. Wang, Y. Chen, H. Yu, W. Liu, G. Kuang, L. Mei, Z. Wu, W. Wei, X. Ji, B. Qu, L. Chen, *Adv. Funct. Mater.* **2022**, 32, 2205600.

[22] J. Wang, H. Zhang, L. Yang, S. Zhang, X. Han, W. Hu, *Angew. Chem. Int. Ed.* **2024**, 63, e202318149.

[23] Z. Ge, L. Xu, Y. Xu, J. Wu, Z. Geng, X. Xiao, W. Deng, G. Zou, H. Hou, X. Ji, *Nano Energy* **2024**, 119, 109053.

[24] S. You, D.-S. Liu, M. Ye, Y. Zhang, Y. Tang, X. Liu, C. Chao Li, *Chem. Eng. J.* **2023**, 454, 139907.

[25] Z. Cao, X. Zhu, D. Xu, P. Dong, M. O. L. Chee, X. Li, K. Zhu, M. Ye, J. Shen, *Energy Storage Mater.* **2021**, 36, 132.

[26] Z. Li, L. Wu, S. Dong, T. Xu, S. Li, Y. An, J. Jiang, X. Zhang, *Adv. Funct. Mater.* **2021**, 31, 2006495.

[27] X. Fu, L. Wang, G. Li, Y. Su, J. Wang, W. Yu, X. Dong, D. Liu, X. Wang, *Electrochim. Acta* **2025**, 536, 146800.

[28] Q. Wen, T. Chen, C. Sun, Y. Chen, R. Ji, R. Cui, H. Chen, L. Tang, J. Zhang, X. Zhang, J. Zheng, *J. Mater. Chem. A,* **2025**, 13, 26593.

[29] H. Peng, Y. Fang, J. Wang, P. Ruan, Y. Tang, B. Lu, X. Cao, S. Liang, J. Zhou, *Matter* **2022**, 5, 4363.

[30] S. Song, J. Chen, A. Wang, M. Shen, F. Shen, Q. Lu, J. Xu, Z. Lin, X. Han, *Energy Fuels* **2025**, 39, 19016.
